# Endosymbiont Dominated Bacterial Communities in a Dwarf Spider

**DOI:** 10.1371/journal.pone.0117297

**Published:** 2015-02-23

**Authors:** Bram Vanthournout, Frederik Hendrickx

**Affiliations:** 1 Department of Bioscience, Aarhus University, Aarhus-C, Denmark; 2 Royal Belgian Institute of Natural Sciences, Brussels, Belgium; 3 Terrestrial Ecology Unit (TEREC), Biology Department, Ghent University, Gent, Belgium; University of Vienna, AUSTRIA

## Abstract

The microbial community of spiders is little known, with previous studies focussing primarily on the medical importance of spiders as vectors of pathogenic bacteria and on the screening of known cytoplasmic endosymbiont bacteria. These screening studies have been performed by means of specific primers that only amplify a selective set of endosymbionts, hampering the detection of unreported species in spiders. In order to have a more complete overview of the bacterial species that can be present in spiders, we applied a combination of a cloning assay, DGGE profiling and high-throughput sequencing on multiple individuals of the dwarf spider *Oedothorax gibbosus*. This revealed a co-infection of at least three known (*Wolbachia, Rickettsia* and *Cardinium*) and the detection of a previously unreported endosymbiont bacterium (*Rhabdochlamydia*) in spiders. 16S rRNA gene sequences of *Rhabdochlamydia* matched closely with those of *Candidatus* R. porcellionis, which is currently only reported as a pathogen from a woodlouse and with *Candidatus* R. crassificans reported from a cockroach. Remarkably, this bacterium appears to present in very high proportions in one of the two populations only, with all investigated females being infected. We also recovered *Acinetobacter* in high abundance in one individual. In total, more than 99% of approximately 4.5M high-throughput sequencing reads were restricted to these five bacterial species. In contrast to previously reported screening studies of terrestrial arthropods, our results suggest that the bacterial communities in this spider species are dominated by, or even restricted to endosymbiont bacteria. Given the high prevalence of endosymbiont species in spiders, this bacterial community pattern could be widespread in the Araneae order.

## Introduction

It has become clear that many arthropods harbour a wealth of symbiotic bacteria exerting a strong effect on host adaptation and, hence, host evolution [1–6]. Though recent advances in molecular methods have allowed for a comprehensive quantification of microbial communities in a diverse set of species (e.g. [7–9]), the majority of arthropod groups still remain largely unexplored. This is particularly the case for spiders, for which only little research on their microbial community has currently been performed.

The few studies on microbial assemblages associated with spiders concentrated on the medical importance of spiders as vectors of potentially human pathogenic bacteria. Here, emphasis has been put on the general internal and external bacterial community of the spider [10,11], human serum and blood after presumed spider bites [12] and the presence of pathogenic bacteria associated with venom and fangs [13,14].

On the other hand, microbial investigations in spiders focussed on infection by endosymbiont bacteria. These maternally inherited bacteria that reside obligatorily in the cells or intercellular lumen of their host may profoundly alter host reproduction by killing male offspring, feminizing genetic males and inducing parthenogenesis and cytoplasmic incompatibility [15–17]. One of the earliest accounts of endosymbiont infections in spiders were made by microscope studies that report *Rickettsia* and *Chlamydia* like bacteria [18–22]. However, electron microscopy is limited in the observation of bacterial species as the positive identification is based on phenotypic features. A more systematic approach through PCR based screening of endosymbionts, with particular emphasis on the endosymbionts *Wolbachia, Rickettsia, Spiroplasma* and *Cardinium*, showed that spiders may show a remarkable diversity and high prevalence of cytoplasmic bacteria [23–27]. Currently, up to six different bacterial taxa that potentially affect host reproduction have been detected so far (i.e. *Wolbachia, Rickettsia, Spiroplasma ixodetis, Spiroplasma poulsonii, Cardinium* and *Arsenophonus* [24,28]). In few cases, these endosymbionts appeared to have a pronounced effect on their spider hosts’ biology by manipulating host reproduction [28–30] as well through affecting dispersal behaviour [31].

Quantification of bacterial species in these studies are based on directed searches that target only a predefined set of putative reproductive manipulators. These methods are therefore less suitable to provide a more comprehensive view on the complete endosymbiont community present in many arthropod groups. However, with the advent of high-throughput sequencing techniques this can readily be achieved by employing a metagenomic approach.

In this paper we aim to determine the bacterial diversity in the dwarf spider *Oedothorax gibbosus*. Previous work showed that two populations of this spider are infected by at least three endosymbiont species, i.e. *Wolbachia* (belonging to clade G), *Rickettsia* and *Cardinium*. Of these, only *Wolbachia* was found to affect reproduction causing a female biased sex ratio by killing male embryos [28]. Remarkably, some females produced highly distorted female biased sex ratios in the absence of *Wolbachia* (unpublished results), suggesting that an additional factor influences sex ratio in this species. To fully understand the different agents potentially affecting sex ratio there is a clear need of a comprehensive identification of the microbial community.

We tackle this problem by first characterizing and identifying the bacterial community of this species using a combination of high-throughput sequencing, cloning and DGGE profiling of 16S rDNA amplicons. Second, we test the prevalence of newly identified bacteria in this species in multiple individuals of both sexes by means of species specific PCR screenings.

## Material and Methods

### Sample origin and DNA extraction

Individuals of *Oedothorax gibbosus* used in the molecular analysis were sampled by hand in two different populations in Belgium i.e. Damvallei (DAM: 51°03’25.60”N, 3°49’51.13”E)) and Walenbos (WAL: 50°55’32.49”N, 4°51’48.91”E, permissions for field collections: Walenbos: Belgian Nature and Forest Agency, Damvallei: Natuurpunt). *Oedothorax gibbosus* is not an endangered or protected species.

Although mostly field captured specimens were used for molecular analysis, we also included specimens that were bred from wild caught females from the WAL population for six consecutive generations (F6 generation). One of these lines, further referred to as *Wol+*, was previously shown to be infected with *Wolbachia, Rickettsia* and *Cardinium* and previously used to investigate the effect of *Wolbachia* infection on sex ratio (see [28]) (average sex ratio: 0.25 ± 0.04 (n = 797 offspring)). The second maternal line, further referred to as *Wol-*, was even more female biased and infected with *Rickettsia* and *Cardinium*, but not with *Wolbachia* (average sex ratio: 0.05 ± 0.02 (n = 611 offspring)).

DNA of whole individuals was extracted using the NucleoSpin Tissue DNA extraction kit (Macherey-Nagel) following the manufacturers recommended protocol.

### Identification and characterization of the bacterial community

We first attempted to obtain a complete picture of the dominant bacterial species that can be found in *Oedothorax gibbosus*. To achieve this, we applied the following molecular methods on either pooled or individual samples.

Cloning of bacterial 16S rRNA

Two individual females of the *Wol-* maternal line and two individual females of the *Wol+* maternal line were used for the cloning assay (Table 1). 16S rRNA gene sequences were amplified using the universal primers F45 and R1242 [32]. PCR conditions were as following: initial denaturation at 95°C for 2 min, followed by 35 cycles of denaturation at 94°C for 30 s, annealing at 55°C for 30 s, extension at 72°C for 90 s and a final extension at 72°C during 5 min. PCR amplicons were ligated into the pCR II-TOPO vector (Invitrogen) and transferred into TOP10 chemically competent cells (Invitrogen) according to the manufacturers recommended protocol. Cloned sequences were reamplified using the M13F and M13R primers (Invitrogen) and presence of the insert was checked by gel electrophoresis. Inserts with bands of expected size were sequenced using BigDye v.1.1 Terminator Sequencing mix and run on an ABI 3130 automated sequencer. Identification of the bacteria was based on BLAST searches against both the NCBI nucleotide collection and the NCBI 16S rRNA database (bacteria and archaea).

**Table 1:** Overview of the different individuals (D: Damvallei, W: Walenbos; Wol individuals originate from two Walenbos matrilines) used in the cloning assay (Cloning), V3 high-throughput sequencing (HTS-V3), V4 high-throughput sequencing (HTS-V4) and Denaturating Gradient Gel Electrophoresis (DGGE) and the different bacteria found in each assay (Ac = *Acinetobacter*, Ca = *Cardinium*, Ri = *Rickettsia*, Rh = *Rhabdochlamydia*, Wo = *Wolbachia*).

Individual	Origin	Cloning	HTS-V3	HTS-V4	DGGE
Wol-.01	lab		Ri, Ca, Rh	Ri, Ca, Rh	Ri
Wol-.02	lab	Ri, Rh			Ri
Wol-.03	lab	Ri, Rh			
Wol+.01	lab		Wo, Ri, Ca, Rh	Wo, Ri, Ca, Rh	Wo, Ri
Wol+.02	lab				Wo, Ri
Wol+.03	lab	Wo, Ri			Wo, Ri
Wol+.04	lab	Wo, Ri			Wo, Ri
D160	wild			Wo, Ri, Ca, Ac	Ri
D023	wild				Wo, Ri
D043	wild				Wo, Ri
D026	wild				Ac
D100	wild				Ri
D121	wild				
D306	wild				
D054	wild				
D057	wild				
D327	wild				
W121	wild			Wo, Ri, Ca, Rh	Ri
W102	wild				Ri
W202	wild				Wo, Ri
W220	wild				Ri
W011	wild				Ri
W162	wild				
W216	wild				
W312	wild				
W186	wild				
W201	wild				
W208	wild				Wo

High-throughput sequencing

Two individual females, one of the *Wol+* and one of the *Wol-* maternal line, and two pooled samples, one from WAL (n = 10 females) and one from DAM (n = 10 females) were used for high-throughput sequencing (Table 1). For the pooled samples, DNA of individual extracts was measured with a fluorometric method (Qubit, Invitrogen) and equimolar amounts of DNA were combined from each of the 10 individuals into a single sample. We targeted the 16S rRNA V4 region for all four samples using the primers F563 and R802 (RDP website: http://pyro.cme.msu.edu/pyro/help.jsp) and the V3 region with the primers F338 and R534 (V3; [33]) for the two individual samples only. Including both the V3 and V4 regions accounts for differences between samples based on differential primer specificity. Primers were indexed with a sample specific barcode and amplicons were paired-end sequenced for 100 cycles at 1/8 of a single Illumina HiSeq2000 lane (Baseclear NV, The Netherlands).

Raw reads were subjected to an initial screening and only high quality reads with correct barcode, correct primer sequence and no ambiguous base pairs were retained for further downstream analysis (1.898.581 and 3.773.370 paired-end reads for V3 and V4 respectively).

We first combined both 100bp paired-end reads into a single sequence. For the V3 region, which spans <200bp, overlap is expected between the complementary read ends and we merged the overlapping reads with FLASH [34]. For the V4 region, which spans a region of approximately 238bp, both reads were merged while inserting a series of 38N’s between both read ends by means of a home-made Python script “ConcatenateNonoverlaps.py” (S1 Information). An initial screening of the V3 data showed considerable contamination with the 18S rRNA of the spider. We identified and removed these contaminant sequences with the standalone version of DeconSeq [35] by retaining sequences with hits to Bacterial (2,206 unique genomes and 73,337 whole genome sequences) and Archaeal (155 unique genomes) genomes and removing reads with hits to the 18S rRNA sequence of *Oedothorax gibbosus*. A total of 994,207 V3 sequences were retained.

We used MOTHUR v.1.26.0 [36] for further downstream analysis, data clean-up and data reduction. First, replicate sequences were eliminated to reduce the entire dataset. Remaining sequences were then aligned to the SILVA bacterial reference alignment (downloaded from http://www.mothur.org/wiki/Silva_reference_files, accessed 29/06/2013) using the Needleman alignment method and a *k*-mer size = 8. Sequences that did not align within the expected range were removed. The remaining sequences were preclustered into groups of sequences with maximal 2bp differences. Next, we identified chimeric sequences with ChimeraSlayer as well as UChime [37] as implemented in MOTHUR, using both the SILVA reference alignment as well as the original sequences (taking into account their frequency) as a template. Chimeric sequences constituted 132 (0.8%; V3) and 36,252 (27.2%; V4) of the preclustered sequences. The reduced dataset consisted of 18,863 (V3) and 96,789 (V4) unique sequences, representing 887,125 (V3) and 3,645,675 (V4) of the original sequences.

After data preprocessing, a phylotype analysis was conducted wherein sequences were first assigned a taxonomy and subsequently clustered according to their taxonomic identity. Taxonomic assignment was based on the Wang method [38] using a *k*-mer size = 8 and taxonomic classification was based on the Greengenes taxonomy as this database contained most detailed taxonomic classification of the endosymbiont species obtained from cloning analysis. We further performed taxonomic assignment of each cluster by comparing the most abundant sequence from each cluster against both the type and non-type and cultured and uncultured strain bacterial database using the Ribosomal Database Project (RDP; http://rdp.cme.msu.edu/, accessed 02/07/2013)[39] (See S2 and S3 Informations for the V3 region, and S4 and S5 Informations for the V4 region). A detailed description of the bioinformatics steps and MOTHUR commands to perform the analyses are given in S6 Information. Assembled reads were submitted to GenBank and accessible as BioSamples SAMN02903876 (*Wol-*V3); SAMN02903877 (*Wol+*_V3); SAMN02903878 (*Wol-*V4); SAMN02903879 (*Wol+*_V4); SAMN02903880 (WAL) and SAMN02903881 (DAM).

Low number of reads were observed corresponding to *Wolbachia* in the *Wol-* matriline and *Rhabdochlamydia* in the DAM pooled samples for the V4 region. These most likely constitute contamination due to the very low number of reads (less than 0.00002% for *Wolbachia* and 0.00006% for *Rhabdochlamydia*) and the absence of a PCR product for these endosymbionts when performed on these samples.

Denaturing gel gradient electrophoresis (DGGE)

Six and five females originating from the WAL and DAM population respectively were included in the DGGE assay (Table 1). With the exception of one female from the WAL population, these individuals were also included in the pooled samples of the high-throughput sequencing assay. Besides these wild-caught females, two females of the *Wol*- and four females of the *Wol*+ matriline were also included in the DGGE analysis. Six DNA amplicons (gene library) from the *Wol*- and *Wol*+ matriline were included as a reference.

16S rRNA gene sequences were obtained using the universal primers F45 and R1242 [32]. Next, a nested PCR was performed with use of the primers F357 (5′-CCTACGGGAGGCAGCAG-3′) and R518 (5′-ATTACCGCGGCTGCTGG-3′) which amplify the highly variable V3 region of the 16S rRNA gene [40]. A GC-clamp was added to the forward primer to ensure clear DGGE separation [41,42]. PCR conditions were as follows: initial denaturation at 95°C for 1 min, followed by 30 cycles of denaturation at 95°C for 30 s, annealing (55°C for 45 s), extension (72°C, for 60 s) and a final extension at 72°C during 7 min. DGGE analysis was carried out on PCR products using a DCode Universal Mutation Detection System device (Bio-Rad) [as described in 40,43]. Electrophoresis was performed using 35%-70% denaturating gradient polyacrylamide gels at 70 V in 1x TAE buffer at 60°C for 16.5 hours. This allows discrimination of the different amplified 16S rRNA sequences based on nucleotide composition. Gels were placed in the staining solution SYBR gold (Molecular Probes, Invitrogen) for 30 min after which the gel was visualized and photographed with the Molecular Imager Gel Doc XR System. Selected bands were excised by inserting a pipette tip (1 μl) into the gel and incubated overnight at 4°C in TE buffer. A PCR with the primers F357 and R518 [32] was performed on the DNA solution and PCR amplicons were sequenced as described above. A BLAST search was performed to obtain the best match in the nucleotide collection database.

### PCR screening and phylogeny of endosymbionts

As the high throughput sequencing revealed the presence of *Rhabdochlamydia*, the prevalence of this endosymbiont was further investigated using PCR screening of 19 females and 38 males from the WAL population and 14 females and 14 males from the DAM population. We also included *Arsenophonus* in the PCR screening as this is the only reproductive endosymbiont that was not included in the previous study [28] (n = 14 and n = 27 for Damvallei and Walenbos females respectively). Based on the 16S rRNA sequence obtained from the cloning assay, we designed the primers Rhab16S-F1 5’-CGA GCC TGG GTA AGG TTC TTC-3’and Rhab16S-R1 5’-CTA TCA AAG TGG GGG CCC TTG-3’ to selectively amplify a part of the 16S rRNA gene of *Rhabdochlamydia*. PCR conditions were as follows: initial denaturation at 95°C for 2 min, followed by 35 cycles of denaturation at 94°C for 30 s, annealing at 52°C for 30 s, extension at 72°C for 90 s and a final extension at 72°C during 5 min.

We also tested for the presence of *Arsenophonus* using the primers and protocols reported in [24]. Electrophoresis was performed on a 1,5% agarose gel. Gels were stained in a solution of GELRED for approximately 15 min and bands were visualized by UV-fluorescence. Ten PCR products were sequenced using BigDye v.1.1 Terminator Sequencing mix and run on an ABI 3130 automated sequencer to check for primer specificity. To investigate the presence of multiple strains, sequences were aligned using the Muscle algorithm [44] implemented in MEGA5 [45] and checked for the presence of single nucleotide polymorphisms.

Phylogenetic position of the *Rhabdochlamydia* 16S gene sequence was compared to other *Chlamydiae* sequences [reported in 46]. A p-distance based Neighbour joining tree was constructed as implemented in MEGA 6 [47]. Bootstrap percentage support was calculated for the nodes by generating 10000 bootstrap values.

## Results

### Cloning of 16S rRNA

A total of 82 clones were isolated from the two different females of the *Wol-* matriline and consisted of two groups of identical sequences. Sequences of the largest group (77 sequences) showed a close match with *Rickettsia limoniae* [Genbank:KF720712] (Table 2). Sequences obtained from the five remaining clones showed a very high similarity with *Rhabdochlamydia porcellionis* [KF720713] (Table 2). Sequences of the 19 clones of the two females of the *Wol+* matriline could equally be grouped into two groups of identical sequences (Table 2). 13 appeared identical with our previously obtained 16S rRNA gene sequence of *Wolbachia* in *Oedothorax gibbosus* [GenBank:HQ286291], while sequences of the remaining six clones were identical with the *Rickettsia* sequence obtained from the *Wol-* matriline (Table 2).

**Table 2:** Top four BLAST hits against NCBI nucleotide collection and NCBI 16S rRNA databases for the three distinct sequences obtained from cloning of 16S rRNA from *Oedothorax gibbosus*.

N Clones			NCBI nucleotide collection				NCBI 16S ribosomal RNA			
Sequence	Wol-	Wol+	Species [Accession number]	Score	E-value	Ident	Species [Accession number]	Score	E-value	Ident
KF720713	5	0	*Candidatus* Rhabdochlamydia porcellionis [HF933203]	1452	0	0,98	*Parachlamydia acanthamoebae* [NR_026357]	917	0	0,87
			*Candidatus* Rhabdochlamydia porcellionis [AY223862]	1450	0	0,98	*Parachlamydia acanthamoebae* [NR_074972]	915	0	0,87
			*Candidatus* Rhabdochlamydia sp. [JF513056]	1399	0	0,97	*Simkania negevensis* [NR_074932]	887	0	0,86
			*Rhabdochlamydia crassificans* [AY928092]	1382	0	0,96	*Candidatus Protochlamydia amoebophila* [NR_074271]	878	0	0,88
KF720712	77	6	*Rickettsia limoniae* [AF322443]	1480	0	0,99	*Rickettsia slovaca* [NR_074474]	1323	0	0,96
			*Rickettsia limoniae* [AF322442]	1480	0	0,99	*Rickettsia slovaca* [NR_074462]	1323	0	0,96
			*Rickettsia* endosymbiont of *Macrolophus* sp. [HE583203]	1474	0	0,99	*Rickettsia australis* [NR_074496]	1317	0	0,96
			*Rickettsia* endosymbiont of *Asobara tabida* [FJ603467]	1474	0	0,99	*Rickettsia peacockii* [NR_074488]	1317	0	0,96
HQ286291	0	13	*Wolbachia* endosymbiont of *Tetragnatha montana* [EU333940]	1450	0	1	*Wolbachia* sp. wRi [NR_074437]	1439	0	0,99
			*Wolbachia* endosymbiont of *Oedemeronia lucidicollis* [GU236934]	1445	0	0,99	*Wolbachia* endosymbiont of *Brugia malayi* [NR_074571]	1387	0	0,98
			*Wolbachia* sp. Pin [GQ167635]	1445	0	0,99	*Wolbachia* endosymbiont of *Culex quinquefasciatus* [NR_074127]	1378	0	0,98
			*Wolbachia* sp. Psq [GQ167634]	1445	0	0,99	*Ehrlichia ruminatium* [NR_074155]	1038	0	0,9

### High-throughput sequencing of the 16SrRNA V3 and V4 region

**Fig 1:**
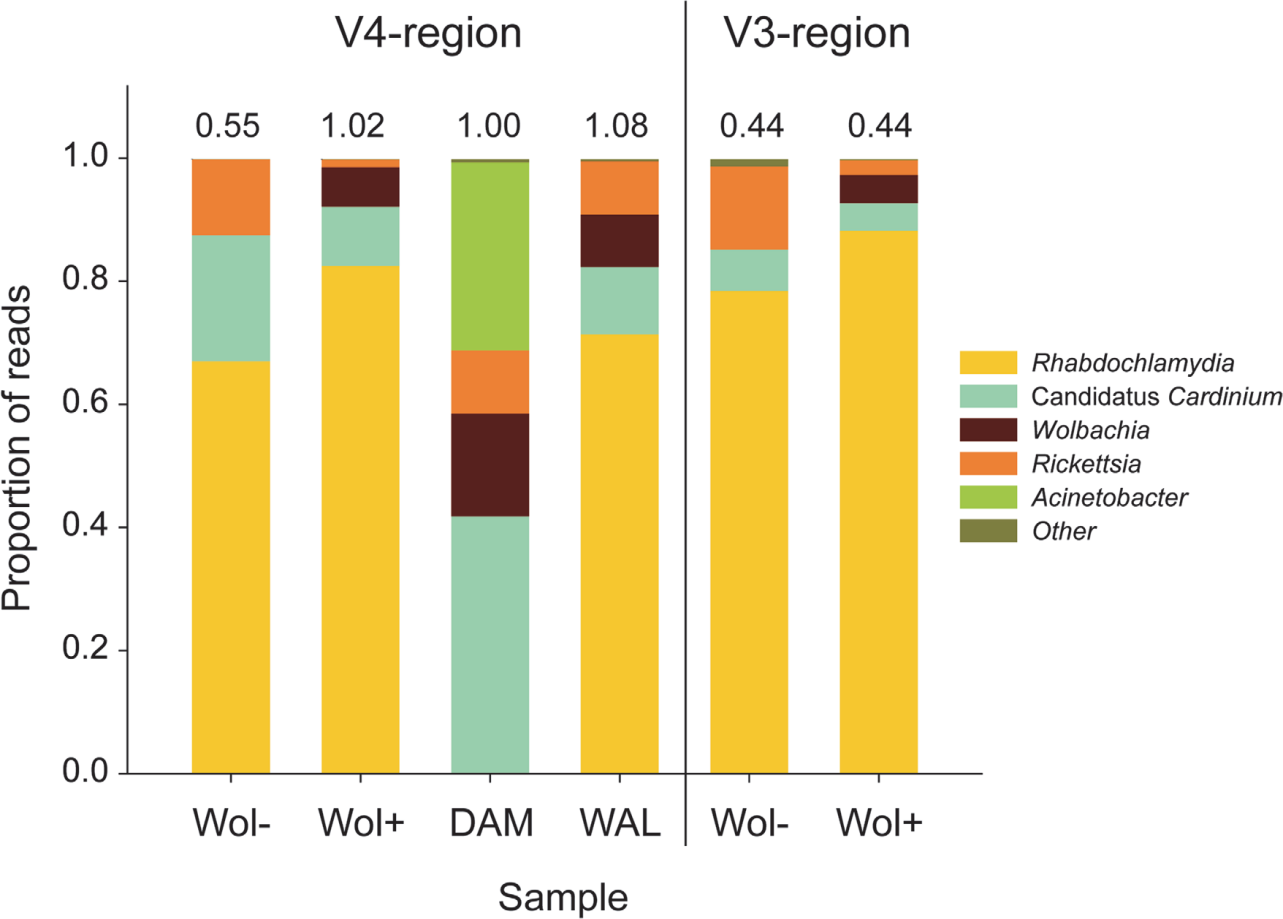
Proportion of paired 100bp Illumina reads from the 16S rRNA-V4 and 16S rRNA-V3 region assigned to the different bacterial taxa in the dwarf spider *Oedothorax gibbosus*. *Wol+* and *Wol*- are individual females from maternal lines infected with and without *Wolbachia* respectively. DAM and WAL represent samples consisting of ten pooled wild caught females from population Damvallei and Walenbos respectively. Numbers above bars represent the number of reads in millions.

Phylotype analysis revealed that sequences matched to 210 (V3) and 327 (V4) different bacterial taxa. The distribution of the sequences among these different taxa was highly uneven and, with the exception of the pooled DAM sample, > 99% of the sequences were assigned to four bacterial taxa only. RDP based taxonomic classification of the most abundant sequences within each phylotype showed that 107 (V3) and 74 (V4) of these sequences showed similarity scores >80%. For all samples, the most frequent sequence was classified as *Candidatus* Rhabdochlamydia porcellionis (Fig. 1) and its sequence was identical to the 16S rRNA gene sequence obtained from cloning analysis. The other three bacteria that showed a high number of reads were classified as *Wolbachia, Rickettsia* and *Candidatus* Cardinium hertigii (Fig. 1). Also for *Wolbachia* and *Rickettsia*, representative sequences were identical to the sequences obtained from cloning. Only for the pooled DAM sample, an additional bacterium was found that represented 30% of the reads of this sample and closely matched with *Acinetobacter*.

The remaining taxa that could be classified with relative high similarity scores were only found in very low frequencies and did not yield consistent results between the V3 and V4 region. For the V3 region, these were an unclassified Bacteriodetes (0.2%) and a *Ruminococcus* species (0.05%) and for the V4 region an unclassified member of the Enterobacteriaceae (0.09%) and an unclassified member of the order Saprospirales (0.017%). Despite the relatively low number of bacterial species associated with *O. gibbosus*, species composition was not similar between the two individuals and the two pooled population samples. For the two individual samples amplification of the V3 and V4 region produced consistent results (Fig. 1). As expected, high-throughput sequencing did not reveal any match with *Wolbachia* in the individual originating from the *Wol-* maternal line, confirming that *Wolbachia* is not the causative agent for the production of highly female biased clutches in this maternal line. This is in contrast with the *Wol+* matriline where in concordance with previous results, *Wolbachia* was positively identified. Both lines showed infection with *Rhabdochlamydia, Cardinium* and *Rickettsia*. However, the *Wol-* individual showed a much higher proportion of *Rickettsia* relative to *Cardinium* and *Rhabdochlamydia* compared to the *Wol+* line. The relative ratio of *Rickettsia*/*Rhabdochlamydia* and the ratio *Rickettsia*/*Cardinium* being 6.3 (V3) to 12.2 (V4) and 3.7 (V3) to 4.6 (V4) times higher in the *Wol-* compared to the *Wol+* individual. As this increase was consistently observed in both datasets, this likely reflects a higher *Rickettsia* infection rate in the *Wol-* line.

Bacterial species composition was furthermore not similar between the two pooled population samples. While *Rhabdochlamydia* appears to be the predominant bacterial species present in the WAL individuals, none of the sequences in the DAM population were assigned to this or a related bacterial species. Conversely, *Acinetobacter* appeared to be only present in the pooled DAM sample. *Cardinium, Wolbachia* and *Rickettsia* were found to infect both populations.

### Denaturing Gel Gradient Electrophoresis (DGGE)

**Fig 2:**
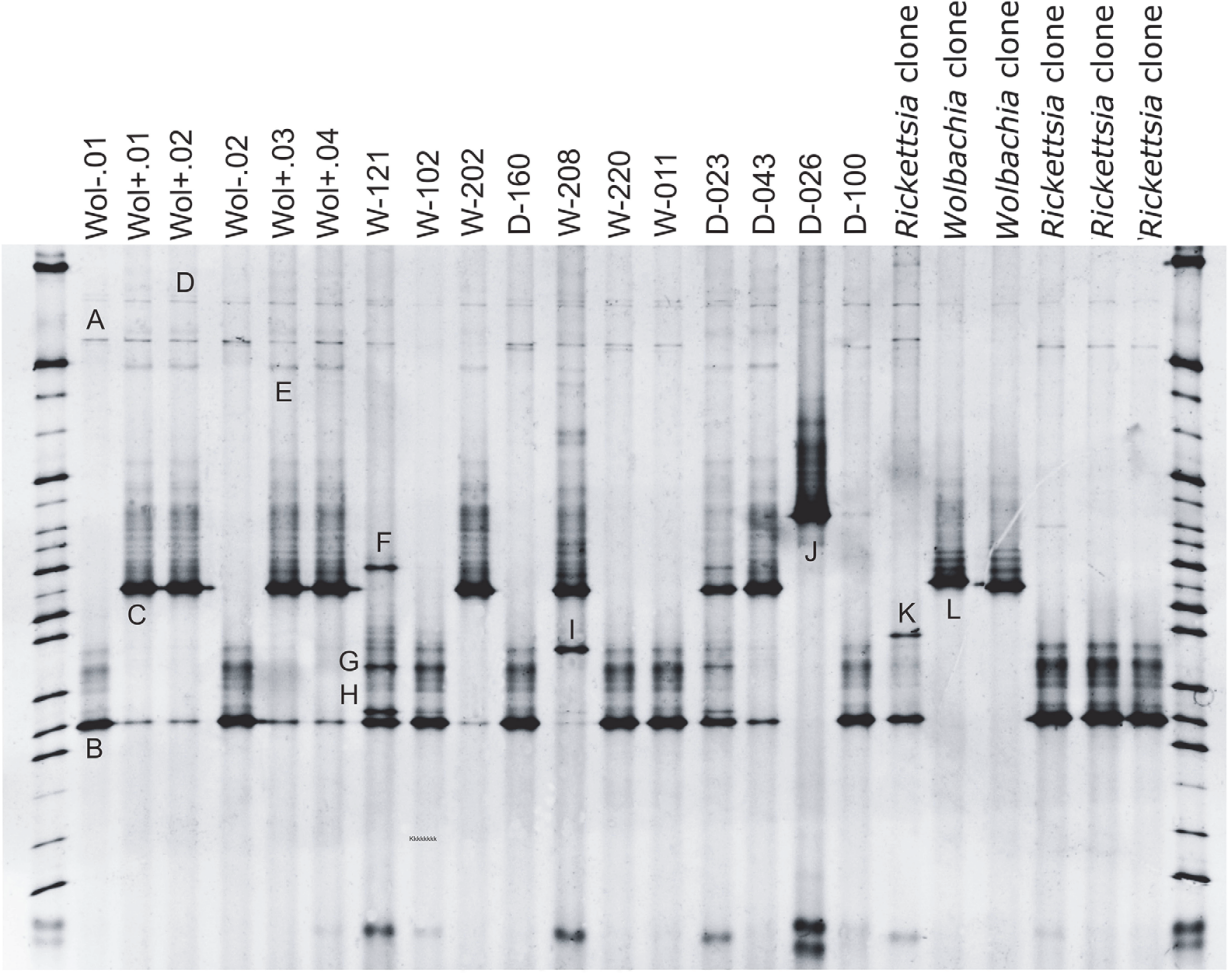
DGGE profiles for the 16S rRNA amplicons of females originating from the *Wol-* and *Wol+* matriline and females of the Damvallei (D) and Walenbos (W) population. Clones indicate the use of DNA amplicons resulting from the cloning study. Bands B and D: *Rickettsia* endosymbiont; C and L: *Wolbachia* endosymbiont; J: *Acinetobacter* sp.; sequences from other bands were ambiguous.

A total of 12 bands (A—L) were selected for sequencing (Fig. 2; Table 3) and revealed that these correspond to identical sequences of *Wolbachia* (C and L), *Rickettsia* (B and D) and *Acinetobacter* (J). Sequencing of bands A, E, F, G, H, I, and K were ambiguous. Several DNA amplicons from the cloning study were included in the DGGE as a reference. This resulted in one clear band in the DGGE profiles corresponding to *Rickettsia* and *Wolbachia* of the *Wol*- and *Wol*+ matrilines respectively.

**Table 3:** Taxonomic affiliation of the sequenced bands obtained by DGGE of Walenbos and Damvallei females.

Bands	NCBI Nucleotide collection closest match (accession number)	E-value (maximum identity)
C,L	*Wolbachia* endosymbiont of *Bemisia tabaci* (KF454764)	<1e-82 (100%)
B,D	*Rickettsia* endosymbiont of *Mermessus sp*. (KJ546647)	<9e-84 (100%)
J	*Acinetobacter sp*. (KJ009404)	<1e-97 (100%)

### PCR screening and phylogeny of endosymbionts

PCR screening revealed that *Rhabdochlamydia* detection was not restricted to the females used in the pooled sample for high-throughput sequencing, but that all females of the WAL population tested positive and 26% of the males (n = 19 and n = 38 for females and males respectively). Moreover, *Rhabdochlamydia* was not detected in the DAM population (n = 14 for both females and males). Primer specificity was subsequently confirmed by sequencing the obtained amplicons and yielded no interspecific variation.

PCR screening for *Arsenophonus* did not reveal any positives, indicating that this endosymbiont is absent in the two investigated populations (n = 14 and n = 27 for Damvallei and Walenbos females respectively).

**Fig 3:**
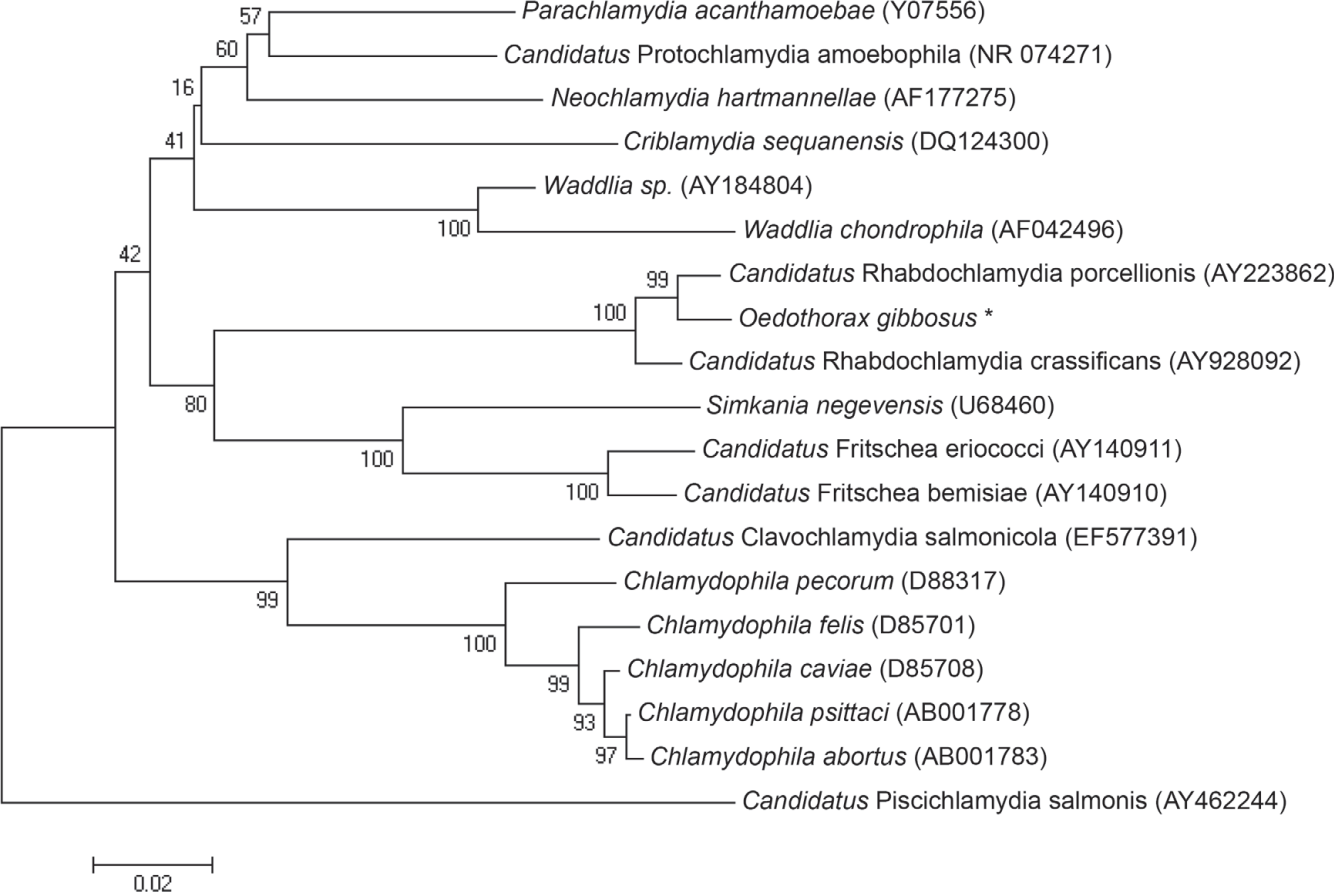
Phylogenetic position of the 16S rRNA gene sequence of *Rhabdochlamydia* of *Oedothorax gibbosus*. A p-distance based Neighbour Joining tree was constructed as implemented in MEGA 6 [47] on a subset of *Chlamydiae* sequences available at Genbank. Percentage bootstrap support was generated for the nodes. Accession numbers are given between brackets, *Rhabdochlamydia* found in *Oedothorax gibbosus* is marked with an asterisk.

The *Rhabdochlamydia* 16S rRNA gene sequences showed high similarity with existing *Rhabdochlamydia* 16S rRNA gene sequences and were most closely related to *Candidatus* Rhabdochlamydia porcellionis (Genbank accession number: AY223862), followed by *Candidatus* Rhabdochlamydia crassificans (Genbank accession number: AY928092) (Fig. 3).

## Discussion

Bacterial symbionts can have a vast diversity of relationships with their hosts and consequently have a high potential in affecting host ecology and evolution [1,3,48,49]. In *Oedothorax gibbosus*, where at least one endosymbiont (*Wolbachia*) causes a distorted sex ratio, we investigated the bacterial community in two different populations. This showed that these communities are dominated by four endosymbionts: *Rhabdochlamydia, Cardinium, Wolbachia* and *Rickettsia*. In all but one of the samples these four genera make up > 99% of the reads. Only for the DAM population, *Acinetobacter sp*. is found in approx. 30% of the reads with DGGE profiles showing that this is most likely due to detection of this bacterial species in a single individual. Screening of females showed that *Rhabdochlamydia* detection is limited to the WAL populations as all investigated females tested positive compared to none of the females in the DAM population.

Bacteria of the Chlamydiales are considered obligate intracellular parasites and are known to infect arthropods [50–52]. In scorpions, pathogenic effects are observed caused by a *Porochlamydia* infection in the hepatopancreas [53]. Although *Chlamydia*-like micro-organisms have been observed in spiders [22], this is the first report of *Rhabdochlamydia* as the genus has only recently been described [54,55]. *Candidatus* Rhabdochlamydia crassificans [55] infects cockroaches and causes pathogenic abdominal swelling of the host. The effects of *Candidatus* Rhabdochlamydia porcellionis [54] in the terrestrial isopod *Porcellio scaber* are currently not known. To date, no effects on host reproduction are documented. However, in the cockroach *Blatta orientalis, Rhabdochlamydia* was isolated from both fat body and ovary tissue [55]. The association with reproductive tissue could be a first indicator of potential reproductive effects. This is further suggested from our data by the difference in infection frequency between the sexes observed in the WAL population where the prevalence appeared to be much higher in females compared to males.

The NGS analysis of the pooled sample of the DAM population and a single DGGE profile revealed the presence of *Acinetobacter sp*. This bacterial genus is commonly found in environmental samples [56] and has been found to infect a variety of arthropods both internally as on the cuticle [57–61]. As whole spiders were used it is currently not known if *Acinetobacter* is located externally or internally.

Beside the *Rhabdochlamydia* detection pattern, both populations are similar in the frequencies of the other endosymbionts. *Rickettsia* and *Cardinium* are fixed in the two, both in males and females, while *Wolbachia* is found in approximately half of the male and female individuals [28, and this study] while *Arsenophonus* is absent in both populations. The cloning assay, DGGE profiles and high-throughput sequencing each support the absence of *Wolbachia* in the *Wol-* matriline. Still, this matriline is characterised by a highly distorted female biased sex ratio (5% males), suggesting that a second sex ratio distorting element, besides *Wolbachia*, is present in this species. No bacterial taxon was uniquely found in the *Wol-* matriline rendering it unlikely that a yet unidentified bacterial endosymbiont is responsible for the sex ratio trait. Given that this sex ratio trait is mainly inherited through daughters (Vanthournout & Hendrickx, *unpublished*), it is most likely that one of the endosymbionts (i.e. *Rhabdochlamydia, Rickettsia* or *Cardinium*) are responsible for the strong female bias. Of these, a higher ratio of *Rickettsia* was found in the *Wol-* matriline compared to the *Wol+* matriline. This increase could reflect the absence of competition with *Wolbachia* and explain the manifestation of a sex ratio distortion if the penetrance of the endosymbiont is density dependent [62,63]. Alternatively, the presence of different strains of these endosymbionts in the *Wol-* matriline could account for a female bias in the absence of *Wolbachia*. As we targeted the conservative 16S rRNA gene, subtle genetic differences between strains that cause differences in the ability to manipulate sex ratios could be overlooked. Future investigations should explore the densities of the different endosymbionts in this matriline through real-time PCR and verify the presence of different strains through multi locus sequencing and 16S rRNA libraries. At last, it still remains possible that a non-bacterial species is responsible for this sex-ratio bias. Microsporidia, for example, show a high degree of vertical transmission and sex ratio manipulation of the host by these endosymbionts has been frequently reported, in particular for crustaceans such as amphipods [64]. Although both high-throughput sequencing, the cloning assay and DGGE profiles demonstrate the absence of *Wolbachia* in the *Wol-* matriline, striking differences in their detection of the other endosymbionts were observed. Illumina sequencing and the use of endosymbiont specific primers indicate that both matrilines are infected with *Rickettsia, Cardinium* and *Rhabdochlamydia*. The cloning assay failed to detect *Cardinium* in both maternal lines and *Rhabdochlamydia* in the *Wol+* matriline. Similarly, DGGE profiles did not show infection with *Cardinium* and *Rhabdochlamydia* in any of the samples. This represents an underestimation of the bacterial diversity in the cloning assay and DGGE approach and illustrates the merit of high-throughput sequencing. One the one hand, our use of pooled samples demonstrates that also rare infections can be detected as was the case for *Acinetobacter*. However, this also illustrates that care should be taken with interpreting read frequencies of pooled samples as single infections may apparently result in a significant proportion of the reads (30%) from this species, suggesting a high prevalence of *Acinetobacter* in this population if not combined with individual approaches.

Notwithstanding these differences, all methods support the same conclusion: the bacterial community of this spider species appears to be almost completely restricted to endosymbiont bacteria. This contrasts with recent studies of arthropod bacterial communities where typically a large number of taxa is found of which the majority is not endosymbiotic. [7–9,65–68]. Given that whole specimens were used that did not receive any treatment for the removal of bacteria from the integument, this suggests that *Oedothorax gibbosus* is characterized by a unique bacterial community that consist almost solely of intracellular endosymbionts and an absence of gut microbiota. This is in line with the results of endosymbiont screening studies showing that a high number of spider species are infected [23–27]. However, in the medical studies, fangs and venom of spiders of the *Loxosceles* genus were surveyed for pathogenic members of *Clostridium* [13,14], while in *Tegenaria agrestis* [11] and in several species of human associated spiders [10] both the internal and external bacterial community was characterised. None of these studies detected an infection with endosymbiont bacteria. These results reflect most likely the limitations of the studies’ methodology as either a specific bacterium was targeted or a medium based rearing of bacteria was employed which excludes the possibility of finding non culturable endosymbiont bacteria.

It remains therefore most interesting to verify in multiple spider species to what extent their bacterial communities are dominated by endosymbiont bacteria.

## Conclusions

In order to obtain a complete overview of the bacterial community in a dwarf spider species a next generation sequencing approach in combination with a cloning assay and DGGE profiles was used. We found that individuals can be co-infected with up to four endosymbiont bacteria, i.e. *Wolbachia, Rickettsia, Cardinium* and *Rhabdochlamydia*. In one sample a high abundance of *Acinetobacter* was detected. Remarkably, virtually no other bacterial species were detected in this spider species, which suggest that its bacterial community is dominated by endosymbiont bacteria. Given the high prevalence of endosymbiont infection in spiders, future screening studies need to determine the dominance of endosymbiont bacteria in spider bacterial assemblages.

## Supporting Information

S1 Information Python script Concatenate Non-overlaps. (DOCX)

S2 Information List of 16S rRNA V3 region OTU’s and number of sequences associated with each OTU in two individuals (*Wol-* and *Wol+*) of the dwarf spider *Oedothorax gibbosus*. OTU’s were determined by phylotype analysis wherein sequences were clustered according to their match with sequences in the SILVA database (as implemented in MOTHUR v 1.29.0) using the Greengenes taxonomic classification.
(PDF)

S3 Information List of 16S rRNA V3 region OTU’s and number of sequences associated with each OTU in two individuals (*Wol-* and *Wol+*) of the dwarf spider *Oedothorax gibbosus*. Taxonomic classification was based on comparison of the most abundant sequence from each cluster against both the type and non-type and cultured and uncultured strain bacterial database using the Ribosomal Database Project (RDP; http://rdp.cme.msu.edu/, accessed 02/07/2013)(Cole et al. 2009). Taxonomy is based on the NCBI nomenclature. OTU’s were determined by phylotype analysis wherein sequences were clustered according to their match with sequences in the SILVA database (as implemented in MOTHUR v 1.29.0) using the Greengenes taxonomic classification.
(PDF)

S4 Information List of 16S rRNA V4 region OTU’s and number of sequences associated with each OTU in two individuals (*Wol-* and *Wol+*) and two pooled population samples (DAM and WAL) of the dwarf spider *Oedothorax gibbosus*. OTU’s were determined by phylotype analysis wherein sequences were clustered according to their match with sequences in the SILVA database (as implemented in MOTHUR v 1.29.0) using the Greengenes taxonomic classification.
(PDF)

S5 Information List of 16S rRNA V4 region OTU’s and number of sequences associated with each OTU in two individuals (*Wol-* and *Wol+*) and two pooled population samples (DAM and WAL) of the dwarf spider *Oedothorax gibbosus*. Taxonomic classification was based on comparison of the most abundant sequence from each cluster against both the type and non-type and cultured and uncultured strain bacterial database using the Ribosomal Database Project (RDP; http://rdp.cme.msu.edu/, accessed 02/07/2013)(Cole et al. 2009). Taxonomy is based on the NCBI nomenclature. OTU’s were determined by phylotype analysis wherein sequences were clustered according to their match with sequences in the SILVA database (as implemented in MOTHUR v 1.29.0) using the Greengenes taxonomic classification.
(PDF)

S6 Information Detailed analysis strategy of bacterial 16S-V4 and 16S-V3 Illumina HiSeq PE 100bp reads in *Oedothorax gibbosus*. (DOCX)
