# Supplementary material for: Endosymbiont Dominated Bacterial Communities in a Dwarf Spider
Source: PLoS One. 2015 Feb 23;10(2):e0117297. doi: 10.1371/journal.pone.0117297 (PMC4338242; doi:10.1371/journal.pone.0117297)
Supplement: S1 Information — (DOCX) [file pone.0117297.s001.docx]

'''
Created on 28 Jul 2012
@author: fhendrickx
'''
print('ConcatenateNonoverlaps 0.0.1\nProgram to join forward and reverse read from fastq files\nn ambigous base pairs are inserted between both reads\n')
import re

forward_reads=raw_input("Filename R1 reads: ")
reverse_reads=raw_input("Filename R2 reads (reverse complement!): ")
results=raw_input("Output filename: ")
n=input("Number of ambigous base pairs to insert: ")
n=int(n)

p=re.compile('\@HWI')
q=re.compile('\+$')
s=re.compile('^[ACGTN][ACTGN]*[ACTGN]$')

file1=open(forward_reads)
file2=open(reverse_reads)
file3=open(results,'w')

nlines=0
for lines in file1:
 nlines+=1

nseqs=nlines/4
print 'Number of forward sequences=',nseqs

nlines2=0
for lines in file2:
 nlines2+=1

nseqs2=nlines2/4
print 'Number of reverse sequences=',nseqs2
print 'Concatenating forward and reverse reads. Please wait....'

amb_bases=n*'N'
amb_phred=n*'#'

file1.seek(0)
file2.seek(0)
for i in range(nlines):

string1=str(file1.readline())
 string2=str(file2.readline())
 if p.match(string1):
 file3.write(string1)
 elif q.match(string1):
 file3.write(string1)
 elif s.match(string1):
 string3=string1[:-1]+amb_bases+string2
 file3.write(string3)
 else:
 string4=string1[:-1]+amb_phred+string2
 file3.write(string4)

file1.close()
file2.close()
file3.close()

print 'Results written to file',"'",results,"'"
raw_input("Press <enter> to quit")
