# Supplementary material for: Endosymbiont Dominated Bacterial Communities in a Dwarf Spider
Source: PLoS One. 2015 Feb 23;10(2):e0117297. doi: 10.1371/journal.pone.0117297 (PMC4338242; doi:10.1371/journal.pone.0117297)
Supplement: S2 Information — OTU’s were determined by phylotype analysis wherein sequences were clustered according to their match with sequences in the SILVA database (as implemented in MOTHUR v 1.29.0) using the Greengenes taxonomic classification. (PDF) [file pone.0117297.s002.pdf]

OTU	Number of reads		Greengenes taxonomic classification
	Wol-	Wol+	
Otu001	346830	392414	Bacteria(100);Chlamydiae(100);Chlamydophila(100);Unclassified(100);unclassified(100);unclassified(100);unclassified(100);unclassified(100);unclassified(100);unclassified(100);
Otu002	29706	19864	Bacteria(100);Bacteroidetes(100);Cardiniaceae(100);Candidatus_Cardinium(100);unclassified(100);unclassified(100);unclassified(100);unclassified(100);unclassified(100);unclassified(100);
Otu003	126	0	Bacteria(100);Firmicutes(100);Clostridia(100);Clostridiales(100);butyrate-producing_bacterium_A2-207(100);Unclassified(100);unclassified(100);unclassified(100);unclassified(100);unclassified(100);
Otu004	476	0	Bacteria(100);Firmicutes(100);Clostridia(100);Clostridiales(100);Ruminococcus(100);Unclassified(100);unclassified(100);unclassified(100);unclassified(100);unclassified(100);
Otu005	2	0	Bacteria(100);Firmicutes(100);Clostridia(100);Clostridiales(100);Anaerofilum(100);Unclassified(100);unclassified(100);unclassified(100);unclassified(100);unclassified(100);
Otu006	339	2	Bacteria(100);Firmicutes(100);Clostridia(100);Clostridiales(100);Clostridium_nexile(100);Clostridium_fusiformis(100);Unclassified(100);unclassified(100);unclassified(100);unclassified(100);
Otu007	59847	10718	Bacteria(100);Proteobacteria(100);Alphaproteobacteria(100);Consistiales(100);Rickettsiales(100);spotted_fever_group(100);unclassified(100);unclassified(100);unclassified(100);unclassified(100);
Otu008	0	20437	Bacteria(100);Proteobacteria(100);Alphaproteobacteria(100);Consistiales(100);Rickettsiales(100);Wolbachia(100);Unclassified(100);unclassified(100);unclassified(100);unclassified(100);
Otu009	1785	0	Bacteria(100);Bacteroidetes(100);Bacteroidales(100);Bacteroidaceae(100);Unclassified(100);unclassified(100);unclassified(100);unclassified(100);unclassified(100);unclassified(100);
Otu010	300	152	Bacteria(100);Proteobacteria(100);Alphaproteobacteria(100);Consistiales(100);Unclassified(100);unclassified(100);unclassified(100);unclassified(100);unclassified(100);unclassified(100);
Otu011	94	0	Bacteria(100);Firmicutes(100);Clostridia(100);Clostridiales(100);AN1045(100);unclassified(100);unclassified(100);unclassified(100);unclassified(100);unclassified(100);
Otu012	115	152	Bacteria(100);Planctomycetes(100);Kueneniaceae(100);Brocadia(100);Candidatus_Anammoxoglobus(100);Unclassified(100);unclassified(100);unclassified(100);unclassified(100);unclassified(100);
Otu013	0	1	Bacteria(100);Bacteroidetes(100);Bacteroidales(100);BSV13(100);unclassified(100);unclassified(100);unclassified(100);unclassified(100);unclassified(100);unclassified(100);
Otu014	2	0	Bacteria(100);Firmicutes(100);Clostridia(100);Clostridiales(100);Clostridiaceae(100);Clostridium(100);Clostridium_butyricum_T(100);unclassified(100);unclassified(100);unclassified(100);
Otu015	89	0	Bacteria(100);Proteobacteria(100);Desulfovibrionales(100);Desulfovibrionaceae(100);Desulfovibrio(100);Unclassified(100);unclassified(100);unclassified(100);unclassified(100);unclassified(100);
Otu016	2	2	Bacteria(100);Firmicutes(100);Desulfotomaculum(100);Desulfotomaculum_nigrificans(100);Desulfotomaculum_aeronauticum(100);unclassified(100);unclassified(100);unclassified(100);unclassified(100);unclassified(100);
Otu017	65	7	Bacteria(100);Proteobacteria(100);Epsilonproteobacteria(100);Arcobacteraceae(100);Unclassified(100);unclassified(100);unclassified(100);unclassified(100);unclassified(100);unclassified(100);
Otu018	78	92	Bacteria(100);Chlamydiae(100);Simkaniaceae(100);unclassified(100);unclassified(100);unclassified(100);unclassified(100);unclassified(100);unclassified(100);unclassified(100);
Otu019	97	4	Bacteria(100);Proteobacteria(100);Gammaproteobacteria(100);Xanthomonadales(100);Unclassified(100);unclassified(100);unclassified(100);unclassified(100);unclassified(100);unclassified(100);
Otu020	300	5	Bacteria(100);Proteobacteria(100);Gammaproteobacteria(100);Pseudomonadaceae(100);Unclassified(100);unclassified(100);unclassified(100);unclassified(100);unclassified(100);unclassified(100);
Otu021	96	1	Bacteria(100);Firmicutes(100);Clostridia(100);Clostridiales(100);Ruminococcus(100);PL-38B10(100);unclassified(100);unclassified(100);unclassified(100);unclassified(100);
Otu022	34	26	Bacteria(100);Planctomycetes(100);Kueneniaceae(100);Unclassified(100);unclassified(100);unclassified(100);unclassified(100);unclassified(100);unclassified(100);unclassified(100);
Otu023	58	254	Bacteria(100);Proteobacteria(100);Gammaproteobacteria(100);Enterobacteriales_Enterobacteriaceae(100);Unclassified(100);unclassified(100);unclassified(100);unclassified(100);unclassified(100);unclassified(100);
Otu024	164	1	Bacteria(100);Bacteroidetes(100);Flavobacteriales(100);Flavobacteriaceae(100);Unclassified(100);unclassified(100);unclassified(100);unclassified(100);unclassified(100);unclassified(100);
Otu025	5	3	Bacteria(100);Proteobacteria(100);Alphaproteobacteria(100);Sphingomonadales(100);Sphingomonas_asaccharolytica(100);Sphingomonas_koreensis(100);unclassified(100);unclassified(100);unclassified(100);unclassified(100);
Otu026	153	0	Bacteria(100);Firmicutes(100);Clostridia(100);Peptostreptococcaceae(100);Mogibacterium(100);Unclassified(100);unclassified(100);unclassified(100);unclassified(100);unclassified(100);
Otu027	8	39	Bacteria(100);Proteobacteria(100);Alphaproteobacteria(100);Consistiales(100);Rickettsiales(100);Anaplasma(100);Unclassified(100);unclassified(100);unclassified(100);unclassified(100);
Otu028	4	21	Bacteria(100);Proteobacteria(100);Alphaproteobacteria(100);Rhodobacterales(100);Rhodobacter(100);Unclassified(100);unclassified(100);unclassified(100);unclassified(100);unclassified(100);
Otu029	1	1	Bacteria(100);Cyanobacteria(100);PRD01a012B(100);unclassified(100);unclassified(100);unclassified(100);unclassified(100);unclassified(100);unclassified(100);unclassified(100);
Otu030	55	81	Bacteria(100);OP3(100);BD4-9(100);unclassified(100);unclassified(100);unclassified(100);unclassified(100);unclassified(100);unclassified(100);unclassified(100);
Otu031	30	0	Bacteria(100);Firmicutes(100);Clostridia(100);Clostridiales(100);Acetivibrio(100);Clostridium_cellulolyticum(100);unclassified(100);unclassified(100);unclassified(100);unclassified(100);
Otu032	29	0	Bacteria(100);Proteobacteria(100);Gammaproteobacteria(100);Xanthomonadales(100);Stenotrophomonas(100);unclassified(100);unclassified(100);unclassified(100);unclassified(100);unclassified(100);
Otu033	10	6	Bacteria(100);Firmicutes(100);Clostridia(100);Clostridiales(100);Clostridium_viride(100);UC9-83(100);Unclassified(100);unclassified(100);unclassified(100);unclassified(100);
Otu034	3	6	Bacteria(100);Planctomycetes(100);Planctomycetacia(100);Pirellulales(100);unclassified(100);unclassified(100);unclassified(100);unclassified(100);unclassified(100);unclassified(100);
Otu035	12	0	Bacteria(100);Actinobacteria(100);Actinobacteridae(100);Unclassified(100);unclassified(100);unclassified(100);unclassified(100);unclassified(100);unclassified(100);unclassified(100);
Otu036	245	0	Bacteria(100);Bacteroidetes(100);Bacteroidales(100);Dysgonomonaceae(100);unclassified(100);unclassified(100);unclassified(100);unclassified(100);unclassified(100);unclassified(100);
Otu037	0	1	Bacteria(100);Acidobacteria(100);Acidobacteria-2(100);Unclassified(100);unclassified(100);unclassified(100);unclassified(100);unclassified(100);unclassified(100);unclassified(100);
Otu038	0	3	Bacteria(100);Acidobacteria(100);Chloracidobacteria(100);Ellin6075(100);unclassified(100);unclassified(100);unclassified(100);unclassified(100);unclassified(100);unclassified(100);
Otu039	7	0	Bacteria(100);Firmicutes(100);Clostridia(100);Clostridiales(100);Clostridium_nexile(100);Unclassified(100);unclassified(100);unclassified(100);unclassified(100);unclassified(100);
Otu040	1	0	Bacteria(100);Proteobacteria(100);Gammaproteobacteria(100);Piscirickettsiaceae(100);Thiobacillus_baregensis(100);Thiovirga(100);unclassified(100);unclassified(100);unclassified(100);unclassified(100);
Otu041	0	8	Bacteria(100);Proteobacteria(100);Alphaproteobacteria(100);Acetobacterales(100);Roseomonas(100);unclassified(100);unclassified(100);unclassified(100);unclassified(100);unclassified(100);
Otu042	1	0	Bacteria(100);Chrysiogenetes(100);unclassified(100);unclassified(100);unclassified(100);unclassified(100);unclassified(100);unclassified(100);unclassified(100);unclassified(100);
Otu043	7	3	Bacteria(100);Actinobacteria(100);Actinobacteridae(100);Gordoniaceae(100);Unclassified(100);unclassified(100);unclassified(100);unclassified(100);unclassified(100);unclassified(100);
Otu044	151	133	Bacteria(100);Planctomycetes(100);Kueneniaceae(100);Scalindua(100);unclassified(100);unclassified(100);unclassified(100);unclassified(100);unclassified(100);unclassified(100);
Otu045	42	0	Bacteria(100);Firmicutes(100);Clostridia(100);Clostridiales(100);Clostridium_viride(100);Unclassified(100);unclassified(100);unclassified(100);unclassified(100);unclassified(100);
Otu046	11	0	Bacteria(100);Bacteroidetes(100);Bacteroidales(100);Bacteroidaceae(100);PL-26B4(100);unclassified(100);unclassified(100);unclassified(100);unclassified(100);unclassified(100);
Otu047	0	7	Bacteria(100);Actinobacteria(100);Actinobacteridae(100);Gordoniaceae(100);Corynebacteriaceae(100);Unclassified(100);unclassified(100);unclassified(100);unclassified(100);unclassified(100);
Otu048	10	0	Bacteria(100);Firmicutes(100);Clostridia(100);Clostridiales(100);Clostridiaceae(100);Clostridium_novyi(100);Clostridium_botulinum(100);Unclassified(100);unclassified(100);unclassified(100);
Otu049	0	1	Bacteria(100);Synergistetes(100);Dethiosulfovibrionales(100);Jonquetella(100);unclassified(100);unclassified(100);unclassified(100);unclassified(100);unclassified(100);unclassified(100);
Otu050	4	16	Bacteria(100);Actinobacteria(100);Actinobacteridae(100);Intrasporangiaceae(100);Intrasporangiaceae(100);unclassified(100);unclassified(100);unclassified(100);unclassified(100);unclassified(100);
Otu051	62	40	Bacteria(100);Proteobacteria(100);Gammaproteobacteria(100);Moraxellaceae(100);Moraxella__Psychrobacter(100);Unclassified(100);unclassified(100);unclassified(100);unclassified(100);unclassified(100);
Otu052	42	1	Bacteria(100);Firmicutes(100);Mollicutes(100);Clostridium_aff_innocuum_CM970(100);Eubacterium_cylindroides(100);Unclassified(100);unclassified(100);unclassified(100);unclassified(100);unclassified(100);
Otu053	74	0	Bacteria(100);Proteobacteria(100);Epsilonproteobacteria(100);Sulfurospirillaceae(100);Sulfurospirillum(100);Unclassified(100);unclassified(100);unclassified(100);unclassified(100);unclassified(100);
Otu054	8	0	Bacteria(100);Firmicutes(100);Clostridia(100);Clostridiales(100);Clostridium_aminovalericum(100);unclassified(100);unclassified(100);unclassified(100);unclassified(100);unclassified(100);
Otu055	7	0	Bacteria(100);Firmicutes(100);Mollicutes(100);Catenibacterium(100);Clostridium_ramosum(100);Unclassified(100);unclassified(100);unclassified(100);unclassified(100);unclassified(100);

OTU	Number of reads		Greengenes taxonomic classification
	Wol-	Wol+	
Otu056	33	43	Bacteria(100);Acidobacteria(100);S035(100);unclassified(100);unclassified(100);unclassified(100);unclassified(100);unclassified(100);unclassified(100);unclassified(100);
Otu057	8	16	Bacteria(100);Actinobacteria(100);Actinobacteridae(100);Propionibacterineae(100);Propionibacterium(100);Unclassified(100);unclassified(100);unclassified(100);unclassified(100);unclassified(100);
Otu058	0	2	Bacteria(100);Firmicutes(100);Clostridia(100);Peptostreptococcaceae(100);Peptoniphilus(100);Unclassified(100);unclassified(100);unclassified(100);unclassified(100);unclassified(100);
Otu059	47	0	Bacteria(100);Bacteroidetes(100);Bacteroidales(100);Parabacteroidaceae(100);mle1-2(100);unclassified(100);unclassified(100);unclassified(100);unclassified(100);unclassified(100);
Otu060	3	0	Bacteria(100);Bacteroidetes(100);Bacteroidales(100);Parabacteroidaceae(100);Parabacteroides(100);unclassified(100);unclassified(100);unclassified(100);unclassified(100);unclassified(100);
Otu061	2	1	Bacteria(100);Proteobacteria(100);Alphaproteobacteria(100);Ellin329(100);Unclassified(100);unclassified(100);unclassified(100);unclassified(100);unclassified(100);unclassified(100);
Otu062	4	0	Bacteria(100);Proteobacteria(100);Gammaproteobacteria(100);Moraxellaceae(100);Unclassified(100);unclassified(100);unclassified(100);unclassified(100);unclassified(100);unclassified(100);
Otu063	10	16	Bacteria(100);Verrucomicrobia(100);Verruco-5(100);RFP12(100);Unclassified(100);unclassified(100);unclassified(100);unclassified(100);unclassified(100);unclassified(100);
Otu064	7	2	Bacteria(100);Firmicutes(100);014B-C8(100);unclassified(100);unclassified(100);unclassified(100);unclassified(100);unclassified(100);unclassified(100);unclassified(100);
Otu065	1	0	Bacteria(100);Caldithrix_KSB1(100);Unclassified(100);unclassified(100);unclassified(100);unclassified(100);unclassified(100);unclassified(100);unclassified(100);unclassified(100);
Otu066	1	1	Bacteria(100);Proteobacteria(100);Gammaproteobacteria(100);Ferrimonadaceae(100);Deselenobacterium(100);unclassified(100);unclassified(100);unclassified(100);unclassified(100);unclassified(100);
Otu067	3	0	Bacteria(100);Firmicutes(100);Clostridia(100);Clostridiales(100);Ruminococcus(100);Ruminococcus_luti(100);butyrate-producing_bacterium_PH07BW09(100);HuCB12(100);UC9-24(100);MA10(100);
Otu068	6	0	Bacteria(100);Proteobacteria(100);Gammaproteobacteria(100);Aeromonadaceae(100);Aeromonas(100);unclassified(100);unclassified(100);unclassified(100);unclassified(100);unclassified(100);
Otu069	23	0	Bacteria(100);Firmicutes(100);Clostridia(100);Eubacteriaceae(100);Acetobacterium(100);Acetobacterium_woodii(100);unclassified(100);unclassified(100);unclassified(100);unclassified(100);
Otu070	21	22	Bacteria(100);Firmicutes(100);Clostridia(100);Clostridiales(100);LW58(100);unclassified(100);unclassified(100);unclassified(100);unclassified(100);unclassified(100);
Otu071	31	4	Bacteria(100);Firmicutes(100);Clostridia(100);Clostridiales(100);Unclassified(100);unclassified(100);unclassified(100);unclassified(100);unclassified(100);unclassified(100);
Otu072	13	11	Bacteria(100);Proteobacteria(100);Deltaproteobacteria(100);Myxococcales(100);OM27(100);CTD005-73B-02(100);ctg_CGOF163(100);unclassified(100);unclassified(100);unclassified(100);
Otu073	18	18	Bacteria(100);Firmicutes(100);Bacilli(100);Staphylococcaceae(100);Unclassified(100);unclassified(100);unclassified(100);unclassified(100);unclassified(100);unclassified(100);
Otu074	9	19	Bacteria(100);Actinobacteria(100);Actinobacteridae(100);Ellin5022(100);unclassified(100);unclassified(100);unclassified(100);unclassified(100);unclassified(100);unclassified(100);
Otu075	30	0	Bacteria(100);Firmicutes(100);Clostridia(100);Clostridiales(100);Clostridium_neopropionicum(100);unclassified(100);unclassified(100);unclassified(100);unclassified(100);unclassified(100);
Otu076	1	0	Bacteria(100);Cyanobacteria(100);Chloroplasts(100);Unclassified(100);unclassified(100);unclassified(100);unclassified(100);unclassified(100);unclassified(100);unclassified(100);
Otu077	42	3	Bacteria(100);Bacteroidetes(100);Flavobacteriales(100);Flavobacteriaceae(100);Chryseobacterium(100);unclassified(100);unclassified(100);unclassified(100);unclassified(100);unclassified(100);
Otu078	9	1	Bacteria(100);Proteobacteria(100);Gammaproteobacteria(100);Oceanimonaceae(100);Oceanimonas(100);unclassified(100);unclassified(100);unclassified(100);unclassified(100);unclassified(100);
Otu079	5	4	Bacteria(100);Firmicutes(100);Clostridia(100);Clostridiales(100);Clostridiaceae(100);Clostridium_pasteurianum(100);unclassified(100);unclassified(100);unclassified(100);unclassified(100);
Otu080	9	2	Bacteria(100);Cyanobacteria(100);Chloroplasts(100);vectors(100);Unclassified(100);unclassified(100);unclassified(100);unclassified(100);unclassified(100);unclassified(100);
Otu081	0	26	Bacteria(100);Proteobacteria(100);Alphaproteobacteria(100);Consistiales(100);Rickettsiales(100);Wolbachia(100);Wolbachia_endosymbiont_of_Onchocerca_ochengi(100);unclassified(100);unclassified(100);unclassified(100);
Otu082	13	2	Bacteria(100);Bacteroidetes(100);Bacteroidales(100);Bacteroidaceae(100);Prevotellaceae(100);Unclassified(100);unclassified(100);unclassified(100);unclassified(100);unclassified(100);
Otu083	9	1	Bacteria(100);Actinobacteria(100);Actinobacteridae(100);Micromonosporaceae(100);unclassified(100);unclassified(100);unclassified(100);unclassified(100);unclassified(100);unclassified(100);
Otu084	4	0	Bacteria(100);Bacteroidetes(100);Flavobacteriales(100);Cytophaga(100);Chl112(100);unclassified(100);unclassified(100);unclassified(100);unclassified(100);unclassified(100);
Otu085	2	5	Bacteria(100);Firmicutes(100);Bacilli(100);Lactobacillales(100);Leuconostoc(100);Oenococcus(100);unclassified(100);unclassified(100);unclassified(100);unclassified(100);
Otu086	1	3	Bacteria(100);Planctomycetes(100);Planctomycetacia(100);Planctomycetales(100);Planctomycetales_bacterium_MPL7(100);unclassified(100);unclassified(100);unclassified(100);unclassified(100);unclassified(100);
Otu087	0	4	Bacteria(100);Proteobacteria(100);Deltaproteobacteria(100);Bacteriovorax(100);Bacteriovorax_marinus(100);unclassified(100);unclassified(100);unclassified(100);unclassified(100);unclassified(100);
Otu088	13	10	Bacteria(100);TM7(100);TM7-3(100);CW040(100);unclassified(100);unclassified(100);unclassified(100);unclassified(100);unclassified(100);unclassified(100);
Otu089	6	2	Bacteria(100);Bacteroidetes(100);Saprospirales(100);cilia-associated_respiratory_bacterium_R3(100);unclassified(100);unclassified(100);unclassified(100);unclassified(100);unclassified(100);unclassified(100);
Otu090	6	12	Bacteria(100);Proteobacteria(100);Alphaproteobacteria(100);Rhodobacterales(100);filamentous_bacteria_GR1(100);unclassified(100);unclassified(100);unclassified(100);unclassified(100);unclassified(100);
Otu091	10	12	Bacteria(100);Spirochaetes(100);Spirochaetales(100);Borreliaceae(100);Unclassified(100);unclassified(100);unclassified(100);unclassified(100);unclassified(100);unclassified(100);
Otu092	4	2	Bacteria(100);AC1(100);unclassified(100);unclassified(100);unclassified(100);unclassified(100);unclassified(100);unclassified(100);unclassified(100);unclassified(100);
Otu093	7	8	Bacteria(100);Proteobacteria(100);Gammaproteobacteria(100);Enterobacteriales_Enterobacteriaceae(100);Baumannia(100);unclassified(100);unclassified(100);unclassified(100);unclassified(100);unclassified(100);
Otu094	8	4	Bacteria(100);Synergistetes(100);Synergistales(100);Candidatus_Tammella(100);unclassified(100);unclassified(100);unclassified(100);unclassified(100);unclassified(100);unclassified(100);
Otu095	2	0	Bacteria(100);Thermotogae(100);MS9(100);unclassified(100);unclassified(100);unclassified(100);unclassified(100);unclassified(100);unclassified(100);unclassified(100);
Otu096	1	0	Bacteria(100);Firmicutes(100);Clostridia(100);Clostridiales(100);p-3487-9F3(100);unclassified(100);unclassified(100);unclassified(100);unclassified(100);unclassified(100);
Otu097	1	0	Bacteria(100);Proteobacteria(100);Gammaproteobacteria(100);Betaproteobacteria(100);Rhodocyclales(100);beta_proteobacterium_F06002(100);IRD18C09(100);unclassified(100);unclassified(100);unclassified(100);
Otu098	1	1	Bacteria(100);Acidobacteria(100);BPC015(100);unclassified(100);unclassified(100);unclassified(100);unclassified(100);unclassified(100);unclassified(100);unclassified(100);
Otu099	1	1	Bacteria(100);Planctomycetes(100);Planctomycetacia(100);DEL17(100);agg8(100);Unclassified(100);unclassified(100);unclassified(100);unclassified(100);unclassified(100);
Otu100	1	3	Bacteria(100);Firmicutes(100);Bacilli(100);Lactobacillales(100);Lactobacillaceae(100);Lactobacillus_perolens(100);unclassified(100);unclassified(100);unclassified(100);unclassified(100);
Otu101	3	2	Bacteria(100);ABY1_OD1(100);FW129(100);KNA6-NB29(100);unclassified(100);unclassified(100);unclassified(100);unclassified(100);unclassified(100);unclassified(100);
Otu102	1	0	Bacteria(100);Proteobacteria(100);Desulfurellales(100);Unclassified(100);unclassified(100);unclassified(100);unclassified(100);unclassified(100);unclassified(100);unclassified(100);
Otu103	0	3	Bacteria(100);Chloroflexi(100);Dehalococcoidetes(100);GIF9(100);MB-A2-101(100);Napoli-2B-07__BC07-2B-07(100);unclassified(100);unclassified(100);unclassified(100);unclassified(100);
Otu104	1	0	Bacteria(100);Firmicutes(100);Bacilli(100);Lactobacillales(100);Leuconostoc(100);Weissella(100);Weissella_kandleri(100);unclassified(100);unclassified(100);unclassified(100);
Otu105	1	0	Bacteria(100);Proteobacteria(100);Gammaproteobacteria(100);AL-2A(100);unclassified(100);unclassified(100);unclassified(100);unclassified(100);unclassified(100);unclassified(100);
Otu106	7	0	Bacteria(100);Proteobacteria(100);Gammaproteobacteria(100);Pseudomonadaceae(100);BRC56(100);unclassified(100);unclassified(100);unclassified(100);unclassified(100);unclassified(100);
Otu107	1	1	Bacteria(100);Chloroflexi(100);Chloroflexi(100);Chloroflexales(100);Chloroflexaceae(100);unclassified(100);unclassified(100);unclassified(100);unclassified(100);unclassified(100);
Otu108	1	1	Bacteria(100);Firmicutes(100);Clostridia(100);Clostridiales(100);B190(100);unclassified(100);unclassified(100);unclassified(100);unclassified(100);unclassified(100);
Otu109	1	0	Bacteria(100);Nitrospirae(100);Leptospirillaceae(100);Leptospirillum_gp_III(100);Leptospirillum_ferrodiazotrophum(100);BA29(100);RCP1-70(100);unclassified(100);unclassified(100);unclassified(100);
Otu110	0	1	Bacteria(100);Verrucomicrobia(100);LD19(100);unclassified(100);unclassified(100);unclassified(100);unclassified(100);unclassified(100);unclassified(100);unclassified(100);

OTU	Number of reads		Greengenes taxonomic classification
	Wol-	Wol+	
Otu111	7	0	Bacteria(100);Proteobacteria(100);Alphaproteobacteria(100);PB21(100);unclassified(100);unclassified(100);unclassified(100);unclassified(100);unclassified(100);unclassified(100);
Otu112	1	2	Bacteria(100);Cyanobacteria(100);Chloroplasts(100);Dinophysis(100);unclassified(100);unclassified(100);unclassified(100);unclassified(100);unclassified(100);unclassified(100);
Otu113	2	0	Bacteria(100);Bacteroidetes(100);Bacteroidales(100);vadinBC27(100);unclassified(100);unclassified(100);unclassified(100);unclassified(100);unclassified(100);unclassified(100);
Otu114	1	1	Bacteria(100);Firmicutes(100);Clostridia(100);Clostridiales(100);Ruminococcus(100);p-1082-a5(100);Unclassified(100);unclassified(100);unclassified(100);unclassified(100);
Otu115	8	0	Bacteria(100);Proteobacteria(100);Gammaproteobacteria(100);Enterobacteriales_Enterobacteriaceae(100);Yersinia(100);unclassified(100);unclassified(100);unclassified(100);unclassified(100);unclassified(100);
Otu116	5	0	Bacteria(100);Firmicutes(100);Clostridia(100);Clostridiales(100);Johnsonella(100);Eubacterium_cf_saburreum_oral_strain_C27KA(100);Unclassified(100);unclassified(100);unclassified(100);unclassified(100);
Otu117	2	1	Bacteria(100);TM7(100);TM7-1(100);K20-27(100);unclassified(100);unclassified(100);unclassified(100);unclassified(100);unclassified(100);unclassified(100);
Otu118	1	0	Bacteria(100);Bacteroidetes(100);Bacteroidales(100);Porphyromonadaceae(100);Porphyromonas_canis(100);Unclassified(100);unclassified(100);unclassified(100);unclassified(100);unclassified(100);
Otu119	1	0	Bacteria(100);ABY1_OD1(100);FW129(100);Unclassified(100);unclassified(100);unclassified(100);unclassified(100);unclassified(100);unclassified(100);unclassified(100);
Otu120	1	1	Bacteria(100);Firmicutes(100);Clostridia(100);Peptostreptococcaceae(100);Frigovirgula_patagoniensis(100);Clostridium_sticklandii(100);unclassified(100);unclassified(100);unclassified(100);unclassified(100);
Otu121	4	1	Bacteria(100);Actinobacteria(100);Actinobacteridae(100);Bifidobacteriaceae(100);Gardnerella(100);unclassified(100);unclassified(100);unclassified(100);unclassified(100);unclassified(100);
Otu122	2	2	Bacteria(100);ZB3(100);Unclassified(100);unclassified(100);unclassified(100);unclassified(100);unclassified(100);unclassified(100);unclassified(100);unclassified(100);
Otu123	1	0	Bacteria(100);Proteobacteria(100);Gammaproteobacteria(100);Methylococcaceae(100);Hyd24-01(100);unclassified(100);unclassified(100);unclassified(100);unclassified(100);unclassified(100);
Otu124	1	0	Bacteria(100);Lentisphaerae(100);Victivallales(100);Victivallaceae(100);Unclassified(100);unclassified(100);unclassified(100);unclassified(100);unclassified(100);unclassified(100);
Otu125	1	0	Bacteria(100);Proteobacteria(100);Gammaproteobacteria(100);Ferrimonadaceae(100);Unclassified(100);unclassified(100);unclassified(100);unclassified(100);unclassified(100);unclassified(100);
Otu126	1	1	Bacteria(100);Firmicutes(100);Mollicutes(100);Spiroplasma_citri(100);Unclassified(100);unclassified(100);unclassified(100);unclassified(100);unclassified(100);unclassified(100);
Otu127	1	1	Bacteria(100);Firmicutes(100);Clostridia(100);Peptostreptococcaceae(100);Peptoniphilus(100);Peptoniphilus_indolicus(100);unclassified(100);unclassified(100);unclassified(100);unclassified(100);
Otu128	1	2	Bacteria(100);Firmicutes(100);Desulfotomaculum(100);Desulfotomaculum_thermocisternum(100);Unclassified(100);unclassified(100);unclassified(100);unclassified(100);unclassified(100);unclassified(100);
Otu129	1	0	Bacteria(100);Verrucomicrobia(100);Spartobacteria(100);Chthoniobacter(100);unclassified(100);unclassified(100);unclassified(100);unclassified(100);unclassified(100);unclassified(100);
Otu130	1	2	Bacteria(100);WS6(100);Unclassified(100);unclassified(100);unclassified(100);unclassified(100);unclassified(100);unclassified(100);unclassified(100);unclassified(100);
Otu131	1	0	Bacteria(100);Firmicutes(100);Clostridia(100);Peptostreptococcaceae(100);Soehngenia(100);unclassified(100);unclassified(100);unclassified(100);unclassified(100);unclassified(100);
Otu132	81	0	Bacteria(100);Firmicutes(100);Clostridia(100);Clostridiales(100);Coprococcus(100);Unclassified(100);unclassified(100);unclassified(100);unclassified(100);unclassified(100);
Otu133	0	1	Bacteria(100);Proteobacteria(100);Gammaproteobacteria(100);Alteromonadales(100);Pseudoalteromonadaceae(100);Unclassified(100);unclassified(100);unclassified(100);unclassified(100);unclassified(100);
Otu134	0	1	Bacteria(100);Proteobacteria(100);Gammaproteobacteria(100);Moraxellaceae(100);Moraxella__Psychrobacter(100);Psychrobacter(100);unclassified(100);unclassified(100);unclassified(100);unclassified(100);
Otu135	5	2	Bacteria(100);Proteobacteria(100);Gammaproteobacteria(100);Betaproteobacteria(100);Comamonadaceae(100);Comamonas(100);Unclassified(100);unclassified(100);unclassified(100);unclassified(100);
Otu136	8	0	Bacteria(100);Firmicutes(100);Clostridia(100);Clostridiales(100);bacterium_MDA2477(100);unclassified(100);unclassified(100);unclassified(100);unclassified(100);unclassified(100);
Otu137	2	4	Bacteria(100);SR1(100);BH1(100);unclassified(100);unclassified(100);unclassified(100);unclassified(100);unclassified(100);unclassified(100);unclassified(100);
Otu138	1	4	Bacteria(100);Bacteroidetes(100);Sphingobacteriaceae(100);Sphingobacterium(100);Sphingobacterium_multivorum(100);unclassified(100);unclassified(100);unclassified(100);unclassified(100);unclassified(100);
Otu139	5	0	Bacteria(100);Bacteroidetes(100);Bacteroidales(100);Tannerellaceae(100);unclassified(100);unclassified(100);unclassified(100);unclassified(100);unclassified(100);unclassified(100);
Otu140	0	3	Bacteria(100);Actinobacteria(100);Actinobacteridae(100);Brachybacterium(100);unclassified(100);unclassified(100);unclassified(100);unclassified(100);unclassified(100);unclassified(100);
Otu141	1	0	Bacteria(100);Bacteroidetes(100);Bacteroidales(100);Bacteroidaceae(100);B_coprocola(100);unclassified(100);unclassified(100);unclassified(100);unclassified(100);unclassified(100);
Otu142	0	4	Bacteria(100);Actinobacteria(100);Actinobacteridae(100);Rhodococcus_B(100);unclassified(100);unclassified(100);unclassified(100);unclassified(100);unclassified(100);unclassified(100);
Otu143	1	0	Bacteria(100);Actinobacteria(100);Acidimicrobidae(100);EB1017_group(100);Acidimicrobidae_bacterium_Ellin7143(100);Unclassified(100);unclassified(100);unclassified(100);unclassified(100);unclassified(100);
Otu144	3	0	Bacteria(100);Proteobacteria(100);Gammaproteobacteria(100);Betaproteobacteria(100);Nitrosomonadales(100);Unclassified(100);unclassified(100);unclassified(100);unclassified(100);unclassified(100);
Otu145	1	0	Bacteria(100);Nitrospirae(100);Thermodesulfovibrionales(100);Thermodesulfovibrionaceae(100);Unclassified(100);unclassified(100);unclassified(100);unclassified(100);unclassified(100);unclassified(100);
Otu146	0	3	Bacteria(100);Proteobacteria(100);Gammaproteobacteria(100);Betaproteobacteria(100);Alcaligenaceae(100);Unclassified(100);unclassified(100);unclassified(100);unclassified(100);unclassified(100);
Otu147	2	0	Bacteria(100);Bacteroidetes(100);Bacteroidales(100);p-184-o5(100);rc5-47(100);unclassified(100);unclassified(100);unclassified(100);unclassified(100);unclassified(100);
Otu148	4	3	Bacteria(100);Proteobacteria(100);Alphaproteobacteria(100);JP57(100);unclassified(100);unclassified(100);unclassified(100);unclassified(100);unclassified(100);unclassified(100);
Otu149	1	0	Bacteria(100);Proteobacteria(100);Gammaproteobacteria(100);Nevskiaceae(100);Unclassified(100);unclassified(100);unclassified(100);unclassified(100);unclassified(100);unclassified(100);
Otu150	1	0	Bacteria(100);Actinobacteria(100);Actinobacteridae(100);Propionibacterineae(100);Nocardioidaceae(100);unclassified(100);unclassified(100);unclassified(100);unclassified(100);unclassified(100);
Otu151	3	0	Bacteria(100);Proteobacteria(100);Gammaproteobacteria(100);Pseudomonadaceae(100);glacial_ice_bacterium_M3C47K-2(100);Pseudomonas_psychrotolerans(100);unclassified(100);unclassified(100);unclassified(100);unclassified(100);
Otu152	5	0	Bacteria(100);Proteobacteria(100);Epsilonproteobacteria(100);Helicobacterales(100);Helicobacteraceae(100);Wolinella(100);unclassified(100);unclassified(100);unclassified(100);unclassified(100);
Otu153	1	0	Bacteria(100);Firmicutes(100);Clostridia(100);Clostridiales(100);Clostridiaceae(100);pDH-A(100);unclassified(100);unclassified(100);unclassified(100);unclassified(100);
Otu154	0	1	Bacteria(100);Spirochaetes(100);Treponemaceae(100);Treponema(100);Rs-D01(100);unclassified(100);unclassified(100);unclassified(100);unclassified(100);unclassified(100);
Otu155	4	0	Bacteria(100);Firmicutes(100);Bacilli(100);Lactobacillales(100);Lactobacillaceae(100);Lactobacillus(100);Lactobacillus_delbrueckii(100);Unclassified(100);unclassified(100);unclassified(100);
Otu156	0	1	Bacteria(100);Proteobacteria(100);Gammaproteobacteria(100);Betaproteobacteria(100);Sutterellaceae(100);HuCA4(100);unclassified(100);unclassified(100);unclassified(100);unclassified(100);
Otu157	1	0	Bacteria(100);Proteobacteria(100);Alphaproteobacteria(100);Ellin314(100);Unclassified(100);unclassified(100);unclassified(100);unclassified(100);unclassified(100);unclassified(100);
Otu158	1	0	Bacteria(100);Firmicutes(100);Clostridia(100);Clostridiales(100);C21_k11(100);unclassified(100);unclassified(100);unclassified(100);unclassified(100);unclassified(100);
Otu159	0	1	Bacteria(100);Proteobacteria(100);Gammaproteobacteria(100);Betaproteobacteria(100);Comamonadaceae(100);Unclassified(100);unclassified(100);unclassified(100);unclassified(100);unclassified(100);
Otu160	6	3	Bacteria(100);Proteobacteria(100);Alphaproteobacteria(100);Sphingomonadales(100);Sphingobium(100);Unclassified(100);unclassified(100);unclassified(100);unclassified(100);unclassified(100);
Otu161	0	2	Bacteria(100);Acidobacteria(100);iii1-15(100);uncultivated_soil_bacterium_clone_C112(100);unclassified(100);unclassified(100);unclassified(100);unclassified(100);unclassified(100);unclassified(100);
Otu162	1	1	Bacteria(100);Actinobacteria(100);Actinobacteridae(100);Segniliparaceae(100);unclassified(100);unclassified(100);unclassified(100);unclassified(100);unclassified(100);unclassified(100);
Otu163	2	1	Bacteria(100);Firmicutes(100);Bacilli(100);Lactobacillales(100);Aerococcaceae(100);Unclassified(100);unclassified(100);unclassified(100);unclassified(100);unclassified(100);
Otu164	1	0	Bacteria(100);Proteobacteria(100);Alphaproteobacteria(100);Crabtreella(100);unclassified(100);unclassified(100);unclassified(100);unclassified(100);unclassified(100);unclassified(100);
Otu165	2	0	Bacteria(100);Firmicutes(100);Clostridia(100);Peptostreptococcaceae(100);Clostridium_felsineum(100);Clostridium_formicaceticum(100);unclassified(100);unclassified(100);unclassified(100);unclassified(100);

OTU	Number of reads		Greengenes taxonomic classification
	Wol-	Wol+	
Otu166	6	0	Bacteria(100);Bacteroidetes(100);Bacteroidales(100);Porphyromonadaceae(100);Unclassified(100);unclassified(100);unclassified(100);unclassified(100);unclassified(100);unclassified(100);
Otu167	3	0	Bacteria(100);Firmicutes(100);Acidaminococcaceae(100);Anaerospora(100);Unclassified(100);unclassified(100);unclassified(100);unclassified(100);unclassified(100);unclassified(100);
Otu168	2	0	Bacteria(100);Firmicutes(100);Unclassified(100);unclassified(100);unclassified(100);unclassified(100);unclassified(100);unclassified(100);unclassified(100);unclassified(100);
Otu169	1	0	Bacteria(100);Proteobacteria(100);Gammaproteobacteria(100);Chromatiaceae(100);Marichromatium(100);unclassified(100);unclassified(100);unclassified(100);unclassified(100);unclassified(100);
Otu170	4	0	Bacteria(100);Firmicutes(100);Clostridia(100);Clostridiales(100);Johnsonella(100);Eubacterium_cf_saburreum_oral_strain_C27KA(100);human_oral_bacterium_C73(100);unclassified(100);unclassified(100);unclassified(100);
Otu171	2	0	Bacteria(100);Actinobacteria(100);Actinobacteridae(100);Brevibacteriaceae(100);unclassified(100);unclassified(100);unclassified(100);unclassified(100);unclassified(100);unclassified(100);
Otu172	0	1	Bacteria(100);Spirochaetes(100);Unclassified(100);unclassified(100);unclassified(100);unclassified(100);unclassified(100);unclassified(100);unclassified(100);unclassified(100);
Otu173	2	0	Bacteria(100);Firmicutes(100);Acidaminococcaceae(100);Unclassified(100);unclassified(100);unclassified(100);unclassified(100);unclassified(100);unclassified(100);unclassified(100);
Otu174	1	0	Bacteria(100);Proteobacteria(100);Gammaproteobacteria(100);Aeromonadaceae(100);Tolumonas(100);unclassified(100);unclassified(100);unclassified(100);unclassified(100);unclassified(100);
Otu175	1	0	Bacteria(100);Firmicutes(100);Clostridia(100);Clostridiales(100);Clostridium_bolteae(100);Clostridium_bolteae(100);unclassified(100);unclassified(100);unclassified(100);unclassified(100);
Otu176	0	1	Bacteria(100);Proteobacteria(100);Gammaproteobacteria(100);Halomonadaceae(100);Unclassified(100);unclassified(100);unclassified(100);unclassified(100);unclassified(100);unclassified(100);
Otu177	2	0	Bacteria(100);Bacteroidetes(100);Bacteroidales(100);rumen_bacterium_YS3(100);unclassified(100);unclassified(100);unclassified(100);unclassified(100);unclassified(100);unclassified(100);
Otu178	1	0	Bacteria(100);Verrucomicrobia(100);Pedosphaerae(100);CTD005-1B-02(100);Unclassified(100);unclassified(100);unclassified(100);unclassified(100);unclassified(100);unclassified(100);
Otu179	0	7	Bacteria(100);Actinobacteria(100);Actinobacteridae(100);Gordoniaceae(100);Tsukamurella(100);unclassified(100);unclassified(100);unclassified(100);unclassified(100);unclassified(100);
Otu180	2	0	Bacteria(100);Proteobacteria(100);Gammaproteobacteria(100);Enterobacteriales_Enterobacteriaceae(100);Morganella(100);unclassified(100);unclassified(100);unclassified(100);unclassified(100);unclassified(100);
Otu181	3	0	Bacteria(100);Firmicutes(100);Bacilli(100);Lactobacillales(100);Leuconostoc(100);Leuconostoc_carnosum(100);unclassified(100);unclassified(100);unclassified(100);unclassified(100);
Otu182	1	0	Bacteria(100);Firmicutes(100);Clostridia(100);Clostridiales(100);Clostridiaceae(100);Clostridium_perfringens(100);unclassified(100);unclassified(100);unclassified(100);unclassified(100);
Otu183	0	2	Bacteria(100);Actinobacteria(100);Actinobacteridae(100);Propionibacterineae(100);Nocardioides(100);unclassified(100);unclassified(100);unclassified(100);unclassified(100);unclassified(100);
Otu184	2	0	Bacteria(100);Proteobacteria(100);Alphaproteobacteria(100);Sphingomonadales(100);Sphingomonas_trueperi(100);unclassified(100);unclassified(100);unclassified(100);unclassified(100);unclassified(100);
Otu185	5	2	Bacteria(100);Chlamydiae(100);Parachlamydiaceae(100);unclassified(100);unclassified(100);unclassified(100);unclassified(100);unclassified(100);unclassified(100);unclassified(100);
Otu186	1	0	Bacteria(100);Proteobacteria(100);Gammaproteobacteria(100);Vibrionaceae(100);Grimontia(100);unclassified(100);unclassified(100);unclassified(100);unclassified(100);unclassified(100);
Otu187	0	1	Bacteria(100);Chloroflexi(100);Anaerolineae(100);Anaerolineales(100);SB-34(100);unclassified(100);unclassified(100);unclassified(100);unclassified(100);unclassified(100);
Otu188	1	0	Bacteria(100);Proteobacteria(100);Gammaproteobacteria(100);ZA2333c(100);Unclassified(100);unclassified(100);unclassified(100);unclassified(100);unclassified(100);unclassified(100);
Otu189	1	0	Bacteria(100);Proteobacteria(100);Alphaproteobacteria(100);Defluvicoccus(100);LPB60(100);unclassified(100);unclassified(100);unclassified(100);unclassified(100);unclassified(100);
Otu190	0	1	Bacteria(100);Proteobacteria(100);Alphaproteobacteria(100);Rhodobacterales(100);Hyphomonadaceae(100);marine_bacterium_SCRIPPS_423(100);unclassified(100);unclassified(100);unclassified(100);unclassified(100);
Otu191	1	0	Bacteria(100);Proteobacteria(100);Gammaproteobacteria(100);Betaproteobacteria(100);Zoogloea(100);beta_proteobacterium_B5(100);unclassified(100);unclassified(100);unclassified(100);unclassified(100);
Otu192	0	1	Bacteria(100);Proteobacteria(100);Gammaproteobacteria(100);Enterobacteriales_Enterobacteriaceae(100);Plesiomonas(100);unclassified(100);unclassified(100);unclassified(100);unclassified(100);unclassified(100);
Otu193	9	0	Bacteria(100);Firmicutes(100);Clostridia(100);Clostridiales(100);Faecalibacterium(100);Subdoligranulum(100);unclassified(100);unclassified(100);unclassified(100);unclassified(100);
Otu194	0	1	Bacteria(100);Proteobacteria(100);Gammaproteobacteria(100);Halomonadaceae(100);Cobetia(100);unclassified(100);unclassified(100);unclassified(100);unclassified(100);unclassified(100);
Otu195	0	1	Bacteria(100);Actinobacteria(100);Actinobacteridae(100);Propionibacterineae(100);Propionibacterium(100);Propionibacteriaceae(100);unclassified(100);unclassified(100);unclassified(100);unclassified(100);
Otu196	1	0	Bacteria(100);Actinobacteria(100);Coriobacteridae(100);Coriobacteriales(100);Collinsella(100);Collinsella_aerofaciens(100);Unclassified(100);unclassified(100);unclassified(100);unclassified(100);
Otu197	2	0	Bacteria(100);Proteobacteria(100);Gammaproteobacteria(100);Shewanellaceae(100);Unclassified(100);unclassified(100);unclassified(100);unclassified(100);unclassified(100);unclassified(100);
Otu198	0	1	Bacteria(100);Actinobacteria(100);Acidimicrobidae(100);koll13(100);Unclassified(100);unclassified(100);unclassified(100);unclassified(100);unclassified(100);unclassified(100);
Otu199	4	0	Bacteria(100);Proteobacteria(100);Alphaproteobacteria(100);Caedibacteraceae(100);Unclassified(100);unclassified(100);unclassified(100);unclassified(100);unclassified(100);unclassified(100);
Otu200	0	1	Bacteria(100);Bacteroidetes(100);Thermonema(100);unclassified(100);unclassified(100);unclassified(100);unclassified(100);unclassified(100);unclassified(100);unclassified(100);
Otu201	1	0	Bacteria(100);Firmicutes(100);Clostridia(100);Clostridiales(100);U29-D06(100);Unclassified(100);unclassified(100);unclassified(100);unclassified(100);unclassified(100);
Otu202	0	2	Bacteria(100);Actinobacteria(100);Actinobacteridae(100);Propionibacterineae(100);Unclassified(100);unclassified(100);unclassified(100);unclassified(100);unclassified(100);unclassified(100);
Otu203	1	0	Bacteria(100);Proteobacteria(100);Gammaproteobacteria(100);Betaproteobacteria(100);Sutterellaceae(100);Unclassified(100);unclassified(100);unclassified(100);unclassified(100);unclassified(100);
Otu204	1	0	Bacteria(100);Actinobacteria(100);Actinobacteridae(100);Streptomycineae(100);Streptomyces(100);unclassified(100);unclassified(100);unclassified(100);unclassified(100);unclassified(100);
Otu205	1	0	Bacteria(100);Firmicutes(100);Symbiobacteria(100);Symbiobacterales(100);Symbiobacterium(100);Symbiobacterium_thermophilum(100);unclassified(100);unclassified(100);unclassified(100);unclassified(100);
Otu206	1	0	Bacteria(100);Firmicutes(100);Bacilli(100);Lactobacillales(100);Lactobacillaceae(100);Lactobacillus(100);Lactobacillus_delbrueckii(100);p-2370-55G5(100);unclassified(100);unclassified(100);
Otu207	0	1	Bacteria(100);Bacteroidetes(100);Crenotrichaceae(100);Rhodothermus(100);unclassified(100);unclassified(100);unclassified(100);unclassified(100);unclassified(100);unclassified(100);
Otu208	0	1	Bacteria(100);Actinobacteria(100);Actinobacteridae(100);Curtobacterium(100);unclassified(100);unclassified(100);unclassified(100);unclassified(100);unclassified(100);unclassified(100);
Otu209	1	0	Bacteria(100);Thermodesulfobacteria(100);Thermodesulfatatoraceae(100);Thermodesulfatator(100);unclassified(100);unclassified(100);unclassified(100);unclassified(100);unclassified(100);unclassified(100);
