# Supplementary material for: Endosymbiont Dominated Bacterial Communities in a Dwarf Spider
Source: PLoS One. 2015 Feb 23;10(2):e0117297. doi: 10.1371/journal.pone.0117297 (PMC4338242; doi:10.1371/journal.pone.0117297)
Supplement: S3 Information — Taxonomic classification was based on comparison of the most abundant sequence from each cluster against both the type and non-type and cultured and uncultured strain bacterial database using the Ribosomal Database Project (RDP; http://rdp.cme.msu.edu/, accessed 02/07/2013)(Cole et al. 2009). Taxonomy is based on the NCBI nomenclature. OTU’s were determined by phylotype analysis wherein sequences were clustered according to their match with sequences in the SILVA database (as implemented in MOTHUR v 1.29.0) using the Greengenes taxonomic classification. (PDF) [file pone.0117297.s003.pdf]

OTU	Number of reads		Taxonomy (NCBI nomenclature)	RDP classification	S_ab scor
	Wol-	Wol+			
Otu001	346830	392414	Candidatus Rhabdochlamydia porcellionis; RKPsHep; AY223862	Chlamydiae:Chlamydiales:unclassified_Chlamydiales	0.735
Otu002	29706	19864	endosymbiont of Metaseiulus occidentalis; pAJ233; AY635291	Sphingobacteria:Sphingobacteriales:unclassified_"Sphingobacteriales"	0.818
Otu003	126	0	uncultured Clostridiales bacterium; YNP_ObP_B94; DQ243771	Clostridia:Clostridiales:Ruminococcaceae:Butyricicoccus	1
Otu004	476	0	uncultured bacterium; RL176_aah43e09; DQ793803	Clostridia:Clostridiales:Ruminococcaceae:unclassified_Ruminococcaceae	0.937
Otu005	2	0	uncultured Ruminococcaceae bacterium; L-93; JX543456	Clostridia:Clostridiales:Ruminococcaceae:Anaerofilum	0.812
Otu006	339	2	uncultured bacterium; SHCB0510; JN697770	Clostridia:Clostridiales:Lachnospiraceae:Clostridium XlVa	1
Otu007	59847	10718	Rickettsia limoniae; Gent; AF322442	Alphaproteobacteria:Rickettsiales:Rickettsiaceae:Rickettsia	0.906
Otu008	0	20437	uncultured alpha proteobacterium; SHNY547; HM111424	Alphaproteobacteria:Rickettsiales:Anaplasmataceae:Anaplasma	0.619
Otu009	1785	0	uncultured bacterium; T6_3_15; EU828414	Bacteroidia:Bacteroidales:Bacteroidaceae:Bacteroides	1
Otu010	300	152	Candidatus Rhabdochlamydia porcellionis; RKPsHep; AY223862	Chlamydiae:Chlamydiales:unclassified_Chlamydiales	0.797
Otu011	94	0	uncultured bacterium; IIIA-3; AJ488093	Clostridia:Clostridiales:Ruminococcaceae:Oscillibacter	1
Otu012	115	152	Candidatus Rhabdochlamydia porcellionis; RKPsHep; AY223862	Chlamydiae:Chlamydiales:unclassified_Chlamydiales	0.649
Otu013	0	1	Candidatus Rhabdochlamydia porcellionis; RKPsHep; AY223862	Chlamydiae:Chlamydiales:unclassified_Chlamydiales	0.689
Otu014	2	0	uncultured bacterium; CFT211A12; DQ456247	Clostridia:Clostridiales:Ruminococcaceae:unclassified_Ruminococcaceae	1
Otu015	89	0	Desulfovibrio sp.; KRS1; X93146	Deltaproteobacteria:Desulfovibrionales:Desulfovibrionaceae:Desulfovibrio	1
Otu016	2	2	Candidatus Rhabdochlamydia porcellionis; RKPsHep; AY223862	Chlamydiae:Chlamydiales:unclassified_Chlamydiales	0.647
Otu017	65	7	Arcobacter butzleri (T); ATCC 49616; AY621116	Epsilonproteobacteria:Campylobacterales:Campylobacteraceae:Arcobacter	1
Otu018	78	92	Candidatus Rhabdochlamydia porcellionis; RKPsHep; AY223862	Chlamydiae:Chlamydiales:unclassified_Chlamydiales	0.715
Otu019	97	4	Stenotrophomonas maltophilia; VUN10075; AF137357	Gammaproteobacteria:Xanthomonadales:Xanthomonadaceae:Stenotrophomonas	1
Otu020	300	5	Pseudomonas otitidis (T); MCC10330; AY953147	Gammaproteobacteria:Pseudomonadales:Pseudomonadaceae:Pseudomonas	0.967
Otu021	96	1	uncultured bacterium; OTU6; AB294747	Clostridia:Clostridiales:Ruminococcaceae:unclassified_Ruminococcaceae	1
Otu022	34	26	Candidatus Rhabdochlamydia porcellionis; RKPsHep; AY223862	Chlamydiae:Chlamydiales:unclassified_Chlamydiales	0.621
Otu023	58	254	uncultured bacterium; aab40c11; DQ818969	Gammaproteobacteria:Enterobacteriales:Enterobacteriaceae:Escherichia/Shigella	0.914
Otu024	164	1	Flavobacteriaceae genomosp. C1; C4MKM119; AY278614	Flavobacteria:Flavobacteriales:Flavobacteriaceae:Cloacibacterium	1
Otu025	5	3	Rickettsia endosymbiont of Torix tagoi; AB066351	Alphaproteobacteria:Rickettsiales:Rickettsiaceae:Rickettsia	0.634
Otu026	153	0	uncultured bacterium; KSB28; JX047081	Clostridia:Clostridiales:Clostridiales_Incertae Sedis XIII:Anaerovorax	1
Otu027	8	39	Cardinium endosymbiont of Marietta sp.; AY327470	Sphingobacteria:Sphingobacteriales:unclassified_"Sphingobacteriales"	0.68
Otu028	4	21	Paracoccus marcusii (T); Y12703	Alphaproteobacteria:Rhodobacterales:Rhodobacteraceae:Paracoccus	1
Otu029	1	1	uncultured bacterium; SRRB08; AB240501	Deltaproteobacteria:Bdellovibrionales:Bdellovibrionaceae:Vampirovibrio	0.732
Otu030	55	81	Candidatus Rhabdochlamydia porcellionis; RKPsHep; AY223862	Chlamydiae:Chlamydiales:unclassified_Chlamydiales	0.739
Otu031	30	0	uncultured bacterium; nbw788h02c1; GQ010923	Clostridia:Clostridiales:Ruminococcaceae:Clostridium IV	1
Otu032	29	0	Xanthomonas theicola (T); LMG 8684 T; Y10763	Gammaproteobacteria:Xanthomonadales:Xanthomonadaceae:Xanthomonas	1
Otu033	10	6	Candidatus Rhabdochlamydia porcellionis; RKPsHep; AY223862	Chlamydiae:Chlamydiales:unclassified_Chlamydiales	0.682
Otu034	3	6	Candidatus Rhabdochlamydia porcellionis; RKPsHep; AY223862	Chlamydiae:Chlamydiales:unclassified_Chlamydiales	0.614
Otu035	12	0	Kocuria rhizophila; JPL-9; AY030315	Actinobacteria:Actinobacteridae:Actinomycetales:Micrococcineae	1
Otu036	245	0	uncultured bacterium; nbw37d10c1; GQ059570	Bacteroidia:Bacteroidales:Porphyromonadaceae:unclassified_"Porphyromonadaceae"	1
Otu037	0	1	Candidatus Rhabdochlamydia porcellionis; RKPsHep; AY223862	Chlamydiae:Chlamydiales:unclassified_Chlamydiales	0.582
Otu038	0	3	uncultured Acidobacteria bacterium; AKYG1698; AY921973	Acidobacteria_Gp4:Gp4:	0.969
Otu039	7	0	uncultured bacterium; p-195-o5; AF371561	Clostridia:Clostridiales:Lachnospiraceae:Lachnospiracea_incertae_sedis	1
Otu040	1	0	uncultured Xanthomonadaceae bacterium; AMEG10; AM935344	Gammaproteobacteria:Xanthomonadales:Sinobacteraceae:unclassified_Sinobacteraceae	0.852
Otu041	0	8	bacterium str. 96446; AF227855	Alphaproteobacteria:Rhodospirillales:Acetobacteraceae:Roseomonas	1

OTU	Number of reads		Taxonomy (NCBI nomenclature)	RDP classification	S_ab scor
	Wol-	Wol+			
Otu042	1	0	Candidatus Rhabdochlamydia porcellionis; RKPsHep; AY223862	Chlamydiae:Chlamydiales:unclassified_Chlamydiales	0.497
Otu043	7	3	uncultured bacterium; ncd761h06c1; HM295809	Actinobacteria:Actinobacteridae:Actinomycetales:Corynebacterineae	1
Otu044	151	133	Rhabdochlamydia crassificans; CRIB01; AY928092	Chlamydiae:Chlamydiales:Parachlamydiaceae:Parachlamydia	0.58
Otu045	42	0	uncultured bacterium; G267; AY916285	Clostridia:Clostridiales:Ruminococcaceae:unclassified_Ruminococcaceae	1
Otu046	11	0	uncultured bacterium; T6_3_15; EU828414	Bacteroidia:Bacteroidales:Bacteroidaceae:Bacteroides	0.748
Otu047	0	7	uncultured bacterium; ncd859g08c1; HM297941	Actinobacteria:Actinobacteridae:Actinomycetales:Corynebacterineae:family	1
Otu048	10	0	Clostridium sp. r53; AB470961	Clostridia:Clostridiales:Clostridiaceae 1:Clostridium sensu stricto	0.891
Otu049	0	1	Candidatus Rhabdochlamydia porcellionis; RKPsHep; AY223862	Chlamydiae:Chlamydiales:unclassified_Chlamydiales	0.612
Otu050	4	16	Micrococcus luteus; D7; AJ409095	Actinobacteria:Actinobacteridae:Actinomycetales:Micrococcineae:family	1
Otu051	62	40	Moraxella osloensis; Ben 58; X95304	Gammaproteobacteria:Pseudomonadales:Moraxellaceae:Enhydrobacter	1
Otu052	42	1	uncultured bacterium; CR_52; JX457312	Erysipelotrichia:Erysipelotrichales:Erysipelotrichaceae:unclassified_Erysipelotrichaceae	1
Otu053	74	0	uncultured bacterium; IA-22; AJ488073	Epsilonproteobacteria:Campylobacterales:Campylobacteraceae:Sulfurospirillum	1
Otu054	8	0	uncultured bacterium; WYSK 0002; AY179349	Clostridia:Clostridiales:Lachnospiraceae:Clostridium XlVa	0.868
Otu055	7	0	Clostridium ramosum; M91; AY699288	Erysipelotrichia:Erysipelotrichales:Erysipelotrichaceae:Clostridium XVIII:	1
Otu056	33	43	Candidatus Rhabdochlamydia porcellionis; RKPsHep; AY223862	Chlamydiae:Chlamydiales:unclassified_Chlamydiales	0.549
Otu057	8	16	uncultured bacterium; nbt83g01; EU538245	Actinobacteria:Actinobacteridae:Actinomycetales:Propionibacterineae:family	0.835
Otu058	0	2	uncultured bacterium; nbw761b04c1; GQ014275	Clostridia:Clostridiales:Clostridiales_Incertae Sedis XI:unclassified_Clostridiales_Incertae Sed	1
Otu059	47	0	uncultured bacterium mle1-2; AF280841	Bacteroidia:Bacteroidales:Porphyromonadaceae:Parabacteroides	1
Otu060	3	0	uncultured bacterium; T6_3_15; EU828414	Bacteroidia:Bacteroidales:Bacteroidaceae:Bacteroides	0.823
Otu061	2	1	Candidatus Rhabdochlamydia porcellionis; RKPsHep; AY223862	Chlamydiae:Chlamydiales:unclassified_Chlamydiales	0.735
Otu062	4	0	uncultured bacterium; ncd650c12c1; HM287878	Gammaproteobacteria:Pseudomonadales:Moraxellaceae:Acinetobacter	1
Otu063	10	16	Candidatus Rhabdochlamydia porcellionis; RKPsHep; AY223862	Chlamydiae:Chlamydiales:unclassified_Chlamydiales	0.638
Otu064	7	2	Candidatus Rhabdochlamydia porcellionis; RKPsHep; AY223862	Chlamydiae:Chlamydiales:unclassified_Chlamydiales	0.691
Otu065	1	0	Candidatus Rhabdochlamydia porcellionis; RKPsHep; AY223862	Chlamydiae:Chlamydiales:unclassified_Chlamydiales	0.654
Otu066	1	1	uncultured Citrobacter sp.; F1Sjun.16; GQ417179	Gammaproteobacteria:Enterobacteriales:Enterobacteriaceae:Citrobacter	0.868
Otu067	3	0	uncultured bacterium; COL_aai14b01; EU460073	Clostridia:Clostridiales:Lachnospiraceae:unclassified_Lachnospiraceae	0.795
Otu068	6	0	Aeromonas hydrophila (T); type strain: LMG 19562; AJ508765	Gammaproteobacteria:Aeromonadales:Aeromonadaceae:Aeromonas	1
Otu069	23	0	Acetobacterium woodii (T); DSM 1030; X96954	Clostridia:Clostridiales:Eubacteriaceae:Acetobacterium	1
Otu070	21	22	Candidatus Rhabdochlamydia porcellionis; RKPsHep; AY223862	Chlamydiae:Chlamydiales:unclassified_Chlamydiales	0.641
Otu071	31	4	uncultured Clostridium sp.; F117; DQ232857	Clostridia:Clostridiales:unclassified_Clostridiales	1
Otu072	13	11	Candidatus Rhabdochlamydia porcellionis; RKPsHep; AY223862	Chlamydiae:Chlamydiales:unclassified_Chlamydiales	0.49
Otu073	18	18	Staphylococcus caprae; DSM 20608; Y12593	Bacilli:Bacillales:Staphylococcaceae:Staphylococcus	1
Otu074	9	19	Candidatus Rhabdochlamydia porcellionis; RKPsHep; AY223862	Chlamydiae:Chlamydiales:unclassified_Chlamydiales	0.641
Otu075	30	0	uncultured bacterium; NBBPI0209_14; JQ072511	Clostridia:Clostridiales:Lachnospiraceae:Clostridium XlVb	1
Otu076	1	0	Rickettsia endosymbiont of Torix tagoi; AB066351	Alphaproteobacteria:Rickettsiales:Rickettsiaceae:Rickettsia	0.636
Otu077	42	3	uncultured bacterium; nbu507d04c1; GQ047565	Flavobacteria:Flavobacteriales:Flavobacteriaceae:Cloacibacterium	1
Otu078	9	1	uncultured Tolumonas sp.; R15-18; JF808898	Gammaproteobacteria:Aeromonadales:Aeromonadaceae:Tolumonas	1
Otu079	5	4	Candidatus Rhabdochlamydia porcellionis; RKPsHep; AY223862	Chlamydiae:Chlamydiales:unclassified_Chlamydiales	0.636
Otu080	9	2	uncultured bacterium; bat_aaj06h08; EU463921	Chloroplast:Chloroplast:Streptophyta	1
Otu081	0	26	Rickettsia endosymbiont of Torix tagoi; AB066351	Alphaproteobacteria:Rickettsiales:Rickettsiaceae:Rickettsia	0.752
Otu082	13	2	uncultured bacterium; 30g04; EF515599	Bacteroidia:Bacteroidales:Prevotellaceae:Prevotella	1

OTU	Number of reads		Taxonomy (NCBI nomenclature)	RDP classification	S_ab scor
	Wol-	Wol+			
Otu083	9	1	uncultured actinobacterium; MspS113; AY770698	Actinobacteria:Actinobacteridae:Actinomycetales:unclassified_Actinomycetales	1
Otu084	4	0	uncultured bacterium; E-23; KC835942	Clostridia:Clostridiales:Ruminococcaceae:unclassified_Ruminococcaceae	0.937
Otu085	2	5	Candidatus Rhabdochlamydia porcellionis; RKPsHep; AY223862	Chlamydiae:Chlamydiales:unclassified_Chlamydiales	0.379
Otu086	1	3	Candidatus Rhabdochlamydia porcellionis; RKPsHep; AY223862	Chlamydiae:Chlamydiales:unclassified_Chlamydiales	0.673
Otu087	0	4	Candidatus Rhabdochlamydia porcellionis; RKPsHep; AY223862	Chlamydiae:Chlamydiales:unclassified_Chlamydiales	0.66
Otu088	13	10	Candidatus Rhabdochlamydia porcellionis; RKPsHep; AY223862	Chlamydiae:Chlamydiales:unclassified_Chlamydiales	0.765
Otu089	6	2	Candidatus Rhabdochlamydia porcellionis; RKPsHep; AY223862	Chlamydiae:Chlamydiales:unclassified_Chlamydiales	0.638
Otu090	6	12	Candidatus Rhabdochlamydia porcellionis; RKPsHep; AY223862	Chlamydiae:Chlamydiales:unclassified_Chlamydiales	0.543
Otu091	10	12	Candidatus Rhabdochlamydia porcellionis; RKPsHep; AY223862	Chlamydiae:Chlamydiales:unclassified_Chlamydiales	0.507
Otu092	4	2	Candidatus Rhabdochlamydia porcellionis; RKPsHep; AY223862	Chlamydiae:Chlamydiales:unclassified_Chlamydiales	0.517
Otu093	7	8	Candidatus Rhabdochlamydia porcellionis; RKPsHep; AY223862	Chlamydiae:Chlamydiales:unclassified_Chlamydiales	0.447
Otu094	8	4	Candidatus Rhabdochlamydia porcellionis; RKPsHep; AY223862	Chlamydiae:Chlamydiales:unclassified_Chlamydiales	0.533
Otu095	2	0	Candidatus Rhabdochlamydia porcellionis; RKPsHep; AY223862	Chlamydiae:Chlamydiales:unclassified_Chlamydiales	0.418
Otu096	1	0	Rickettsia endosymbiont of Torix tagoi; AB066351	Alphaproteobacteria:Rickettsiales:Rickettsiaceae:Rickettsia	0.769
Otu097	1	0	Candidatus Rhabdochlamydia porcellionis; RKPsHep; AY223862	Chlamydiae:Chlamydiales:unclassified_Chlamydiales	0.601
Otu098	1	1	Candidatus Rhabdochlamydia porcellionis; RKPsHep; AY223862	Chlamydiae:Chlamydiales:unclassified_Chlamydiales	0.48
Otu099	1	1	Candidatus Rhabdochlamydia porcellionis; RKPsHep; AY223862	Chlamydiae:Chlamydiales:unclassified_Chlamydiales	0.438
Otu100	1	3	Candidatus Rhabdochlamydia sp. cvE88; JF513056	Chlamydiae:Chlamydiales:unclassified_Chlamydiales	0.477
Otu101	3	2	Candidatus Rhabdochlamydia porcellionis; RKPsHep; AY223862	Chlamydiae:Chlamydiales:unclassified_Chlamydiales	0.586
Otu102	1	0	Candidatus Rhabdochlamydia porcellionis; RKPsHep; AY223862	Chlamydiae:Chlamydiales:unclassified_Chlamydiales	0.579
Otu103	0	3	Candidatus Rhabdochlamydia porcellionis; RKPsHep; AY223862	Chlamydiae:Chlamydiales:unclassified_Chlamydiales	0.553
Otu104	1	0	Candidatus Rhabdochlamydia porcellionis; RKPsHep; AY223862	Chlamydiae:Chlamydiales:unclassified_Chlamydiales	0.396
Otu105	1	0	Rickettsia endosymbiont of Torix tagoi; AB066351	Alphaproteobacteria:Rickettsiales:Rickettsiaceae:Rickettsia	0.669
Otu106	7	0	uncultured bacterium; 16slp101-2g08.p1k; GQ157097	Gammaproteobacteria:Pseudomonadales:Pseudomonadaceae:Pseudomonas	0.739
Otu107	1	1	Candidatus Rhabdochlamydia porcellionis; RKPsHep; AY223862	Chlamydiae:Chlamydiales:unclassified_Chlamydiales	0.48
Otu108	1	1	Candidatus Rhabdochlamydia porcellionis; RKPsHep; AY223862	Chlamydiae:Chlamydiales:unclassified_Chlamydiales	0.507
Otu109	1	0	Candidatus Rhabdochlamydia porcellionis; RKPsHep; AY223862	Chlamydiae:Chlamydiales:unclassified_Chlamydiales	0.543
Otu110	0	1	Candidatus Rhabdochlamydia porcellionis; RKPsHep; AY223862	Chlamydiae:Chlamydiales:unclassified_Chlamydiales	0.516
Otu111	7	0	Rickettsia endosymbiont of Hemiclepsis marginata; AB113215	Alphaproteobacteria:Rickettsiales:Rickettsiaceae:Rickettsia	0.669
Otu112	1	2	Candidatus Rhabdochlamydia porcellionis; RKPsHep; AY223862	Chlamydiae:Chlamydiales:unclassified_Chlamydiales	0.49
Otu113	2	0	Candidatus Rhabdochlamydia porcellionis; RKPsHep; AY223862	Chlamydiae:Chlamydiales:unclassified_Chlamydiales	0.523
Otu114	1	1	Candidatus Rhabdochlamydia porcellionis; RKPsHep; AY223862	Chlamydiae:Chlamydiales:unclassified_Chlamydiales	0.503
Otu115	8	0	Yersinia frederiksenii (T); ATCC 33641; AF366379	Gammaproteobacteria:Enterobacteriales:Enterobacteriaceae:Yersinia	0.724
Otu116	5	0	Rickettsia endosymbiont of Torix tagoi; AB066351	Alphaproteobacteria:Rickettsiales:Rickettsiaceae:Rickettsia	0.829
Otu117	2	1	Candidatus Rhabdochlamydia porcellionis; RKPsHep; AY223862	Chlamydiae:Chlamydiales:unclassified_Chlamydiales	0.47
Otu118	1	0	uncultured bacterium; nbw37d10c1; GQ059570	Bacteroidia:Bacteroidales:Porphyromonadaceae:unclassified_"Porphyromonadaceae"	0.694
Otu119	1	0	Candidatus Rhabdochlamydia porcellionis; RKPsHep; AY223862	Chlamydiae:Chlamydiales:unclassified_Chlamydiales	0.516
Otu120	1	1	Candidatus Rhabdochlamydia porcellionis; RKPsHep; AY223862	Chlamydiae:Chlamydiales:unclassified_Chlamydiales	0.631
Otu121	4	1	Candidatus Rhabdochlamydia porcellionis; RKPsHep; AY223862	Chlamydiae:Chlamydiales:unclassified_Chlamydiales	0.507
Otu122	2	2	Candidatus Rhabdochlamydia porcellionis; RKPsHep; AY223862	Chlamydiae:Chlamydiales:unclassified_Chlamydiales	0.533
Otu123	1	0	Rickettsia endosymbiont of Torix tagoi; AB066351	Alphaproteobacteria:Rickettsiales:Rickettsiaceae:Rickettsia	0.633

OTU	Number of reads		Taxonomy (NCBI nomenclature)	RDP classification	S_ab scor
	Wol-	Wol+			
Otu124	1	0	Candidatus Rhabdochlamydia porcellionis; RKPsHep; AY223862	Chlamydiae:Chlamydiales:unclassified_Chlamydiales	0.537
Otu125	1	0	bacterium enrichment culture clone JCN13; FJ009447	Gammaproteobacteria:Enterobacteriales:Enterobacteriaceae:Serratia	0.837
Otu126	1	1	Candidatus Rhabdochlamydia porcellionis; RKPsHep; AY223862	Chlamydiae:Chlamydiales:unclassified_Chlamydiales	0.454
Otu127	1	1	Candidatus Rhabdochlamydia porcellionis; RKPsHep; AY223862	Chlamydiae:Chlamydiales:unclassified_Chlamydiales	0.553
Otu128	1	2	Candidatus Rhabdochlamydia porcellionis; RKPsHep; AY223862	Chlamydiae:Chlamydiales:unclassified_Chlamydiales	0.627
Otu129	1	0	Candidatus Rhabdochlamydia porcellionis; RKPsHep; AY223862	Chlamydiae:Chlamydiales:unclassified_Chlamydiales	0.503
Otu130	1	2	Candidatus Rhabdochlamydia porcellionis; RKPsHep; AY223862	Chlamydiae:Chlamydiales:unclassified_Chlamydiales	0.5
Otu131	1	0	Candidatus Rhabdochlamydia porcellionis; RKPsHep; AY223862	Chlamydiae:Chlamydiales:unclassified_Chlamydiales	0.553
Otu132	81	0	Clostridium herbivorans (T); 54408; L34418	Clostridia:Clostridiales:Lachnospiraceae:Clostridium XlVa	1
Otu133	0	1	Pseudoalteromonas sp. D17; AY582935	Gammaproteobacteria:Alteromonadales:Pseudoalteromonadaceae:Pseudoalteromonas	0.893
Otu134	0	1	Hyphomicrobium sp. wp14; AJ551095	Gammaproteobacteria:Pseudomonadales:Moraxellaceae:Psychrobacter	1
Otu135	5	2	bacterium SRMC-52-8; DQ104970	Betaproteobacteria:Burkholderiales:Comamonadaceae:Comamonas	1
Otu136	8	0	uncultured bacterium; C9.26; GU559765	Clostridia:Clostridiales:Lachnospiraceae:Butyrivibrio	1
Otu137	2	4	Candidatus Rhabdochlamydia porcellionis; RKPsHep; AY223862	Chlamydiae:Chlamydiales:unclassified_Chlamydiales	0.608
Otu138	1	4	Sphingobacterium multivorum (T); IAM14316; AB100738	Sphingobacteria:Sphingobacteriales:Sphingobacteriaceae:Sphingobacterium	0.912
Otu139	5	0	uncultured bacterium; C1O; DQ856517	Bacteroidia:Bacteroidales:Porphyromonadaceae:Dysgonomonas	0.959
Otu140	0	3	Brachybacterium faecium; DSM 4810; X91032	Actinobacteria:Actinobacteridae:Actinomycetales:Micrococcineae:family	1
Otu141	1	0	uncultured bacterium; T6_3_15; EU828414	Bacteroidia:Bacteroidales:Bacteroidaceae:Bacteroides	0.789
Otu142	0	4	Rhodococcus fascians; DSM 43673; X79187	Actinobacteria:Actinobacteridae:Actinomycetales:Corynebacterineae:family	1
Otu143	1	0	Candidatus Rhabdochlamydia porcellionis; RKPsHep; AY223862	Chlamydiae:Chlamydiales:unclassified_Chlamydiales	0.461
Otu144	3	0	Nitrosomonas ureae; AF272414	Betaproteobacteria:Nitrosomonadales:Nitrosomonadaceae:unclassified_Nitrosomonadacea	0.96
Otu145	1	0	uncultured bacterium; nbw731d05c1; GQ066992	Bacteroidia:Bacteroidales:Porphyromonadaceae:unclassified_"Porphyromonadaceae"	0.671
Otu146	0	3	Alcaligenes sp. TS-MOSK-6; AB234302	Betaproteobacteria:Burkholderiales:Alcaligenaceae:Achromobacter:	0.961
Otu147	2	0	uncultured bacterium; nbw37d10c1; GQ059570	Bacteroidia:Bacteroidales:Porphyromonadaceae:unclassified_"Porphyromonadaceae"	0.912
Otu148	4	3	alpha proteobacterium S22-37; AM932270	Alphaproteobacteria:Rhizobiales:unclassified_Rhizobiales	0.527
Otu149	1	0	uncultured Xanthomonadaceae bacterium; AMEG10; AM935344	Gammaproteobacteria:Xanthomonadales:Sinobacteraceae:unclassified_Sinobacteraceae	0.933
Otu150	1	0	uncultured Nocardioidaceae bacterium; Elev_16S_388; EF019220	Actinobacteria:Actinobacteridae:Actinomycetales:Propionibacterineae:family	1
Otu151	3	0	uncultured bacterium; LNR A2-4; DQ988257	Gammaproteobacteria:Pseudomonadales:Pseudomonadaceae:Pseudomonas	0.843
Otu152	5	0	Wolinella succinogenes (T); ATCC 29543; M88159	Epsilonproteobacteria:Campylobacterales:Helicobacteraceae:Wolinella	1
Otu153	1	0	Candidatus Rhabdochlamydia sp. cvE88; JF513056	Chlamydiae:Chlamydiales:unclassified_Chlamydiales	0.549
Otu154	0	1	Candidatus Rhabdochlamydia porcellionis; RKPsHep; AY223862	Chlamydiae:Chlamydiales:unclassified_Chlamydiales	0.366
Otu155	4	0	Lactobacillus fornicalis (T); TV1018; Y18654	Bacilli:Lactobacillales:Lactobacillaceae:Lactobacillus	1
Otu156	0	1	uncultured bacterium; N913; AY975480	Betaproteobacteria:Burkholderiales:Sutterellaceae:Parasutterella	1
Otu157	1	0	Rickettsia endosymbiont of Torix tagoi; AB066351	Alphaproteobacteria:Rickettsiales:Rickettsiaceae:Rickettsia	0.742
Otu158	1	0	uncultured bacterium; 16slp112-2h09.p1k; GQ158172	Clostridia:Clostridiales:Lachnospiraceae:Lachnospiracea_incertae_sedis	0.867
Otu159	0	1	Tepidimonas arfidensis; AB206468	Betaproteobacteria:Burkholderiales:Burkholderiales_incertae_sedis:Tepidimonas	1
Otu160	6	3	uncultured alpha proteobacterium; JG37-AG-18; AJ518765	Alphaproteobacteria:Sphingomonadales:Sphingomonadaceae:Sphingomonas	1
Otu161	0	2	Candidatus Rhabdochlamydia porcellionis; RKPsHep; AY223862	Chlamydiae:Chlamydiales:unclassified_Chlamydiales	0.68
Otu162	1	1	Candidatus Rhabdochlamydia porcellionis; RKPsHep; AY223862	Chlamydiae:Chlamydiales:unclassified_Chlamydiales	0.586
Otu163	2	1	Firmicutes oral clone BX005; AY005049	Bacilli:Lactobacillales:Carnobacteriaceae:Atopostipes	1
Otu164	1	0	uncultured bacterium; Winter_MachineA&B_HEPAoff_4a01; FJ658472	Alphaproteobacteria:Rhizobiales:Brucellaceae:Ochrobactrum	0.953

OTU	Number of reads		Taxonomy (NCBI nomenclature)	RDP classification	S_ab scor
	Wol-	Wol+			
Otu165	2	0	Peptoniphilus duerdenii ATCC BAA-1640; WAL 1998L; EU526290	Clostridia:Clostridiales:Clostridiales_Incertae Sedis XI:Peptoniphilus	1
Otu166	6	0	uncultured bacterium; nbw37d10c1; GQ059570	Bacteroidia:Bacteroidales:Porphyromonadaceae:unclassified_"Porphyromonadaceae"	0.932
Otu167	3	0	uncultured bacterium; HuAC20; AY684401	Negativicutes:Selenomonadales:Veillonellaceae:unclassified_Veillonellaceae	1
Otu168	2	0	uncultured bacterium; copi29; AY563456	Negativicutes:Selenomonadales:Acidaminococcaceae:unclassified_Acidaminococcaceae	1
Otu169	1	0	Serratia marcescens; SA Ant16; AY551938	Gammaproteobacteria:Enterobacteriales:Enterobacteriaceae:Serratia	0.849
Otu170	4	0	uncultured organism; ELU0144-T169-S-IIPCRAMgANa_000147; HQ799464	Clostridia:Clostridiales:unclassified_Clostridiales	0.984
Otu171	2	0	Brevibacterium paucivorans (T); CF 62; AJ251463	Actinobacteria:Actinobacteridae:Actinomycetales:Micrococcineae:family	1
Otu172	0	1	Candidatus Rhabdochlamydia porcellionis; RKPsHep; AY223862	Chlamydiae:Chlamydiales:unclassified_Chlamydiales	0.351
Otu173	2	0	Anaeroarcus burkinensis (T); DSM 6283(T); AJ010961	Negativicutes:Selenomonadales:Veillonellaceae:Anaeroarcus	1
Otu174	1	0	uncultured Tolumonas sp.; R15-18; JF808898	Gammaproteobacteria:Aeromonadales:Aeromonadaceae:Tolumonas	0.921
Otu175	1	0	uncultured bacterium; K78S4_31f06; EU455391	Clostridia:Clostridiales:Lachnospiraceae:unclassified_Lachnospiraceae	0.945
Otu176	0	1	uncultured marine bacterium; BM1-1-48; FJ825816	Gammaproteobacteria:Oceanospirillales:Halomonadaceae:Halomonas	0.98
Otu177	2	0	uncultured bacterium; nbw37d10c1; GQ059570	Bacteroidia:Bacteroidales:Porphyromonadaceae:unclassified_"Porphyromonadaceae"	0.864
Otu178	1	0	Candidatus Rhabdochlamydia porcellionis; RKPsHep; AY223862	Chlamydiae:Chlamydiales:unclassified_Chlamydiales	0.373
Otu179	0	7	Tsukamurella spumae (T); M. Goodfellow N1171 (type strain); Z37150	Actinobacteria:Actinobacteridae:Actinomycetales:Corynebacterineae:family	1
Otu180	2	0	Enterobacter sp. 2390; JX174267	Gammaproteobacteria:Enterobacteriales:Enterobacteriaceae:Enterobacter	0.804
Otu181	3	0	Leuconostoc carnosum (T); NRIC 1722; AB022925	Bacilli:Lactobacillales:Leuconostocaceae:Leuconostoc	1
Otu182	1	0	uncultured bacterium; 03f06; GQ132402	Clostridia:Clostridiales:Ruminococcaceae:Acetivibrio	0.819
Otu183	0	2	Nocardioides sp. 2_4V; EF540473	Actinobacteria:Actinobacteridae:Actinomycetales:Propionibacterineae:family	1
Otu184	2	0	Sphingomonas sp. MN 122.2a; AJ313019	Alphaproteobacteria:Sphingomonadales:Sphingomonadaceae:Sphingomonas	1
Otu185	5	2	Candidatus Rhabdochlamydia porcellionis; RKPsHep; AY223862	Chlamydiae:Chlamydiales:unclassified_Chlamydiales	0.775
Otu186	1	0	Candidatus Rhabdochlamydia porcellionis; RKPsHep; AY223862	Chlamydiae:Chlamydiales:unclassified_Chlamydiales	0.658
Otu187	0	1	Rickettsia endosymbiont of Torix tagoi; AB066351	Alphaproteobacteria:Rickettsiales:Rickettsiaceae:Rickettsia	0.682
Otu188	1	0	Candidatus Rhabdochlamydia porcellionis; RKPsHep; AY223862	Chlamydiae:Chlamydiales:unclassified_Chlamydiales	0.601
Otu189	1	0	Rickettsia endosymbiont of Torix tagoi; AB066351	Alphaproteobacteria:Rickettsiales:Rickettsiaceae:Rickettsia	0.773
Otu190	0	1	Rickettsia endosymbiont of Torix tagoi; AB066351	Alphaproteobacteria:Rickettsiales:Rickettsiaceae:Rickettsia	0.727
Otu191	1	0	Candidatus Rhabdochlamydia porcellionis; RKPsHep; AY223862	Chlamydiae:Chlamydiales:unclassified_Chlamydiales	0.575
Otu192	0	1	uncultured bacterium; AFEL_aai30e03; EU464518	Gammaproteobacteria:Enterobacteriales:Enterobacteriaceae:Escherichia/Shigella	0.758
Otu193	9	0	uncultured bacterium; K559; AY976911	Clostridia:Clostridiales:Ruminococcaceae:unclassified_Ruminococcaceae	1
Otu194	0	1	Cobetia marina (T); DSM 4741; AJ306890	Gammaproteobacteria:Oceanospirillales:Halomonadaceae:Cobetia	0.953
Otu195	0	1	uncultured bacterium; D-66; AY676491	Actinobacteria:Actinobacteridae:Actinomycetales:Propionibacterineae:family	1
Otu196	1	0	Candidatus Rhabdochlamydia porcellionis; RKPsHep; AY223862	Chlamydiae:Chlamydiales:unclassified_Chlamydiales	0.529
Otu197	2	0	Shewanella putrefaciens; AB057660	Gammaproteobacteria:Alteromonadales:Shewanellaceae:Shewanella	1
Otu198	0	1	Candidatus Rhabdochlamydia porcellionis; RKPsHep; AY223862	Chlamydiae:Chlamydiales:unclassified_Chlamydiales	0.467
Otu199	4	0	unidentified bacterium; LWSR-14; AY345543	Alphaproteobacteria:Kiloniellales:Kiloniellaceae:Kiloniella	0.719
Otu200	0	1	Candidatus Rhabdochlamydia porcellionis; RKPsHep; AY223862	Chlamydiae:Chlamydiales:unclassified_Chlamydiales	0.634
Otu201	1	0	uncultured bacterium; T6_3_15; EU828414	Bacteroidia:Bacteroidales:Bacteroidaceae:Bacteroides	0.646
Otu202	0	2	unidentified eubacterium clone FJ21-A; U27856	Actinobacteria:Actinobacteridae:Actinomycetales:Propionibacterineae:family	1
Otu203	1	0	Tepidimonas aquatica (T); CLN-1; AY324139	Betaproteobacteria:Burkholderiales:Burkholderiales_incertae_sedis:Tepidimonas	0.974
Otu204	1	0	Kocuria varians (T); DSM 20033; X87754	Actinobacteria:Actinobacteridae:Actinomycetales:Micrococcineae:family	0.969
Otu205	1	0	Candidatus Rhabdochlamydia porcellionis; RKPsHep; AY223862	Chlamydiae:Chlamydiales:unclassified_Chlamydiales	0.507

OTU	Number of reads		Taxonomy (NCBI nomenclature)	RDP classification	S_ab scor
	Wol-	Wol+			
Otu206	1	0	uncultured bacterium; p-2370-55G5; AF371474	Bacilli:Lactobacillales:Lactobacillaceae:Lactobacillus	0.953
Otu207	0	1	Rickettsia endosymbiont of Torix tagoi; AB066351	Alphaproteobacteria:Rickettsiales:Rickettsiaceae:Rickettsia	0.47
Otu208	0	1	Curtobacterium pusillum (T); type strain: DSM 20527; AJ784400	Actinobacteria:Actinobacteridae:Actinomycetales:Micrococcineae:family	1
Otu209	1	0	Candidatus Rhabdochlamydia porcellionis; RKPsHep; AY223862	Chlamydiae:Chlamydiales:unclassified_Chlamydiales	0.418
Otu210	1	0	Streptococcus sp. oral clone FN051; AF432135	Bacilli:Lactobacillales:Streptococcaceae:Streptococcus	1
