# Supplementary material for: Endosymbiont Dominated Bacterial Communities in a Dwarf Spider
Source: PLoS One. 2015 Feb 23;10(2):e0117297. doi: 10.1371/journal.pone.0117297 (PMC4338242; doi:10.1371/journal.pone.0117297)
Supplement: S4 Information — OTU’s were determined by phylotype analysis wherein sequences were clustered according to their match with sequences in the SILVA database (as implemented in MOTHUR v 1.29.0) using the Greengenes taxonomic classification. (PDF) [file pone.0117297.s004.pdf]

OTU	Number of reads				Greengenes taxonomic classification			
	Wol-	Wol+	DAM	WAL				
Otu001	0	0	123	104	Bacteria(100);Proteobacteria(100);Gammaproteobacteria(100);Xanthomonadales(100);Frateuria(100);Unclassified(100);unclassified(100);unclassified(100);unclassified(100);unclassified(100);			
Otu002	369741	838377	651	771800	Bacteria(100);Chlamydiae(100);Simkaniaceae(100);unclassified(100);unclassified(100);unclassified(100);unclassified(100);unclassified(100);unclassified(100);unclassified(100);			
Otu003	113086	97931	415845	118093	Bacteria(100);Bacteroidetes(100);Cardiniaceae(100);Candidatus_Cardinium(100);unclassified(100);unclassified(100);unclassified(100);unclassified(100);unclassified(100);unclassified(100);			
Otu004	11	19	303654	79	Bacteria(100);Proteobacteria(100);Gammaproteobacteria(100);Moraxellaceae(100);Unclassified(100);unclassified(100);unclassified(100);unclassified(100);unclassified(100);unclassified(100);			
Otu005	67993	12641	102503	93446	Bacteria(100);Proteobacteria(100);Alphaproteobacteria(100);Consistiales(100);Rickettsiales(100);spotted_fever_group(100);unclassified(100);unclassified(100);unclassified(100);unclassified(100);			
Otu006	0	2	4	27	Bacteria(100);Bacteroidetes(100);Saprospirales(100);FukuS23(100);unclassified(100);unclassified(100);unclassified(100);unclassified(100);unclassified(100);unclassified(100);			
Otu007	3	64870	166081	92371	Bacteria(100);Proteobacteria(100);Alphaproteobacteria(100);Consistiales(100);Rickettsiales(100);Wolbachia(100);Unclassified(100);unclassified(100);unclassified(100);unclassified(100);			
Otu008	8	438	812	535	Bacteria(100);Proteobacteria(100);Alphaproteobacteria(100);Consistiales(100);Rickettsiales(100);Wolbachia(100);Wolbachia_endosymbiont_of_Onchocerca_ochengi(100);unclassified(100);unclassified(100);unclassified(100);			
Otu009	0	0	1	0	Bacteria(100);Actinobacteria(100);Acidimicrobidae(100);Microthrixineae(100);Candidatus_Microthrix(100);unclassified(100);unclassified(100);unclassified(100);unclassified(100);unclassified(100);			
Otu010	238	459	9	515	Bacteria(100);Acidobacteria(100);Acidobacteriae(100);K-5b2(100);Unclassified(100);unclassified(100);unclassified(100);unclassified(100);unclassified(100);unclassified(100);			
Otu011	364	827	6	786	Bacteria(100);Firmicutes(100);Clostridia(100);Clostridiales(100);Clostridium_viride(100);UC9-83(100);Unclassified(100);unclassified(100);unclassified(100);unclassified(100);			
Otu012	0	0	0	4	Bacteria(100);Firmicutes(100);Clostridia(100);Clostridiales(100);butyrate-producing_bacterium_A2-207(100);Unclassified(100);unclassified(100);unclassified(100);unclassified(100);unclassified(100);			
Otu013	7	1	13	8	Bacteria(100);Firmicutes(100);Clostridia(100);Peptostreptococcaceae(100);Frigovirgula_patagoniensis(100);Clostridium_sticklandii(100);unclassified(100);unclassified(100);unclassified(100);unclassified(100);			
Otu014	0	0	1	0	Bacteria(100);Fusobacteria(100);Propionigeniaceae(100);Unclassified(100);unclassified(100);unclassified(100);unclassified(100);unclassified(100);unclassified(100);unclassified(100);			
Otu015	0	2	70	129	Bacteria(100);Proteobacteria(100);Alphaproteobacteria(100);Caulobacterales(100);Brevundimonas(100);Unclassified(100);unclassified(100);unclassified(100);unclassified(100);unclassified(100);			
Otu016	18	61	3	54	Bacteria(100);Proteobacteria(100);Deltaproteobacteria(100);Desulfobacterium_catecholicum(100);Unclassified(100);unclassified(100);unclassified(100);unclassified(100);unclassified(100);unclassified(100);			
Otu017	0	0	3	0	Bacteria(100);Actinobacteria(100);Acidimicrobidae(100);Acidimicrobiaceae(100);unclassified(100);unclassified(100);unclassified(100);unclassified(100);unclassified(100);unclassified(100);			
Otu018	0	0	19	575	Bacteria(100);Bacteroidetes(100);Saprospirales(100);Unclassified(100);unclassified(100);unclassified(100);unclassified(100);unclassified(100);unclassified(100);unclassified(100);			
Otu019	0	0	233	0	Bacteria(100);Proteobacteria(100);Gammaproteobacteria(100);Enterobacteriales_Enterobacteriaceae(100);Plesiomonas(100);unclassified(100);unclassified(100);unclassified(100);unclassified(100);unclassified(100);			
Otu020	77	193	2	190	Bacteria(100);Spirochaetes(100);Treponemaceae(100);Treponema(100);RFS99(100);Unclassified(100);unclassified(100);unclassified(100);unclassified(100);unclassified(100);			
Otu021	0	0	326	0	Bacteria(100);Proteobacteria(100);Gammaproteobacteria(100);Piscirickettsiaceae(100);Thiobacillus_baregensis(100);Thiovirga(100);unclassified(100);unclassified(100);unclassified(100);unclassified(100);			
Otu022	0	0	5	3	Bacteria(100);Bacteroidetes(100);Bacteroidales(100);AU126(100);Rs-P01(100);unclassified(100);unclassified(100);unclassified(100);unclassified(100);unclassified(100);			
Otu023	7	10	3	11	Bacteria(100);Planctomycetes(100);Planctomycetacia(100);Pirellulales(100);unclassified(100);unclassified(100);unclassified(100);unclassified(100);unclassified(100);unclassified(100);			
Otu024	4	1	6	2	Bacteria(100);Proteobacteria(100);Alphaproteobacteria(100);Consistiales(100);Rickettsiales(100);Unclassified(100);unclassified(100);unclassified(100);unclassified(100);unclassified(100);			
Otu025	68	0	2	533	Bacteria(100);Firmicutes(100);Clostridia(100);Clostridiales(100);Clostridium_neopropionicum(100);unclassified(100);unclassified(100);unclassified(100);unclassified(100);unclassified(100);			
Otu026	2	1	10	1	Bacteria(100);Spirochaetes(100);Brevinema(100);unclassified(100);unclassified(100);unclassified(100);unclassified(100);unclassified(100);unclassified(100);unclassified(100);			
Otu027	1	5	9	4	Bacteria(100);Proteobacteria(100);Alphaproteobacteria(100);Bradyrhizobiales(100);Nitrobacter(100);unclassified(100);unclassified(100);unclassified(100);unclassified(100);unclassified(100);			
Otu028	0	0	3	2	Bacteria(100);Bacteroidetes(100);JTB248(100);unclassified(100);unclassified(100);unclassified(100);unclassified(100);unclassified(100);unclassified(100);unclassified(100);			
Otu029	6	6	4	6	Bacteria(100);MBMPE71(100);unclassified(100);unclassified(100);unclassified(100);unclassified(100);unclassified(100);unclassified(100);unclassified(100);unclassified(100);			
Otu030	0	1	6	3	Bacteria(100);Proteobacteria(100);Epsilonproteobacteria(100);Sulfurospirillaceae(100);Sulfurospirillum_arcachonense(100);unclassified(100);unclassified(100);unclassified(100);unclassified(100);unclassified(100);			
Otu031	114	280	1	293	Bacteria(100);Chlamydiae(100);Chlamydophila(100);Unclassified(100);unclassified(100);unclassified(100);unclassified(100);unclassified(100);unclassified(100);unclassified(100);			
Otu032	0	0	4	1	Bacteria(100);Proteobacteria(100);Alphaproteobacteria(100);S23_91(100);unclassified(100);unclassified(100);unclassified(100);unclassified(100);unclassified(100);unclassified(100);			
Otu033	89	10	119	126	Bacteria(100);Firmicutes(100);Clostridia(100);Peptostreptococcaceae(100);Clostridium_felsineum(100);Unclassified(100);unclassified(100);unclassified(100);unclassified(100);unclassified(100);			
Otu034	0	0	3335	12	Bacteria(100);Proteobacteria(100);Gammaproteobacteria(100);Enterobacteriales_Enterobacteriaceae(100);Unclassified(100);unclassified(100);unclassified(100);unclassified(100);unclassified(100);unclassified(100);			
Otu035	0	0	1	0	Bacteria(100);Chrysiogenetes(100);unclassified(100);unclassified(100);unclassified(100);unclassified(100);unclassified(100);unclassified(100);unclassified(100);unclassified(100);			
Otu036	3	0	12	6	Bacteria(100);NKB19(100);Unclassified(100);unclassified(100);unclassified(100);unclassified(100);unclassified(100);unclassified(100);unclassified(100);unclassified(100);			
Otu037	17	7	84	32	Bacteria(100);Firmicutes(100);Clostridia(100);Clostridiales(100);Faecalibacterium(100);Subdoligranulum(100);unclassified(100);unclassified(100);unclassified(100);unclassified(100);			
Otu038	8	13	0	18	Bacteria(100);Acidobacteria(100);PAUC37f(100);unclassified(100);unclassified(100);unclassified(100);unclassified(100);unclassified(100);unclassified(100);unclassified(100);			
Otu039	0	2	351	0	Bacteria(100);Proteobacteria(100);Gammaproteobacteria(100);Enterobacteriales_Enterobacteriaceae(100);Candidatus_Blochmannia(100);Blochmannia(100);unclassified(100);unclassified(100);unclassified(100);unclassified(100);			
Otu040	0	0	9	0	Bacteria(100);Proteobacteria(100);Gammaproteobacteria(100);Betaproteobacteria(100);Ralstoniaceae(100);Unclassified(100);unclassified(100);unclassified(100);unclassified(100);unclassified(100);			
Otu041	0	0	10	0	Bacteria(100);Verrucomicrobia(100);TP21(100);unclassified(100);unclassified(100);unclassified(100);unclassified(100);unclassified(100);unclassified(100);unclassified(100);			
Otu042	0	0	139	1	Bacteria(100);ZB3(100);Unclassified(100);unclassified(100);unclassified(100);unclassified(100);unclassified(100);unclassified(100);unclassified(100);unclassified(100);			
Otu043	0	0	14	0	Bacteria(100);Acidobacteria(100);iii1-15(100);Riz6I(100);Unclassified(100);unclassified(100);unclassified(100);unclassified(100);unclassified(100);unclassified(100);			
Otu044	2	1	28	5	Bacteria(100);Proteobacteria(100);Gammaproteobacteria(100);ZA3412c(100);Unclassified(100);unclassified(100);unclassified(100);unclassified(100);unclassified(100);unclassified(100);			
Otu045	0	1	1	0	Bacteria(100);Actinobacteria(100);Actinobacteridae(100);Gordoniaceae(100);Corynebacteriaceae(100);Corynebacterium_kroppenstedtii(100);unclassified(100);unclassified(100);unclassified(100);unclassified(100);			
Otu046	9	14	8	36	Bacteria(100);Proteobacteria(100);Deltaproteobacteria(100);Myxococcales(100);OM27(100);CTD005-73B-02(100);ctg_CGOF163(100);unclassified(100);unclassified(100);unclassified(100);			
Otu047	0	0	3	0	Bacteria(100);Proteobacteria(100);Gammaproteobacteria(100);Ferrimonadaceae(100);Unclassified(100);unclassified(100);unclassified(100);unclassified(100);unclassified(100);unclassified(100);			
Otu048	1	0	3	0	Bacteria(100);Firmicutes(100);Clostridia(100);Clostridiales(100);Clostridiaceae(100);Clostridium_autoethanogenum(100);unclassified(100);unclassified(100);unclassified(100);unclassified(100);			
Otu049	4	4	2	10	Bacteria(100);Haloanaerobiales(100);Halanaerobiaceae(100);Unclassified(100);unclassified(100);unclassified(100);unclassified(100);unclassified(100);unclassified(100);unclassified(100);			
Otu050	0	0	0	17	Bacteria(100);EM3(100);SRI-15(100);unclassified(100);unclassified(100);unclassified(100);unclassified(100);unclassified(100);unclassified(100);unclassified(100);			
Otu051	0	0	9	26	Bacteria(100);Proteobacteria(100);Gammaproteobacteria(100);Xanthomonadales(100);Frateuria(100);Rhodanobacter(100);unclassified(100);unclassified(100);unclassified(100);unclassified(100);			
Otu052	0	0	2	1	Bacteria(100);Proteobacteria(100);Epsilonproteobacteria(100);PeH32(100);unclassified(100);unclassified(100);unclassified(100);unclassified(100);unclassified(100);unclassified(100);			
Otu053	2	0	1	3	Bacteria(100);Bacteroidetes(100);Sphingobacteriaceae(100);Sphingobacterium(100);Unclassified(100);unclassified(100);unclassified(100);unclassified(100);unclassified(100);unclassified(100);			
Otu054	0	0	0	24	Bacteria(100);Firmicutes(100);Clostridia(100);Clostridiales(100);AN1045(100);unclassified(100);unclassified(100);unclassified(100);unclassified(100);unclassified(100);			
Otu055	0	0	64	0	Bacteria(100);Proteobacteria(100);Gammaproteobacteria(100);Enterobacteriales_Enterobacteriaceae(100);Candidatus_Blochmannia(100);Unclassified(100);unclassified(100);unclassified(100);unclassified(100);unclassified(100);			
Otu056	0	0	2	0	Bacteria(100);Firmicutes(100);Alicyclobacillus(100);Alicyclobacillus_acidocaldarius(100);Alicyclobacillus_tolerans(100);unclassified(100);unclassified(100);unclassified(100);unclassified(100);unclassified(100);			
Otu057	0	2	0	4	Bacteria(100);Proteobacteria(100);Alphaproteobacteria(100);H4(100);H29(100);unclassified(100);unclassified(100);unclassified(100);unclassified(100);unclassified(100);			
Otu058	2	5	0	131	Bacteria(100);Proteobacteria(100);Desulfurellales(100);Desulfurellaceae(100);Desulfurella(100);unclassified(100);unclassified(100);unclassified(100);unclassified(100);unclassified(100);			
Otu059	0	0	0	47	Bacteria(100);Firmicutes(100);Acidaminococcaceae(100);Unclassified(100);unclassified(100);unclassified(100);unclassified(100);unclassified(100);unclassified(100);unclassified(100);			
Otu060	1	5	50	124	Bacteria(100);Proteobacteria(100);Gammaproteobacteria(100);Pseudomonadaceae(100);Unclassified(100);unclassified(100);unclassified(100);unclassified(100);unclassified(100);unclassified(100);			
Otu061	6	1	8	9	Bacteria(100);Firmicutes(100);Bacilli(100);Lactobacillales(100);Aerococcaceae(100);Turicibacter(100);Turicibacter_sanguinis(100);NB1-n(100);unclassified(100);unclassified(100);			
Otu062	0	0	0	42	Bacteria(100);Firmicutes(100);Bacilli(100);Bacillus_cereus(100);unclassified(100);unclassified(100);unclassified(100);unclassified(100);unclassified(100);unclassified(100);			
Otu063	12	1	25	15	Bacteria(100);Firmicutes(100);Clostridia(100);Clostridiales(100);S-P1-30(100);unclassified(100);unclassified(100);unclassified(100);unclassified(100);unclassified(100);			
Otu064	0	0	14	165	Bacteria(100);Actinobacteria(100);Actinobacteridae(100);Propionibacterineae(100);Nocardioidaceae(100);unclassified(100);unclassified(100);unclassified(100);unclassified(100);unclassified(100);			
Otu065	5	17	1	23	Bacteria(100);Aquificae(100);Aquificae(100);Aquificales(100);Hydrogenobaculum(100);unclassified(100);unclassified(100);unclassified(100);unclassified(100);unclassified(100);			
Otu066	5	19	27	24	Bacteria(100);Proteobacteria(100);Epsilonproteobacteria(100);Helicobacterales(100);Helicobacteraceae(100);Helicobacter(100);Helicobacter_vulpecula(100);unclassified(100);unclassified(100);unclassified(100);			

OTU	Number of reads				Greengenes taxonomic classification
	Wol-	Wol+	DAM	WAL	
Otu067	1	2	4	5	Bacteria(100);Firmicutes(100);Clostridia(100);Clostridiales(100);Oxobacter(100);unclassified(100);unclassified(100);unclassified(100);unclassified(100);unclassified(100);
Otu068	0	0	69	0	Bacteria(100);Proteobacteria(100);Gammaproteobacteria(100);Moraxellaceae(100);Moraxella__Psychrobacter(100);Enhydrobacter(100);unclassified(100);unclassified(100);unclassified(100);unclassified(100);
Otu069	0	0	3	0	Bacteria(100);Proteobacteria(100);Gammaproteobacteria(100);Enterobacteriales_Enterobacteriaceae(100);Serratia_symbiotica(100);unclassified(100);unclassified(100);unclassified(100);unclassified(100);unclassified(100);
Otu070	0	0	310	34	Bacteria(100);Firmicutes(100);Clostridia(100);Clostridiales(100);Coprococcus(100);Unclassified(100);unclassified(100);unclassified(100);unclassified(100);unclassified(100);
Otu071	1	0	0	1	Bacteria(100);Acidobacteria(100);S035(100);unclassified(100);unclassified(100);unclassified(100);unclassified(100);unclassified(100);unclassified(100);unclassified(100);
Otu072	3	0	13	13	Bacteria(100);Firmicutes(100);Clostridia(100);Peptostreptococcaceae(100);Peptoniphilus(100);Anaerococcus(100);Unclassified(100);unclassified(100);unclassified(100);unclassified(100);
Otu073	3	0	9	30	Bacteria(100);Proteobacteria(100);Gammaproteobacteria(100);Xanthomonadales(100);Unclassified(100);unclassified(100);unclassified(100);unclassified(100);unclassified(100);unclassified(100);
Otu074	0	0	19	3	Bacteria(100);Firmicutes(100);Clostridia(100);Clostridiales(100);Clostridium_aminovalericum(100);unclassified(100);unclassified(100);unclassified(100);unclassified(100);unclassified(100);
Otu075	0	0	0	61	Bacteria(100);Planctomycetes(100);Planctomycetacia(100);Planctomycetales(100);Unclassified(100);unclassified(100);unclassified(100);unclassified(100);unclassified(100);unclassified(100);
Otu076	2	1	4	4	Bacteria(100);Firmicutes(100);Mollicutes(100);RF39(100);adhufec202(100);FM046(100);unclassified(100);unclassified(100);unclassified(100);unclassified(100);
Otu077	0	0	0	41	Bacteria(100);Verrucomicrobia(100);Spartobacteria(100);Chthoniobacter(100);unclassified(100);unclassified(100);unclassified(100);unclassified(100);unclassified(100);unclassified(100);
Otu078	0	0	0	23	Bacteria(100);Bacteroidetes(100);Pedobacter(100);unclassified(100);unclassified(100);unclassified(100);unclassified(100);unclassified(100);unclassified(100);unclassified(100);
Otu079	0	0	2	1	Bacteria(100);Proteobacteria(100);Alphaproteobacteria(100);Consistiales(100);Rickettsiales(100);Anaplasma(100);Unclassified(100);unclassified(100);unclassified(100);unclassified(100);
Otu080	0	0	4	0	Bacteria(100);OP10(100);SJA-176(100);unclassified(100);unclassified(100);unclassified(100);unclassified(100);unclassified(100);unclassified(100);unclassified(100);
Otu081	0	1	136	53	Bacteria(100);Firmicutes(100);Bacilli(100);Lactobacillales(100);Aerococcaceae(100);Abiotrophia(100);unclassified(100);unclassified(100);unclassified(100);unclassified(100);
Otu082	0	0	28	0	Bacteria(100);Proteobacteria(100);Gammaproteobacteria(100);Alteromonadales(100);Pseudoalteromonadaceae(100);Pseudoalteromonas_spongiae(100);unclassified(100);unclassified(100);unclassified(100);unclassified(100);
Otu083	0	0	18	18	Bacteria(100);Bacteroidetes(100);Saprospirales(100);BIsi8(100);unclassified(100);unclassified(100);unclassified(100);unclassified(100);unclassified(100);unclassified(100);
Otu084	0	4	4	1	Bacteria(100);Firmicutes(100);Mollicutes(100);Mycoplasmatales(100);Mycoplasma(100);Mycoplasma_flocculare(100);Unclassified(100);unclassified(100);unclassified(100);unclassified(100);
Otu085	1	2	0	2	Bacteria(100);Firmicutes(100);Clostridia(100);Clostridiales(100);p-3487-9F3(100);unclassified(100);unclassified(100);unclassified(100);unclassified(100);unclassified(100);
Otu086	0	0	17	0	Bacteria(100);Bacteroidetes(100);Bacteroidales(100);Marinilabiliaceae(100);Unclassified(100);unclassified(100);unclassified(100);unclassified(100);unclassified(100);unclassified(100);
Otu087	0	0	29	10	Bacteria(100);Proteobacteria(100);Alphaproteobacteria(100);Bradyrhizobiales(100);Bradyrhizobium_japonicum(100);Rhodopseudomonas_palustris(100);Unclassified(100);unclassified(100);unclassified(100);unclassified(100);
Otu088	3	0	1	4	Bacteria(100);Firmicutes(100);Mollicutes(100);Clostridium_aff_innocuum_CM970(100);Erysipelothrix(100);Unclassified(100);unclassified(100);unclassified(100);unclassified(100);unclassified(100);
Otu089	0	1	0	0	Bacteria(100);Planctomycetes(100);WPS-1(100);BD2-16(100);Unclassified(100);unclassified(100);unclassified(100);unclassified(100);unclassified(100);unclassified(100);
Otu090	0	2	2	3	Bacteria(100);Proteobacteria(100);Gammaproteobacteria(100);Betaproteobacteria(100);Sutterellaceae(100);Sutterella(100);unclassified(100);unclassified(100);unclassified(100);unclassified(100);
Otu091	1	1	1	5	Bacteria(100);Firmicutes(100);Clostridia(100);Peptostreptococcaceae(100);Peptoniphilus(100);Peptoniphilus_indolicus(100);unclassified(100);unclassified(100);unclassified(100);unclassified(100);
Otu092	0	0	35	2	Bacteria(100);Proteobacteria(100);Gammaproteobacteria(100);AEGEAN_245(100);unclassified(100);unclassified(100);unclassified(100);unclassified(100);unclassified(100);unclassified(100);
Otu093	0	0	1	0	Bacteria(100);Proteobacteria(100);Gammaproteobacteria(100);Chromatiaceae(100);Allochromatium(100);unclassified(100);unclassified(100);unclassified(100);unclassified(100);unclassified(100);
Otu094	1	1	6	1	Bacteria(100);Lentisphaerae(100);Victivallales(100);Victivallaceae(100);Unclassified(100);unclassified(100);unclassified(100);unclassified(100);unclassified(100);unclassified(100);
Otu095	0	0	22	1	Bacteria(100);Bacteroidetes(100);Saprospirales(100);cilia-associated_respiratory_bacterium_R3(100);unclassified(100);unclassified(100);unclassified(100);unclassified(100);unclassified(100);unclassified(100);
Otu096	0	0	0	12	Bacteria(100);Proteobacteria(100);Gammaproteobacteria(100);Xanthomonadales(100);Stenotrophomonas(100);unclassified(100);unclassified(100);unclassified(100);unclassified(100);unclassified(100);
Otu097	1	0	0	0	Bacteria(100);Firmicutes(100);Clostridia(100);Clostridiales(100);Ruminococcus(100);Rs-B44(100);unclassified(100);unclassified(100);unclassified(100);unclassified(100);
Otu098	0	0	2	0	Bacteria(100);Proteobacteria(100);Gammaproteobacteria(100);Enterobacteriales_Enterobacteriaceae(100);Baumannia(100);unclassified(100);unclassified(100);unclassified(100);unclassified(100);unclassified(100);
Otu099	2	0	18	0	Bacteria(100);Thermi(100);Thermales(100);Meiothermaceae(100);Meiothermus_silvanus(100);unclassified(100);unclassified(100);unclassified(100);unclassified(100);unclassified(100);
Otu100	1	2	1	6	Bacteria(100);Thermotogae(100);Unclassified(100);unclassified(100);unclassified(100);unclassified(100);unclassified(100);unclassified(100);unclassified(100);unclassified(100);
Otu101	0	0	0	15	Bacteria(100);Proteobacteria(100);Epsilonproteobacteria(100);Arcobacteraceae(100);Unclassified(100);unclassified(100);unclassified(100);unclassified(100);unclassified(100);unclassified(100);
Otu102	0	1	4	0	Bacteria(100);Chloroflexi(100);Anaerolineae(100);Anaerolineales(100);C1_B004(100);unclassified(100);unclassified(100);unclassified(100);unclassified(100);unclassified(100);
Otu103	0	0	5	1	Bacteria(100);Planctomycetes(100);Planctomycetacia(100);A17(100);PRR-7(100);BCf2-25(100);unclassified(100);unclassified(100);unclassified(100);unclassified(100);
Otu104	5	6	5	11	Bacteria(100);Planctomycetes(100);Planctomycetacia(100);DEL17(100);agg8(100);Unclassified(100);unclassified(100);unclassified(100);unclassified(100);unclassified(100);
Otu105	0	0	0	41	Bacteria(100);Bacteroidetes(100);Saprospirales(100);FukuN24(100);Unclassified(100);unclassified(100);unclassified(100);unclassified(100);unclassified(100);unclassified(100);
Otu106	0	0	21	0	Bacteria(100);Proteobacteria(100);Gammaproteobacteria(100);Halothiobacillaceae(100);Unclassified(100);unclassified(100);unclassified(100);unclassified(100);unclassified(100);unclassified(100);
Otu107	3	9	13	9	Bacteria(100);Proteobacteria(100);Gammaproteobacteria(100);Aeromonadaceae(100);Tolumonas(100);unclassified(100);unclassified(100);unclassified(100);unclassified(100);unclassified(100);
Otu108	6	0	18	0	Bacteria(100);Proteobacteria(100);Deltaproteobacteria(100);CTD005-82B-02(100);unclassified(100);unclassified(100);unclassified(100);unclassified(100);unclassified(100);unclassified(100);
Otu109	0	0	0	6	Bacteria(100);Bacteroidetes(100);Flavobacteriales(100);Unclassified(100);unclassified(100);unclassified(100);unclassified(100);unclassified(100);unclassified(100);unclassified(100);
Otu110	0	0	3	0	Bacteria(100);Actinobacteria(100);Actinobacteridae(100);Nesterenkonia(100);unclassified(100);unclassified(100);unclassified(100);unclassified(100);unclassified(100);unclassified(100);
Otu111	0	0	0	1	Bacteria(100);Gemmatimonadetes(100);Gemmatimonadales(100);Unclassified(100);unclassified(100);unclassified(100);unclassified(100);unclassified(100);unclassified(100);unclassified(100);
Otu112	2	1	19	21	Bacteria(100);Bacteroidetes(100);Saprospirales(100);FukuN24(100);GL182(100);unclassified(100);unclassified(100);unclassified(100);unclassified(100);unclassified(100);
Otu113	0	0	0	2	Bacteria(100);Gemmatimonadetes(100);Gemm-1b(100);Unclassified(100);unclassified(100);unclassified(100);unclassified(100);unclassified(100);unclassified(100);unclassified(100);
Otu114	1	0	1	0	Bacteria(100);Firmicutes(100);Clostridia(100);Clostridiales(100);Ruminococcus(100);PL-38B10(100);unclassified(100);unclassified(100);unclassified(100);unclassified(100);
Otu115	0	2	2	1	Bacteria(100);ZB2(100);BD5-13(100);BD5-13(100);unclassified(100);unclassified(100);unclassified(100);unclassified(100);unclassified(100);unclassified(100);
Otu116	3	5	2	2	Bacteria(100);Firmicutes(100);Clostridia(100);Clostridiales(100);Ruminococcus(100);p-1082-a5(100);Unclassified(100);unclassified(100);unclassified(100);unclassified(100);
Otu117	0	2	3	0	Bacteria(100);Bacteroidetes(100);Bacteroidales(100);Odoribacteriaceae(100);Unclassified(100);unclassified(100);unclassified(100);unclassified(100);unclassified(100);unclassified(100);
Otu118	2	0	4	1	Bacteria(100);Bacteroidetes(100);Thermonema(100);unclassified(100);unclassified(100);unclassified(100);unclassified(100);unclassified(100);unclassified(100);unclassified(100);
Otu119	0	0	3	1	Bacteria(100);Bacteroidetes(100);Flavobacteriales(100);Blattabacteriaceae(100);Unclassified(100);unclassified(100);unclassified(100);unclassified(100);unclassified(100);unclassified(100);
Otu120	0	0	1	0	Bacteria(100);ABY1_OD1(100);FW129(100);Unclassified(100);unclassified(100);unclassified(100);unclassified(100);unclassified(100);unclassified(100);unclassified(100);
Otu121	0	0	2	3	Bacteria(100);Bacteroidetes(100);Saprospirales(100);Terrimonaceae(100);Niabella(100);unclassified(100);unclassified(100);unclassified(100);unclassified(100);unclassified(100);
Otu122	0	0	0	1	Bacteria(100);Firmicutes(100);Clostridia(100);Peptostreptococcaceae(100);Peptostreptococcus_anaerobius(100);Unclassified(100);unclassified(100);unclassified(100);unclassified(100);unclassified(100);
Otu123	0	0	1	0	Bacteria(100);Chlorobi(100);Chlorobiales(100);Chlorobaculum(100);Unclassified(100);unclassified(100);unclassified(100);unclassified(100);unclassified(100);unclassified(100);
Otu124	0	0	35	0	Bacteria(100);Proteobacteria(100);Gammaproteobacteria(100);Betaproteobacteria(100);Rhodocyclales(100);beta_proteobacterium_F06002(100);IRD18C09(100);unclassified(100);unclassified(100);unclassified(100);
Otu125	4	6	2	9	Bacteria(100);Acidobacteria(100);Acidobacteriae(100);WJ7(100);unclassified(100);unclassified(100);unclassified(100);unclassified(100);unclassified(100);unclassified(100);
Otu126	0	0	2	0	Bacteria(100);Actinobacteria(100);Actinobacteridae(100);Intrasporangiaceae(100);Dermacoccus(100);unclassified(100);unclassified(100);unclassified(100);unclassified(100);unclassified(100);
Otu127	2	2	11	1	Bacteria(100);Proteobacteria(100);Alphaproteobacteria(100);Rhodobacterales(100);Rhodobacter(100);Unclassified(100);unclassified(100);unclassified(100);unclassified(100);unclassified(100);
Otu128	0	0	0	1	Bacteria(100);Actinobacteria(100);Actinobacteridae(100);Gordoniaceae(100);Unclassified(100);unclassified(100);unclassified(100);unclassified(100);unclassified(100);unclassified(100);
Otu129	0	0	2	1	Bacteria(100);Proteobacteria(100);Gammaproteobacteria(100);Xanthomonadales(100);Lysobacter(100);Silanimonas_lenta(100);unclassified(100);unclassified(100);unclassified(100);unclassified(100);
Otu130	0	0	3	0	Bacteria(100);WS3(100);Sediment-1(100);PRR-12(100);unclassified(100);unclassified(100);unclassified(100);unclassified(100);unclassified(100);unclassified(100);
Otu131	1	0	1	0	Bacteria(100);Firmicutes(100);Clostridia(100);Clostridiales(100);adhufec52(100);unclassified(100);unclassified(100);unclassified(100);unclassified(100);unclassified(100);
Otu132	0	1	2	0	Bacteria(100);Verrucomicrobia(100);Opitutae(100);Fucophilus(100);unclassified(100);unclassified(100);unclassified(100);unclassified(100);unclassified(100);unclassified(100);

OTU	Number of reads				Greengenes taxonomic classification
	Wol-	Wol+	DAM	WAL	
Otu133	0	1	2	1	Bacteria(100);Chlamydiae(100);Parachlamydiaceae(100);unclassified(100);unclassified(100);unclassified(100);unclassified(100);unclassified(100);unclassified(100);unclassified(100);
Otu134	1	2	0	2	Bacteria(100);Bacteroidetes(100);Bacteroidales(100);Bacteroidaceae(100);B_ovatus(100);unclassified(100);unclassified(100);unclassified(100);unclassified(100);unclassified(100);
Otu135	0	0	3	0	Bacteria(100);Bacteroidetes(100);Bacteroidales(100);adhufec77-25(100);Barnesiella(100);Barnesiella_viscericola(100);unclassified(100);unclassified(100);unclassified(100);unclassified(100);
Otu136	0	0	0	2	Bacteria(100);OP10(100);SJA-22(100);unclassified(100);unclassified(100);unclassified(100);unclassified(100);unclassified(100);unclassified(100);unclassified(100);
Otu137	0	0	33	0	Bacteria(100);Proteobacteria(100);Gammaproteobacteria(100);Piscirickettsiaceae(100);Thiobacillus_baregensis(100);MS149BH1062003_5(100);unclassified(100);unclassified(100);unclassified(100);unclassified(100);
Otu138	0	0	34	0	Bacteria(100);Firmicutes(100);Clostridia(100);Peptostreptococcaceae(100);Mogibacterium(100);CK047(100);p-648-a5(100);unclassified(100);unclassified(100);unclassified(100);
Otu139	0	0	1	1	Bacteria(100);Firmicutes(100);Clostridia(100);Clostridiales(100);Clostridiaceae(100);pDH-A(100);unclassified(100);unclassified(100);unclassified(100);unclassified(100);
Otu140	0	0	5	0	Bacteria(100);Proteobacteria(100);Gammaproteobacteria(100);Enterobacteriales_Enterobacteriaceae(100);Yersinia(100);unclassified(100);unclassified(100);unclassified(100);unclassified(100);unclassified(100);
Otu141	14	33	0	21	Bacteria(100);Firmicutes(100);Bacilli(100);Lactobacillales(100);Leuconostoc(100);Oenococcus(100);unclassified(100);unclassified(100);unclassified(100);unclassified(100);
Otu142	0	1	0	3	Bacteria(100);Firmicutes(100);Clostridia(100);Clostridiales(100);Z623(100);unclassified(100);unclassified(100);unclassified(100);unclassified(100);unclassified(100);
Otu143	2	1	0	2	Bacteria(100);Firmicutes(100);Clostridia(100);Eubacteriaceae(100);Unclassified(100);unclassified(100);unclassified(100);unclassified(100);unclassified(100);unclassified(100);
Otu144	1	1	0	0	Bacteria(100);Chloroflexi(100);Chloroflexi(100);Chloroflexales(100);Chloroflexaceae(100);unclassified(100);unclassified(100);unclassified(100);unclassified(100);unclassified(100);
Otu145	0	0	1	0	Bacteria(100);Firmicutes(100);Bacilli(100);Lactobacillales(100);Lactobacillaceae(100);Lactobacillus_ruminis(100);unclassified(100);unclassified(100);unclassified(100);unclassified(100);
Otu146	0	0	0	1	Bacteria(100);Chloroflexi(100);TK17(100);Unclassified(100);unclassified(100);unclassified(100);unclassified(100);unclassified(100);unclassified(100);unclassified(100);
Otu147	4	0	6	4	Bacteria(100);Proteobacteria(100);Alphaproteobacteria(100);Consistiales(100);Unclassified(100);unclassified(100);unclassified(100);unclassified(100);unclassified(100);unclassified(100);
Otu148	3	0	2	2	Bacteria(100);Firmicutes(100);Clostridia(100);Clostridiales(100);B360(100);unclassified(100);unclassified(100);unclassified(100);unclassified(100);unclassified(100);
Otu149	0	0	4	0	Bacteria(100);Firmicutes(100);Clostridia(100);Clostridiales(100);C21_k11(100);unclassified(100);unclassified(100);unclassified(100);unclassified(100);unclassified(100);
Otu150	0	0	1	0	Bacteria(100);Proteobacteria(100);Alphaproteobacteria(100);NAC1-6(100);unclassified(100);unclassified(100);unclassified(100);unclassified(100);unclassified(100);unclassified(100);
Otu151	4	0	6	6	Bacteria(100);Acidobacteria(100);Solibacteres(100);Solibacteraceae(100);WD205(100);unclassified(100);unclassified(100);unclassified(100);unclassified(100);unclassified(100);
Otu152	3	6	0	2	Bacteria(100);Firmicutes(100);Symbiobacteria(100);OPB54(100);BSV81(100);unclassified(100);unclassified(100);unclassified(100);unclassified(100);unclassified(100);
Otu153	0	1	0	0	Bacteria(100);Firmicutes(100);Clostridia(100);Clostridiales(100);Johnsonella(100);Eubacterium_cf_saburreum_oral_strain_C27KA(100);Unclassified(100);unclassified(100);unclassified(100);unclassified(100);
Otu154	1	5	0	3	Bacteria(100);Chloroflexi(100);Dehalococcoidetes(100);GIF9(100);MB-A2-101(100);Unclassified(100);unclassified(100);unclassified(100);unclassified(100);unclassified(100);
Otu155	0	0	7	0	Bacteria(100);WS3(100);Sediment-1(100);Unclassified(100);unclassified(100);unclassified(100);unclassified(100);unclassified(100);unclassified(100);unclassified(100);
Otu156	0	0	3	0	Bacteria(100);Firmicutes(100);Clostridia(100);Clostridiales(100);RF30(100);RF6(100);M366(100);unclassified(100);unclassified(100);unclassified(100);
Otu157	2	0	0	0	Bacteria(100);Spirochaetes(100);Treponemaceae(100);Treponema(100);Rs-D01(100);unclassified(100);unclassified(100);unclassified(100);unclassified(100);unclassified(100);
Otu158	0	1	1	1	Bacteria(100);Firmicutes(100);Clostridia(100);p-2531(100);Unclassified(100);unclassified(100);unclassified(100);unclassified(100);unclassified(100);unclassified(100);
Otu159	0	1	0	0	Bacteria(100);OP8(100);OP8_2(100);unclassified(100);unclassified(100);unclassified(100);unclassified(100);unclassified(100);unclassified(100);unclassified(100);
Otu160	0	1	1	0	Bacteria(100);Planctomycetes(100);Kueneniaceae(100);Unclassified(100);unclassified(100);unclassified(100);unclassified(100);unclassified(100);unclassified(100);unclassified(100);
Otu161	0	0	6	1	Bacteria(100);Proteobacteria(100);Gammaproteobacteria(100);Thiomicrospira(100);Thiomicrospiraceae(100);unclassified(100);unclassified(100);unclassified(100);unclassified(100);unclassified(100);
Otu162	2	3	5	5	Bacteria(100);Bacteroidetes(100);Saprospirales(100);Saprospiraceae(100);Saprospira(100);Unclassified(100);unclassified(100);unclassified(100);unclassified(100);unclassified(100);
Otu163	0	0	19	1	Bacteria(100);Proteobacteria(100);Gammaproteobacteria(100);Thiomicrospira(100);Thiomicrospira_frisia(100);Thiomicrospira_chilensis(100);unclassified(100);unclassified(100);unclassified(100);unclassified(100);
Otu164	0	0	1	0	Bacteria(100);Proteobacteria(100);Alphaproteobacteria(100);Rhodobacterales(100);Rhodobacter(100);OM42(100);unclassified(100);unclassified(100);unclassified(100);unclassified(100);
Otu165	0	0	8	1	Bacteria(100);Bacteroidetes(100);SM1A07(100);unclassified(100);unclassified(100);unclassified(100);unclassified(100);unclassified(100);unclassified(100);unclassified(100);
Otu166	0	0	3	53	Bacteria(100);Acidobacteria(100);Chloracidobacteria(100);Ellin6075(100);unclassified(100);unclassified(100);unclassified(100);unclassified(100);unclassified(100);unclassified(100);
Otu167	0	0	0	1	Bacteria(100);Firmicutes(100);Clostridia(100);Clostridiales(100);Clostridiaceae(100);Caloramator(100);Unclassified(100);unclassified(100);unclassified(100);unclassified(100);
Otu168	0	0	0	3	Bacteria(100);Proteobacteria(100);Alphaproteobacteria(100);Brucellaceae(100);unclassified(100);unclassified(100);unclassified(100);unclassified(100);unclassified(100);unclassified(100);
Otu169	0	0	1	0	Bacteria(100);Firmicutes(100);Clostridia(100);Clostridiales(100);Anaerostipes(100);unclassified(100);unclassified(100);unclassified(100);unclassified(100);unclassified(100);
Otu170	0	0	1	3	Bacteria(100);Proteobacteria(100);Gammaproteobacteria(100);Betaproteobacteria(100);Comamonadaceae(100);Delftia(100);unclassified(100);unclassified(100);unclassified(100);unclassified(100);
Otu171	1	0	1	2	Bacteria(100);Firmicutes(100);Clostridia(100);Peptostreptococcaceae(100);Soehngenia(100);unclassified(100);unclassified(100);unclassified(100);unclassified(100);unclassified(100);
Otu172	0	0	5	0	Bacteria(100);Firmicutes(100);Mollicutes(100);Acholeplasmatales(100);Candidatus_Phytoplasma(100);Candidatus_Phytoplasma_oryzae(100);Candidatus_Phytoplasma_fraxini(100);unclassified(100);unclassified(100);unclassified(100);
Otu173	0	2	1	3	Bacteria(100);Proteobacteria(100);Alphaproteobacteria(100);Acetobacterales(100);Roseococcaceae(100);Unclassified(100);unclassified(100);unclassified(100);unclassified(100);unclassified(100);
Otu174	0	0	0	15	Bacteria(100);Proteobacteria(100);Gammaproteobacteria(100);Betaproteobacteria(100);Alcaligenaceae(100);Unclassified(100);unclassified(100);unclassified(100);unclassified(100);unclassified(100);
Otu175	4	0	0	1	Bacteria(100);Firmicutes(100);Clostridia(100);Clostridiales(100);B190(100);unclassified(100);unclassified(100);unclassified(100);unclassified(100);unclassified(100);
Otu176	2	1	1	2	Bacteria(100);Proteobacteria(100);Alphaproteobacteria(100);pLW-38(100);unclassified(100);unclassified(100);unclassified(100);unclassified(100);unclassified(100);unclassified(100);
Otu177	0	1	7	0	Bacteria(100);Chloroflexi(100);Anaerolineae(100);DRC31(100);Napoli-2B-44__BC07-2B-44(100);unclassified(100);unclassified(100);unclassified(100);unclassified(100);unclassified(100);
Otu178	0	0	1	0	Bacteria(100);Planctomycetes(100);Kueneniaceae(100);Brocadia(100);Candidatus_Anammoxoglobus(100);Unclassified(100);unclassified(100);unclassified(100);unclassified(100);unclassified(100);
Otu179	0	1	1	0	Bacteria(100);Firmicutes(100);Clostridia(100);Clostridiales(100);Clostridiaceae(100);Clostridium_pasteurianum(100);unclassified(100);unclassified(100);unclassified(100);unclassified(100);
Otu180	0	0	31	0	Bacteria(100);Proteobacteria(100);Gammaproteobacteria(100);Methylococcaceae(100);Hyd24-01(100);unclassified(100);unclassified(100);unclassified(100);unclassified(100);unclassified(100);
Otu181	0	4	0	0	Bacteria(100);Firmicutes(100);Clostridia(100);Clostridiales(100);Ruminococcus(100);Unclassified(100);unclassified(100);unclassified(100);unclassified(100);unclassified(100);
Otu182	0	0	0	3	Bacteria(100);Proteobacteria(100);Gammaproteobacteria(100);Legionellales(100);Legionella(100);unclassified(100);unclassified(100);unclassified(100);unclassified(100);unclassified(100);
Otu183	0	0	2	0	Bacteria(100);Cyanobacteria(100);Synechococcus(100);Unclassified(100);unclassified(100);unclassified(100);unclassified(100);unclassified(100);unclassified(100);unclassified(100);
Otu184	1	1	4	1	Bacteria(100);Actinobacteria(100);Coriobacteridae(100);Coriobacteriales(100);Collinsella(100);Collinsella_aerofaciens(100);Unclassified(100);unclassified(100);unclassified(100);unclassified(100);
Otu185	0	0	0	3	Bacteria(100);Proteobacteria(100);Gammaproteobacteria(100);Xanthomonadales(100);Lysobacter(100);Unclassified(100);unclassified(100);unclassified(100);unclassified(100);unclassified(100);
Otu186	0	0	1	0	Bacteria(100);Bacteroidetes(100);Bacteroidales(100);p-2534-18B5(100);BE24(100);GRC135(100);unclassified(100);unclassified(100);unclassified(100);unclassified(100);
Otu187	0	0	5	3	Bacteria(100);Bacteroidetes(100);b-17BO(100);unclassified(100);unclassified(100);unclassified(100);unclassified(100);unclassified(100);unclassified(100);unclassified(100);
Otu188	1	0	1	0	Bacteria(100);Firmicutes(100);Mollicutes(100);Spiroplasma_citri(100);Unclassified(100);unclassified(100);unclassified(100);unclassified(100);unclassified(100);unclassified(100);
Otu189	0	0	1	0	Bacteria(100);Firmicutes(100);Clostridia(100);Clostridiales(100);BB68(100);adhufec295(100);unclassified(100);unclassified(100);unclassified(100);unclassified(100);
Otu190	0	0	4	0	Bacteria(100);Proteobacteria(100);Gammaproteobacteria(100);Xanthomonadales(100);gamma_proteobacterium_WJ2(100);unclassified(100);unclassified(100);unclassified(100);unclassified(100);unclassified(100);
Otu191	1	0	0	0	Bacteria(100);Thermotogae(100);MS9(100);unclassified(100);unclassified(100);unclassified(100);unclassified(100);unclassified(100);unclassified(100);unclassified(100);
Otu192	0	0	2	1	Bacteria(100);Proteobacteria(100);Epsilonproteobacteria(100);Sulfuricurvales(100);Sulfuricurvaceae(100);hydrothermal_vent_eubacterium_PVB_OTU_6(100);Unclassified(100);unclassified(100);unclassified(100);unclassified(100);
Otu193	0	0	2	0	Bacteria(100);OP3(100);BD4-9(100);unclassified(100);unclassified(100);unclassified(100);unclassified(100);unclassified(100);unclassified(100);unclassified(100);
Otu194	0	0	0	1	Bacteria(100);Proteobacteria(100);Gammaproteobacteria(100);Endozoicimonas(100);Unclassified(100);unclassified(100);unclassified(100);unclassified(100);unclassified(100);unclassified(100);
Otu195	0	0	0	1	Bacteria(100);Proteobacteria(100);Alphaproteobacteria(100);Crabtreella(100);unclassified(100);unclassified(100);unclassified(100);unclassified(100);unclassified(100);unclassified(100);
Otu196	0	0	0	10	Bacteria(100);Actinobacteria(100);Acidimicrobidae(100);CL500-29(100);ML316M-15(100);unclassified(100);unclassified(100);unclassified(100);unclassified(100);unclassified(100);
Otu197	0	0	0	4	Bacteria(100);Actinobacteria(100);Actinobacteridae(100);Propionibacterineae(100);Unclassified(100);unclassified(100);unclassified(100);unclassified(100);unclassified(100);unclassified(100);
Otu198	0	0	0	1	Bacteria(100);Firmicutes(100);Bacilli(100);TP137(100);unclassified(100);unclassified(100);unclassified(100);unclassified(100);unclassified(100);unclassified(100);

OTU	Number of reads				Greengenes taxonomic classification			
	Wol-	Wol+	DAM	WAL				
Otu199	0	0	0	1	Bacteria(100);Proteobacteria(100);Gammaproteobacteria(100);Betaproteobacteria(100);Comamonadaceae(100);Variovorax_paradoxus(100);unclassified(100);unclassified(100);unclassified(100);			
Otu200	1	0	0	0	Bacteria(100);Firmicutes(100);Bacilli(100);Lactobacillales(100);Carnobacteriaceae(100);Unclassified(100);unclassified(100);unclassified(100);unclassified(100);unclassified(100);			
Otu201	0	1	7	0	Bacteria(100);Firmicutes(100);Peptococcaceae(100);unclassified(100);unclassified(100);unclassified(100);unclassified(100);unclassified(100);unclassified(100);unclassified(100);			
Otu202	0	0	4	1	Bacteria(100);Bacteroidetes(100);Bacteroidales(100);vadinBC27(100);unclassified(100);unclassified(100);unclassified(100);unclassified(100);unclassified(100);unclassified(100);			
Otu203	0	0	0	1	Bacteria(100);Planctomycetes(100);Planctomycetacia(100);Pirellulaceae(100);unclassified(100);unclassified(100);unclassified(100);unclassified(100);unclassified(100);unclassified(100);			
Otu204	0	0	4	1	Bacteria(100);Verrucomicrobia(100);Verruco-5(100);RFP12(100);Unclassified(100);unclassified(100);unclassified(100);unclassified(100);unclassified(100);unclassified(100);			
Otu205	1	0	0	0	Bacteria(100);Proteobacteria(100);Alphaproteobacteria(100);JP57(100);unclassified(100);unclassified(100);unclassified(100);unclassified(100);unclassified(100);unclassified(100);			
Otu206	0	0	0	1	Bacteria(100);Proteobacteria(100);Alphaproteobacteria(100);Bradyrhizobiales(100);Methylocystis_echinoides(100);unclassified(100);unclassified(100);unclassified(100);unclassified(100);unclassified(100);			
Otu207	0	0	1	1	Bacteria(100);Proteobacteria(100);Alphaproteobacteria(100);Anderseniella(100);unclassified(100);unclassified(100);unclassified(100);unclassified(100);unclassified(100);unclassified(100);			
Otu208	0	0	0	1	Bacteria(100);Proteobacteria(100);Alphaproteobacteria(100);Bradyrhizobiales(100);UNNAMEABLE_1185325876(100);unclassified(100);unclassified(100);unclassified(100);unclassified(100);unclassified(100);			
Otu209	0	0	1	1	Bacteria(100);Proteobacteria(100);Gammaproteobacteria(100);Aeromonadaceae(100);Unclassified(100);unclassified(100);unclassified(100);unclassified(100);unclassified(100);unclassified(100);			
Otu210	0	0	2	0	Bacteria(100);Proteobacteria(100);Desulfurellales(100);Unclassified(100);unclassified(100);unclassified(100);unclassified(100);unclassified(100);unclassified(100);unclassified(100);			
Otu211	0	0	7	0	Bacteria(100);Proteobacteria(100);Gammaproteobacteria(100);Enterobacteriales_Enterobacteriaceae(100);Morganella(100);unclassified(100);unclassified(100);unclassified(100);unclassified(100);unclassified(100);			
Otu212	0	0	7	0	Bacteria(100);Firmicutes(100);Clostridia(100);LQ86(100);unclassified(100);unclassified(100);unclassified(100);unclassified(100);unclassified(100);unclassified(100);			
Otu213	0	1	1	0	Bacteria(100);Firmicutes(100);Desulfotomaculum(100);Desulfotomaculum_thermocisternum(100);Desulfotomaculum_thermoacetoxidans(100);unclassified(100);unclassified(100);unclassified(100);unclassified(100);unclassified(100);			
Otu214	0	0	1	0	Bacteria(100);Proteobacteria(100);Alphaproteobacteria(100);Phyllobacteriaceae(100);Phyllobacterium(100);unclassified(100);unclassified(100);unclassified(100);unclassified(100);unclassified(100);			
Otu215	0	0	3	1	Bacteria(100);Spirochaetes(100);Unclassified(100);unclassified(100);unclassified(100);unclassified(100);unclassified(100);unclassified(100);unclassified(100);unclassified(100);			
Otu216	1	1	1	2	Bacteria(100);Firmicutes(100);Mollicutes(100);Mycoplasmatales(100);Mycoplasma_haemomuris(100);BCf7-05(100);Unclassified(100);unclassified(100);unclassified(100);unclassified(100);			
Otu217	0	1	1	0	Bacteria(100);Firmicutes(100);Anaerobrancaceae(100);Dethiobacter(100);unclassified(100);unclassified(100);unclassified(100);unclassified(100);unclassified(100);unclassified(100);			
Otu218	0	0	1	0	Bacteria(100);Thermi(100);Thermales(100);Meiothermaceae(100);Meiothermus(100);Unclassified(100);unclassified(100);unclassified(100);unclassified(100);unclassified(100);			
Otu219	0	0	0	2	Bacteria(100);ABY1_OD1(100);FW129(100);KNA6-NB29(100);unclassified(100);unclassified(100);unclassified(100);unclassified(100);unclassified(100);unclassified(100);			
Otu220	0	0	0	6	Bacteria(100);Bacteroidetes(100);Flexibacterales(100);Dyadobacter(100);unclassified(100);unclassified(100);unclassified(100);unclassified(100);unclassified(100);unclassified(100);			
Otu221	0	0	2	1	Bacteria(100);Firmicutes(100);Mollicutes(100);Clostridium_aff_innocuum_CM970(100);Erysipelothrix(100);p-75-a5(100);unclassified(100);unclassified(100);unclassified(100);unclassified(100);			
Otu222	0	1	1	0	Bacteria(100);Planctomycetes(100);Planctomycetacia(100);Isosphaerales(100);Isosphaeraceae(100);Nostocoida_limicola_III(100);aaa36b05(100);unclassified(100);unclassified(100);unclassified(100);			
Otu223	0	0	0	1	Bacteria(100);Proteobacteria(100);Alphaproteobacteria(100);H4(100);Unclassified(100);unclassified(100);unclassified(100);unclassified(100);unclassified(100);unclassified(100);			
Otu224	0	0	5	1	Bacteria(100);Firmicutes(100);Clostridia(100);Clostridiales(100);Clostridiaceae(100);Clostridium_perfringens(100);unclassified(100);unclassified(100);unclassified(100);unclassified(100);			
Otu225	0	0	0	1	Bacteria(100);Firmicutes(100);Bacilli(100);Lactobacillales(100);Lactobacillaceae(100);Lactobacillus(100);Lactobacillus_delbrueckii(100);p-2370-55G5(100);unclassified(100);unclassified(100);			
Otu226	0	0	3	0	Bacteria(100);Proteobacteria(100);Alphaproteobacteria(100);Bradyrhizobiales(100);Unclassified(100);unclassified(100);unclassified(100);unclassified(100);unclassified(100);unclassified(100);			
Otu227	0	0	1	4	Bacteria(100);Actinobacteria(100);Actinobacteridae(100);Arthrobacter(100);Unclassified(100);unclassified(100);unclassified(100);unclassified(100);unclassified(100);unclassified(100);			
Otu228	0	0	0	2	Bacteria(100);Firmicutes(100);Clostridia(100);Eubacteriaceae(100);Acetobacterium(100);Acetobacterium_woodii(100);unclassified(100);unclassified(100);unclassified(100);unclassified(100);			
Otu229	0	0	6	1	Bacteria(100);Proteobacteria(100);Desulfurellales(100);Desulfurellaceae(100);Unclassified(100);unclassified(100);unclassified(100);unclassified(100);unclassified(100);unclassified(100);			
Otu230	0	0	0	1	Bacteria(100);Gemmatimonadetes(100);Gemm-5(100);Unclassified(100);unclassified(100);unclassified(100);unclassified(100);unclassified(100);unclassified(100);unclassified(100);			
Otu231	0	0	1	0	Bacteria(100);Actinobacteria(100);Actinobacteridae(100);Nocardiaceae(100);unclassified(100);unclassified(100);unclassified(100);unclassified(100);unclassified(100);unclassified(100);			
Otu232	2	0	0	0	Bacteria(100);Chloroflexi(100);RA13C7(100);unclassified(100);unclassified(100);unclassified(100);unclassified(100);unclassified(100);unclassified(100);unclassified(100);			
Otu233	0	0	2	0	Bacteria(100);Proteobacteria(100);Alphaproteobacteria(100);Liberibacter(100);unclassified(100);unclassified(100);unclassified(100);unclassified(100);unclassified(100);unclassified(100);			
Otu234	0	0	3	0	Bacteria(100);Proteobacteria(100);Gammaproteobacteria(100);Betaproteobacteria(100);Neisseriales(100);Chromobacterium(100);unclassified(100);unclassified(100);unclassified(100);unclassified(100);			
Otu235	0	0	4	0	Bacteria(100);Acidobacteria(100);RB25(100);JG37-AG-81(100);unclassified(100);unclassified(100);unclassified(100);unclassified(100);unclassified(100);unclassified(100);			
Otu236	0	0	2	2	Bacteria(100);Actinobacteria(100);Actinobacteridae(100);Leucobacter(100);unclassified(100);unclassified(100);unclassified(100);unclassified(100);unclassified(100);unclassified(100);			
Otu237	0	0	0	2	Bacteria(100);SR1(100);BD2-14(100);unclassified(100);unclassified(100);unclassified(100);unclassified(100);unclassified(100);unclassified(100);unclassified(100);			
Otu238	0	0	0	2	Bacteria(100);Verrucomicrobia(100);Verrucomicrobiae(100);Unclassified(100);unclassified(100);unclassified(100);unclassified(100);unclassified(100);unclassified(100);unclassified(100);			
Otu239	1	0	0	3	Bacteria(100);SR1(100);BH1(100);unclassified(100);unclassified(100);unclassified(100);unclassified(100);unclassified(100);unclassified(100);unclassified(100);			
Otu240	0	0	5	0	Bacteria(100);Proteobacteria(100);Gammaproteobacteria(100);Vibrionaceae(100);Grimontia(100);unclassified(100);unclassified(100);unclassified(100);unclassified(100);unclassified(100);			
Otu241	0	0	0	1	Bacteria(100);Firmicutes(100);Bacilli(100);Unclassified(100);unclassified(100);unclassified(100);unclassified(100);unclassified(100);unclassified(100);unclassified(100);			
Otu242	1	0	0	0	Bacteria(100);Cyanobacteria(100);Chloroplasts(100);vectors(100);Unclassified(100);unclassified(100);unclassified(100);unclassified(100);unclassified(100);unclassified(100);			
Otu243	0	0	1	0	Bacteria(100);Proteobacteria(100);Alphaproteobacteria(100);Consistiales(100);Pelagibacter(100);SAR11(100);Unclassified(100);unclassified(100);unclassified(100);unclassified(100);			
Otu244	0	0	2	1	Bacteria(100);Bacteroidetes(100);Flavobacteriales(100);LCP-72(100);unclassified(100);unclassified(100);unclassified(100);unclassified(100);unclassified(100);unclassified(100);			
Otu245	1	0	0	0	Bacteria(100);Proteobacteria(100);Deltaproteobacteria(100);CD15(100);unclassified(100);unclassified(100);unclassified(100);unclassified(100);unclassified(100);unclassified(100);			
Otu246	0	1	7	2	Bacteria(100);WS5(100);unclassified(100);unclassified(100);unclassified(100);unclassified(100);unclassified(100);unclassified(100);unclassified(100);unclassified(100);			
Otu247	0	0	4	0	Bacteria(100);TM7(100);Unclassified(100);unclassified(100);unclassified(100);unclassified(100);unclassified(100);unclassified(100);unclassified(100);unclassified(100);			
Otu248	0	0	0	1	Bacteria(100);Proteobacteria(100);Alphaproteobacteria(100);Azospirillales(100);Unclassified(100);unclassified(100);unclassified(100);unclassified(100);unclassified(100);unclassified(100);			
Otu249	0	0	0	1	Bacteria(100);Acidobacteria(100);iii1-8(100);32-20(100);unclassified(100);unclassified(100);unclassified(100);unclassified(100);unclassified(100);unclassified(100);			
Otu250	1	0	2	0	Bacteria(100);Spirochaetes(100);Spirochaetales(100);Borreliaceae(100);Unclassified(100);unclassified(100);unclassified(100);unclassified(100);unclassified(100);unclassified(100);			
Otu251	1	0	1	0	Bacteria(100);Proteobacteria(100);Gammaproteobacteria(100);SAR86(100);environmental_sequence(100);CHAB-I-7(100);unclassified(100);unclassified(100);unclassified(100);unclassified(100);			
Otu252	0	0	2	0	Bacteria(100);Bacteroidetes(100);Hymenobacterales(100);Pontibacteraceae(100);Pontibacter(100);unclassified(100);unclassified(100);unclassified(100);unclassified(100);unclassified(100);			
Otu253	0	0	5	1	Bacteria(100);TM7(100);TM7-1(100);K20-27(100);unclassified(100);unclassified(100);unclassified(100);unclassified(100);unclassified(100);unclassified(100);			
Otu254	0	0	1	0	Bacteria(100);Bacteroidetes(100);Bacteroidales(100);Porphyromonadaceae(100);Porphyromonas_canis(100);Unclassified(100);unclassified(100);unclassified(100);unclassified(100);unclassified(100);			
Otu255	0	0	1	16	Bacteria(100);Bacteroidetes(100);Flavobacteriales(100);Flavobacteriaceae(100);Chryseobacterium(100);unclassified(100);unclassified(100);unclassified(100);unclassified(100);unclassified(100);			
Otu256	0	0	0	2	Bacteria(100);Actinobacteria(100);Actinobacteridae(100);Agromyces(100);unclassified(100);unclassified(100);unclassified(100);unclassified(100);unclassified(100);unclassified(100);			
Otu257	0	0	2	8	Bacteria(100);Bacteroidetes(100);Flavobacteriales(100);Flavobacteriaceae(100);Wautersiella(100);unclassified(100);unclassified(100);unclassified(100);unclassified(100);unclassified(100);			
Otu258	0	0	1	0	Bacteria(100);Actinobacteria(100);Actinobacteridae(100);Propionibacterineae(100);Propionibacterium(100);Unclassified(100);unclassified(100);unclassified(100);unclassified(100);unclassified(100);			
Otu259	0	0	0	1	Bacteria(100);Thermoacetogenium(100);unclassified(100);unclassified(100);unclassified(100);unclassified(100);unclassified(100);unclassified(100);unclassified(100);unclassified(100);			
Otu260	0	1	1	1	Bacteria(100);OP8(100);Unclassified(100);unclassified(100);unclassified(100);unclassified(100);unclassified(100);unclassified(100);unclassified(100);unclassified(100);			
Otu261	1	2	1	0	Bacteria(100);Thermodesulfobacteria(100);Unclassified(100);unclassified(100);unclassified(100);unclassified(100);unclassified(100);unclassified(100);unclassified(100);unclassified(100);			
Otu262	0	0	1	0	Bacteria(100);WS6(100);Unclassified(100);unclassified(100);unclassified(100);unclassified(100);unclassified(100);unclassified(100);unclassified(100);unclassified(100);			
Otu263	0	0	1	1	Bacteria(100);Bacteroidetes(100);Saprospirales(100);Terrabacter(100);unclassified(100);unclassified(100);unclassified(100);unclassified(100);unclassified(100);unclassified(100);			
Otu264	1	0	0	0	Bacteria(100);Bacteroidetes(100);Pochenobacter(100);unclassified(100);unclassified(100);unclassified(100);unclassified(100);unclassified(100);unclassified(100);unclassified(100);			

OTU	Number of reads				Greengenes taxonomic classification			
	Wol-	Wol+	DAM	WAL				
Otu265	0	1	5	3	Bacteria(100);Thermodesulfobacteria(100);Thermodesulfatatoraceae(100);Thermodesulfatator(100);unclassified(100);unclassified(100);unclassified(100);unclassified(100);unclassified(100);unclassified(100);			
Otu266	0	0	1	0	Bacteria(100);Bacteroidetes(100);envOPS2(100);unclassified(100);unclassified(100);unclassified(100);unclassified(100);unclassified(100);unclassified(100);unclassified(100);			
Otu267	0	0	0	7	Bacteria(100);BRC1(100);Unclassified(100);unclassified(100);unclassified(100);unclassified(100);unclassified(100);unclassified(100);unclassified(100);unclassified(100);			
Otu268	1	0	0	2	Bacteria(100);Proteobacteria(100);Epsilonproteobacteria(100);Sulfurospirillaceae(100);Sulfurospirillum(100);Unclassified(100);unclassified(100);unclassified(100);unclassified(100);unclassified(100);			
Otu269	0	0	2	0	Bacteria(100);Proteobacteria(100);Gammaproteobacteria(100);Schineria(100);unclassified(100);unclassified(100);unclassified(100);unclassified(100);unclassified(100);unclassified(100);			
Otu270	3	1	4	6	Bacteria(100);Firmicutes(100);Bacilli(100);Lactobacillales(100);Streptococcaceae(100);Streptococcus(100);Unclassified(100);unclassified(100);unclassified(100);unclassified(100);			
Otu271	0	2	0	0	Bacteria(100);Actinobacteria(100);Coriobacteridae(100);Coriobacteriales(100);Collinsella(100);Unclassified(100);unclassified(100);unclassified(100);unclassified(100);unclassified(100);			
Otu272	0	0	1	0	Bacteria(100);Proteobacteria(100);Alphaproteobacteria(100);OM38(100);OCS126(100);Unclassified(100);unclassified(100);unclassified(100);unclassified(100);unclassified(100);			
Otu273	0	5	0	0	Bacteria(100);Firmicutes(100);Clostridia(100);Peptostreptococcaceae(100);Peptostreptococcus_anaerobius(100);Clostridium_bifermentans(100);Unclassified(100);unclassified(100);unclassified(100);unclassified(100);			
Otu274	0	0	1	0	Bacteria(100);Chloroflexi(100);Anaerolineae(100);SJA-101(100);SHA-31(100);unclassified(100);unclassified(100);unclassified(100);unclassified(100);unclassified(100);			
Otu275	0	1	0	0	Bacteria(100);Thermoanaerobacteria(100);Unclassified(100);unclassified(100);unclassified(100);unclassified(100);unclassified(100);unclassified(100);unclassified(100);unclassified(100);			
Otu276	0	0	1	0	Bacteria(100);Planctomycetes(100);vadinHA49(100);unclassified(100);unclassified(100);unclassified(100);unclassified(100);unclassified(100);unclassified(100);unclassified(100);			
Otu277	0	0	1	0	Bacteria(100);Planctomycetes(100);Kueneniaceae(100);Scalindua(100);unclassified(100);unclassified(100);unclassified(100);unclassified(100);unclassified(100);unclassified(100);			
Otu278	0	0	1	1	Bacteria(100);Fusobacteria(100);Ilyobacter(100);unclassified(100);unclassified(100);unclassified(100);unclassified(100);unclassified(100);unclassified(100);unclassified(100);			
Otu279	1	0	3	3	Bacteria(100);Bacteroidetes(100);Bacteroidales(100);Parabacteroidaceae(100);mle1-2(100);unclassified(100);unclassified(100);unclassified(100);unclassified(100);unclassified(100);			
Otu280	0	0	4	0	Bacteria(100);Acidobacteria(100);iii1-15(100);uncultivated_soil_bacterium_clone_C112(100);unclassified(100);unclassified(100);unclassified(100);unclassified(100);unclassified(100);unclassified(100);			
Otu281	0	0	0	1	Bacteria(100);TM7(100);TM7-3(100);CW040(100);unclassified(100);unclassified(100);unclassified(100);unclassified(100);unclassified(100);unclassified(100);			
Otu282	0	0	0	1	Bacteria(100);Proteobacteria(100);Alphaproteobacteria(100);Phyllobacteriaceae(100);Unclassified(100);unclassified(100);unclassified(100);unclassified(100);unclassified(100);unclassified(100);			
Otu283	0	1	1	0	Bacteria(100);Spirochaetes(100);Treponemaceae(100);Treponema(100);U40791(100);unclassified(100);unclassified(100);unclassified(100);unclassified(100);unclassified(100);			
Otu284	0	0	0	1	Bacteria(100);TM7(100);TM7-3(100);Unclassified(100);unclassified(100);unclassified(100);unclassified(100);unclassified(100);unclassified(100);unclassified(100);			
Otu285	0	0	6	0	Bacteria(100);Firmicutes(100);Clostridia(100);Clostridiales(100);Clostridiaceae(100);Clostridium(100);Unclassified(100);unclassified(100);unclassified(100);unclassified(100);			
Otu286	0	0	0	2	Bacteria(100);Proteobacteria(100);Gammaproteobacteria(100);Pseudomonadaceae(100);BRC56(100);unclassified(100);unclassified(100);unclassified(100);unclassified(100);unclassified(100);			
Otu287	0	0	0	1	Bacteria(100);Chloroflexi(100);Anaerolineae(100);Anaerolineales(100);GoM_GC185_546E(100);unclassified(100);unclassified(100);unclassified(100);unclassified(100);unclassified(100);			
Otu288	0	0	1	0	Bacteria(100);Firmicutes(100);Acidaminococcaceae(100);Anaerospora(100);Unclassified(100);unclassified(100);unclassified(100);unclassified(100);unclassified(100);unclassified(100);			
Otu289	0	0	0	1	Bacteria(100);Bacteroidetes(100);Flavobacteriales(100);A714025(100);unclassified(100);unclassified(100);unclassified(100);unclassified(100);unclassified(100);unclassified(100);			
Otu290	0	0	0	1	Bacteria(100);Proteobacteria(100);Alphaproteobacteria(100);Bradyrhizobiales(100);Pleomorphomonas(100);unclassified(100);unclassified(100);unclassified(100);unclassified(100);unclassified(100);			
Otu291	1	0	0	0	Bacteria(100);Firmicutes(100);Bacilli(100);Lactobacillales(100);Leuconostoc(100);Weissella(100);Weissella_kandleri(100);unclassified(100);unclassified(100);unclassified(100);			
Otu292	0	0	1	0	Bacteria(100);Proteobacteria(100);Gammaproteobacteria(100);Betaproteobacteria(100);SBR1001(100);G5(100);unclassified(100);unclassified(100);unclassified(100);unclassified(100);			
Otu293	1	0	1	0	Bacteria(100);Bacteroidetes(100);Persicobacter(100);Persicobacter_diffluens(100);unclassified(100);unclassified(100);unclassified(100);unclassified(100);unclassified(100);unclassified(100);			
Otu294	0	0	1	0	Bacteria(100);Bacteroidetes(100);Flavobacteriales(100);Cytophaga(100);Capnocytophagaceae(100);Capnocytophaga(100);unclassified(100);unclassified(100);unclassified(100);unclassified(100);			
Otu295	0	0	1	1	Bacteria(100);Proteobacteria(100);Deltaproteobacteria(100);Bacteriovorax(100);Bacteriovorax_marinus(100);unclassified(100);unclassified(100);unclassified(100);unclassified(100);unclassified(100);			
Otu296	0	0	0	3	Bacteria(100);Actinobacteria(100);Actinobacteridae(100);Gordoniaceae(100);Mycobacteriaceae(100);unclassified(100);unclassified(100);unclassified(100);unclassified(100);unclassified(100);			
Otu297	0	0	2	0	Bacteria(100);Bacteroidetes(100);Bacteroidales(100);SHA-94(100);unclassified(100);unclassified(100);unclassified(100);unclassified(100);unclassified(100);unclassified(100);			
Otu298	0	0	1	0	Bacteria(100);Firmicutes(100);Bacilli(100);Lactobacillales(100);Streptococcaceae(100);Lactococcus(100);Unclassified(100);unclassified(100);unclassified(100);unclassified(100);			
Otu299	1	0	0	0	Bacteria(100);Firmicutes(100);Bacilli(100);Lactobacillales(100);Streptococcaceae(100);Unclassified(100);unclassified(100);unclassified(100);unclassified(100);unclassified(100);			
Otu300	0	1	0	0	Bacteria(100);Actinobacteria(100);Actinobacteridae(100);Brachybacterium(100);unclassified(100);unclassified(100);unclassified(100);unclassified(100);unclassified(100);unclassified(100);			
Otu301	0	0	1	0	Bacteria(100);Bacteroidetes(100);Bacteroidales(100);Bacteroidaceae(100);B_coprocola(100);unclassified(100);unclassified(100);unclassified(100);unclassified(100);unclassified(100);			
Otu302	0	1	0	0	Bacteria(100);Firmicutes(100);Clostridia(100);Clostridiales(100);Ruminococcus(100);Ruminococcus_bromii(100);Unclassified(100);unclassified(100);unclassified(100);unclassified(100);			
Otu303	0	0	0	3	Bacteria(100);Firmicutes(100);Clostridia(100);Peptostreptococcaceae(100);Unclassified(100);unclassified(100);unclassified(100);unclassified(100);unclassified(100);unclassified(100);			
Otu304	0	0	1	0	Bacteria(100);Firmicutes(100);Bacilli(100);Lactobacillales(100);Unclassified(100);unclassified(100);unclassified(100);unclassified(100);unclassified(100);unclassified(100);			
Otu305	0	0	1	0	Bacteria(100);Actinobacteria(100);Coriobacteridae(100);Coriobacteriales(100);Unclassified(100);unclassified(100);unclassified(100);unclassified(100);unclassified(100);unclassified(100);			
Otu306	0	0	1	0	Bacteria(100);Proteobacteria(100);Gammaproteobacteria(100);ZA2333c(100);Unclassified(100);unclassified(100);unclassified(100);unclassified(100);unclassified(100);unclassified(100);			
Otu307	1	0	0	0	Bacteria(100);Bacteroidetes(100);Crenotrichaceae(100);Rhodothermus(100);unclassified(100);unclassified(100);unclassified(100);unclassified(100);unclassified(100);unclassified(100);			
Otu308	0	0	1	0	Bacteria(100);Bacteroidetes(100);Flavobacteriales(100);Cytophaga(100);Psychroflexus(100);unclassified(100);unclassified(100);unclassified(100);unclassified(100);unclassified(100);			
Otu309	0	0	0	1	Bacteria(100);Firmicutes(100);Bacilli(100);Planococcaceae(100);Kurthia(100);unclassified(100);unclassified(100);unclassified(100);unclassified(100);unclassified(100);			
Otu310	0	0	1	0	Bacteria(100);Proteobacteria(100);Alphaproteobacteria(100);Oleomonas(100);BD7-3(100);unclassified(100);unclassified(100);unclassified(100);unclassified(100);unclassified(100);			
Otu311	0	0	0	1	Bacteria(100);Bacteroidetes(100);Saprospirales(100);Saprospiraceae(100);filamentous_bacterium_Plant1_Iso8(100);unclassified(100);unclassified(100);unclassified(100);unclassified(100);unclassified(100);			
Otu312	1	0	0	0	Bacteria(100);Acidobacteria(100);BPC015(100);unclassified(100);unclassified(100);unclassified(100);unclassified(100);unclassified(100);unclassified(100);unclassified(100);			
Otu313	0	0	2	0	Bacteria(100);Actinobacteria(100);Actinobacteridae(100);Gordoniaceae(100);Corynebacteriaceae(100);Corynebacterium_propinquum(100);unclassified(100);unclassified(100);unclassified(100);unclassified(100);			
Otu314	0	0	0	1	Bacteria(100);Chlorobi(100);BSV19(100);Unclassified(100);unclassified(100);unclassified(100);unclassified(100);unclassified(100);unclassified(100);unclassified(100);			
Otu315	0	0	1	0	Bacteria(100);Proteobacteria(100);Gammaproteobacteria(100);Moraxellaceae(100);Moraxella__Psychrobacter(100);Unclassified(100);unclassified(100);unclassified(100);unclassified(100);unclassified(100);			
Otu316	0	0	1	1	Bacteria(100);Bacteroidetes(100);Saprospirales(100);UNNAMEABLE_1185325920(100);unclassified(100);unclassified(100);unclassified(100);unclassified(100);unclassified(100);unclassified(100);			
Otu317	0	0	0	1	Bacteria(100);Proteobacteria(100);Alphaproteobacteria(100);Kaistia(100);unclassified(100);unclassified(100);unclassified(100);unclassified(100);unclassified(100);unclassified(100);			
Otu318	0	0	0	1	Bacteria(100);Firmicutes(100);Bacilli(100);Planococcaceae(100);Caryophanon(100);unclassified(100);unclassified(100);unclassified(100);unclassified(100);unclassified(100);			
Otu319	0	0	0	1	Bacteria(100);Firmicutes(100);Clostridia(100);Clostridiales(100);Anaerofilum(100);Unclassified(100);unclassified(100);unclassified(100);unclassified(100);unclassified(100);			
Otu320	0	0	1	0	Bacteria(100);WPS-2(100);Unclassified(100);unclassified(100);unclassified(100);unclassified(100);unclassified(100);unclassified(100);unclassified(100);unclassified(100);			
Otu321	0	0	0	1	Bacteria(100);Firmicutes(100);Clostridia(100);Peptostreptococcaceae(100);KSC6-33(100);unclassified(100);unclassified(100);unclassified(100);unclassified(100);unclassified(100);			
Otu322	0	0	1	0	Bacteria(100);Verrucomicrobia(100);Opitutae(100);MB11C04(100);Unclassified(100);unclassified(100);unclassified(100);unclassified(100);unclassified(100);unclassified(100);			
Otu323	0	1	0	0	Bacteria(100);Thermoanaerobacteria(100);Thermoanaerobacter(100);Thermoanaerobacter_mathranii(100);unclassified(100);unclassified(100);unclassified(100);unclassified(100);unclassified(100);unclassified(100);			
Otu324	0	0	1	0	Bacteria(100);Proteobacteria(100);Deltaproteobacteria(100);UA27(100);unclassified(100);unclassified(100);unclassified(100);unclassified(100);unclassified(100);unclassified(100);			
Otu325	0	1	0	0	Bacteria(100);Gemmatimonadetes(100);Gemmatimonadales(100);Gemmatibacter(100);Unclassified(100);unclassified(100);unclassified(100);unclassified(100);unclassified(100);unclassified(100);			
Otu326	1	0	0	0	Bacteria(100);Spirochaetes(100);Treponemaceae(100);Treponema(100);saccharolytic_treponemes(100);Unclassified(100);unclassified(100);unclassified(100);unclassified(100);unclassified(100);			
Otu327	0	0	0	1	Bacteria(100);Planctomycetes(100);agg27(100);OM190(100);ARKCH2Br2-76(100);unclassified(100);unclassified(100);unclassified(100);unclassified(100);unclassified(100);			
