# Supplementary material for: Endosymbiont Dominated Bacterial Communities in a Dwarf Spider
Source: PLoS One. 2015 Feb 23;10(2):e0117297. doi: 10.1371/journal.pone.0117297 (PMC4338242; doi:10.1371/journal.pone.0117297)
Supplement: S5 Information — Taxonomic classification was based on comparison of the most abundant sequence from each cluster against both the type and non-type and cultured and uncultured strain bacterial database using the Ribosomal Database Project (RDP; http://rdp.cme.msu.edu/, accessed 02/07/2013)(Cole et al. 2009). Taxonomy is based on the NCBI nomenclature. OTU’s were determined by phylotype analysis wherein sequences were clustered according to their match with sequences in the SILVA database (as implemented in MOTHUR v 1.29.0) using the Greengenes taxonomic classification. (PDF) [file pone.0117297.s005.pdf]

OTU	Number of reads				Taxonomy (NCBI nomenclature)	RDP classification	S_ab score
	Wol-	Wol+	DAM	WAL			
Otu001	0	0	123	104	uncultured bacterium; 27RHU14; AJ863339	environmental samples:unclassified_environmental samples	1
Otu002	369741	838377	651	771800	uncultured Candidatus Rhabdochlamydia sp.; KF-9; EF445478	Chlamydiae/Verrucomicrobia group:Chlamydiae:Chlamydiia:Chlamydiales:Rhabdochlamydiaceae:Candidatus Rhabdochlamydia	0.833
Otu003	113086	97931	415845	118093	endosymbiont of Brevipalpus obovatus; AY279401	Bacteroidetes/Chlorobi group:Bacteroidetes:unclassified Bacteroidetes:unclassified Bacteroidetes (miscellaneous):unclassified_unclassified Bacteroidetes (miscellaneous)	0.909
Otu004	11	19	303654	79	uncultured Acinetobacter sp.; 91_11; AF467306	Proteobacteria:Gammaproteobacteria:Pseudomonadales:Moraxellaceae:Acinetobacter	1
Otu005	67993	12641	102503	93446	Rickettsia endosymbiont of Asobara tabida; At-R; FJ603467	Proteobacteria:Alphaproteobacteria:Rickettsiales:Rickettsiaceae:Rickettsieae:Rickettsia	1
Otu006	0	2	4	27	uncultured Candidatus Rhabdochlamydia sp.; KF-9; EF445478	Chlamydiae/Verrucomicrobia group:Chlamydiae:Chlamydiia:Chlamydiales:Rhabdochlamydiaceae:Candidatus Rhabdochlamydia	0.442
Otu007	3	64870	166081	92371	Wolbachia sp.; X87407	Proteobacteria:Alphaproteobacteria:Rickettsiales:Anaplasmataceae:Wolbachieae:Wolbachia	1
Otu008	8	438	812	535	Wolbachia symbiont of Icerya purchasi; EU727127	Proteobacteria:Alphaproteobacteria:Rickettsiales:Anaplasmataceae:Wolbachieae:Wolbachia	0.632
Otu009	0	0	1	0	uncultured bacterium; ClL11; FN567051	environmental samples:unclassified_environmental samples	0.316
Otu010	238	459	9	515	uncultured bacterium; GP27685cO3; JN850423	environmental samples:unclassified_environmental samples	0.466
Otu011	364	827	6	786	uncultured Candidatus Rhabdochlamydia sp.; KF-9; EF445478	Chlamydiae/Verrucomicrobia group:Chlamydiae:Chlamydiia:Chlamydiales:Rhabdochlamydiaceae:Candidatus Rhabdochlamydia	0.548
Otu012	0	0	0	4	uncultured bacterium; 16saw137-3e01; GU241561	environmental samples:unclassified_environmental samples	0.813
Otu013	7	1	13	8	Rickettsia endosymbiont of Asobara tabida; At-R; FJ603467	Proteobacteria:Alphaproteobacteria:Rickettsiales:Rickettsiaceae:Rickettsieae:Rickettsia	0.604
Otu014	0	0	1	0	Acinetobacter sp. TDIW13; B05; GU003823	Proteobacteria:Gammaproteobacteria:Pseudomonadales:Moraxellaceae:Acinetobacter	0.423
Otu015	0	2	70	129	Brevundimonas vesicularis; ICS11202; AY456200	Proteobacteria:Alphaproteobacteria:Caulobacterales:Caulobacteraceae:Brevundimonas	1
Otu016	18	61	3	54	uncultured Candidatus Rhabdochlamydia sp.; KF-9; EF445478	Chlamydiae/Verrucomicrobia group:Chlamydiae:Chlamydiia:Chlamydiales:Rhabdochlamydiaceae:Candidatus Rhabdochlamydia	0.404
Otu017	0	0	3	0	Acinetobacter sp. 2P1H11; HF936996	Proteobacteria:Gammaproteobacteria:Pseudomonadales:Moraxellaceae:Acinetobacter	0.545
Otu018	0	0	19	575	uncultured bacterium; 27RHU38; AJ863317	environmental samples:unclassified_environmental samples	0.917
Otu019	0	0	233	0	uncultured gamma proteobacterium; 24HbSt13_AAB; EF534517	Proteobacteria:Gammaproteobacteria:environmental samples:unclassified_environmental samples	0.675
Otu020	77	193	2	190	uncultured Candidatus Rhabdochlamydia sp.; KF-9; EF445478	Chlamydiae/Verrucomicrobia group:Chlamydiae:Chlamydiia:Chlamydiales:Rhabdochlamydiaceae:Candidatus Rhabdochlamydia	0.673
Otu021	0	0	326	0	Acinetobacter haemolyticus (T); DSM 6962; X81662	Proteobacteria:Gammaproteobacteria:Pseudomonadales:Moraxellaceae:Acinetobacter	0.567
Otu022	0	0	5	3	uncultured bacterium; Stegobium_mycetome_symbiont; JQ805029	environmental samples:unclassified_environmental samples	0.619
Otu023	7	10	3	11	uncultured Candidatus Rhabdochlamydia sp.; KF-9; EF445478	Chlamydiae/Verrucomicrobia group:Chlamydiae:Chlamydiia:Chlamydiales:Rhabdochlamydiaceae:Candidatus Rhabdochlamydia	0.545
Otu024	4	1	6	2	uncultured bacterium; Stegobium_mycetome_symbiont; JQ805029	environmental samples:unclassified_environmental samples	0.615
Otu025	68	0	2	533	uncultured bacterium; LM12-2; DQ443901	environmental samples:unclassified_environmental samples	0.795
Otu026	2	1	10	1	uncultured Firmicutes bacterium; CTF3-179; GU958139	Firmicutes:environmental samples:unclassified_environmental samples	0.288
Otu027	1	5	9	4	uncultured Candidatus Rhabdochlamydia sp.; KF-9; EF445478	Chlamydiae/Verrucomicrobia group:Chlamydiae:Chlamydiia:Chlamydiales:Rhabdochlamydiaceae:Candidatus Rhabdochlamydia	0.632
Otu028	0	0	3	2	endosymbiont of Brevipalpus obovatus; AY279401	Bacteroidetes/Chlorobi group:Bacteroidetes:unclassified Bacteroidetes:unclassified Bacteroidetes (miscellaneous):unclassified_unclassified Bacteroidetes (miscellaneous)	0.282
Otu029	6	6	4	6	uncultured Candidatus Rhabdochlamydia sp.; KF-9; EF445478	Chlamydiae/Verrucomicrobia group:Chlamydiae:Chlamydiia:Chlamydiales:Rhabdochlamydiaceae:Candidatus Rhabdochlamydia	0.391
Otu030	0	1	6	3	Rickettsia endosymbiont of Nuclearia pattersoni; AY364636	Proteobacteria:Alphaproteobacteria:Rickettsiales:Rickettsiaceae:Rickettsieae:Rickettsia	0.471
Otu031	114	280	1	293	uncultured Candidatus Rhabdochlamydia sp.; KF-9; EF445478	Chlamydiae/Verrucomicrobia group:Chlamydiae:Chlamydiia:Chlamydiales:Rhabdochlamydiaceae:Candidatus Rhabdochlamydia	0.462
Otu032	0	0	4	1	uncultured bacterium; LaC15L115; EF667561	environmental samples:unclassified_environmental samples	0.458
Otu033	89	10	119	126	uncultured alpha proteobacterium; I-DW-126; GQ453070	Proteobacteria:Alphaproteobacteria:environmental samples:unclassified_environmental samples	0.558
Otu034	0	0	3335	12	uncultured gamma proteobacterium; 24HbSt13_AAB; EF534517	Proteobacteria:Gammaproteobacteria:environmental samples:unclassified_environmental samples	0.917
Otu035	0	0	1	0	uncultured epsilon proteobacterium; pMARB06_10; AB496506	Proteobacteria:delta/epsilon subdivisions:Epsilonproteobacteria:environmental samples:unclassified_environmental samples	0.432
Otu036	3	0	12	6	Rickettsia endosymbiont of Asobara tabida; At-R; FJ603467	Proteobacteria:Alphaproteobacteria:Rickettsiales:Rickettsiaceae:Rickettsieae:Rickettsia	0.561
Otu037	17	7	84	32	Rickettsia endosymbiont of Nuclearia pattersoni; AY364636	Proteobacteria:Alphaproteobacteria:Rickettsiales:Rickettsiaceae:Rickettsieae:Rickettsia	0.497
Otu038	8	13	0	18	uncultured Candidatus Rhabdochlamydia sp.; KF-9; EF445478	Chlamydiae/Verrucomicrobia group:Chlamydiae:Chlamydiia:Chlamydiales:Rhabdochlamydiaceae:Candidatus Rhabdochlamydia	0.442
Otu039	0	2	351	0	uncultured bacterium; R4J7T4_C12; GQ468031	environmental samples:unclassified_environmental samples	0.723
Otu040	0	0	9	0	uncultured Acinetobacter sp.; 91_18; AF467295	Proteobacteria:Gammaproteobacteria:Pseudomonadales:Moraxellaceae:Acinetobacter	0.654
Otu041	0	0	10	0	Acinetobacter sp. 'SMCC B0472'; U87132	Proteobacteria:Gammaproteobacteria:Pseudomonadales:Moraxellaceae:Acinetobacter	0.645
Otu042	0	0	139	1	bacterium UASWS0069; DQ190282	unclassified Bacteria:unclassified Bacteria (miscellaneous):unclassified_unclassified Bacteria (miscellaneous)	0.647
Otu043	0	0	14	0	uncultured gamma proteobacterium; TH1-3; AM690808	Proteobacteria:Gammaproteobacteria:environmental samples:unclassified_environmental samples	0.712
Otu044	2	1	28	5	uncultured alpha proteobacterium; I-DW-126; GQ453070	Proteobacteria:Alphaproteobacteria:environmental samples:unclassified_environmental samples	0.5
Otu045	0	1	1	0	uncultured bacterium; Amsterdam-2B-71; BC20-2B-71; AY592427	environmental samples:unclassified_environmental samples	0.484
Otu046	9	14	8	36	uncultured Candidatus Rhabdochlamydia sp.; KF-9; EF445478	Chlamydiae/Verrucomicrobia group:Chlamydiae:Chlamydiia:Chlamydiales:Rhabdochlamydiaceae:Candidatus Rhabdochlamydia	0.555
Otu047	0	0	3	0	uncultured bacterium; 0702007J19DMCFE10923; GU369404	environmental samples:unclassified_environmental samples	0.59
Otu048	1	0	3	0	uncultured bacterium; MW_62; HQ910898	environmental samples:unclassified_environmental samples	0.39
Otu049	4	4	2	10	uncultured Candidatus Rhabdochlamydia sp.; KF-9; EF445478	Chlamydiae/Verrucomicrobia group:Chlamydiae:Chlamydiia:Chlamydiales:Rhabdochlamydiaceae:Candidatus Rhabdochlamydia	0.622
Otu050	0	0	0	17	uncultured planctomycete; Upland_8_781; JF981504	Planctomycetes:Planctomycetia:Planctomycetales:environmental samples:unclassified_environmental samples	0.812
Otu051	0	0	9	26	uncultured bacterium; 27RHU14; AJ863339	environmental samples:unclassified_environmental samples	0.918
Otu052	0	0	2	1	Rickettsia endosymbiont of Nuclearia pattersoni; AY364636	Proteobacteria:Alphaproteobacteria:Rickettsiales:Rickettsiaceae:Rickettsieae:Rickettsia	0.538
Otu053	2	0	1	3	Rickettsia endosymbiont of Asobara tabida; At-R; FJ603467	Proteobacteria:Alphaproteobacteria:Rickettsiales:Rickettsiaceae:Rickettsieae:Rickettsia	0.5
Otu054	0	0	0	24	uncultured bacterium; e10; AB241562	environmental samples:unclassified_environmental samples	1
Otu055	0	0	64	0	uncultured bacterium; DNA-R3-A2; JN885829	environmental samples:unclassified_environmental samples	0.712
Otu056	0	0	2	0	uncultured bacterium; G910P31FN13.T0; EU172165	environmental samples:unclassified_environmental samples	0.571
Otu057	0	2	0	4	uncultured Candidatus Rhabdochlamydia sp.; KF-9; EF445478	Chlamydiae/Verrucomicrobia group:Chlamydiae:Chlamydiia:Chlamydiales:Rhabdochlamydiaceae:Candidatus Rhabdochlamydia	0.5
Otu058	2	5	0	131	beta proteobacterium endosymbiont of Nesophrosyne sp.; 185n; JX433927	Proteobacteria:Betaproteobacteria:unclassified Betaproteobacteria:unclassified Betaproteobacteria (miscellaneous):unclassified_unclassified Betaproteobacteria (miscellaneous)	0.266
Otu059	0	0	0	47	uncultured bacterium; JJ183; JN004301	environmental samples:unclassified_environmental samples	1
Otu060	1	5	50	124	aquatic bacterium R1-G3; AB195770	unclassified Bacteria:unclassified Bacteria (miscellaneous):unclassified_unclassified Bacteria (miscellaneous)	1
Otu061	6	1	8	9	uncultured alpha proteobacterium; I-DW-126; GQ453070	Proteobacteria:Alphaproteobacteria:environmental samples:unclassified_environmental samples	0.461
Otu062	0	0	0	42	agricultural soil bacterium SI-3; AJ252573	unclassified Bacteria:unclassified Bacteria (miscellaneous):unclassified_unclassified Bacteria (miscellaneous)	1
Otu063	12	1	25	15	Rickettsia endosymbiont of Asobara tabida; At-R; FJ603467	Proteobacteria:Alphaproteobacteria:Rickettsiales:Rickettsiaceae:Rickettsieae:Rickettsia	0.609
Otu064	0	0	14	165	uncultured actinobacterium; SL12a-2/11; GU194255	Actinobacteria:Actinobacteria:environmental samples:unclassified_environmental samples	0.658
Otu065	5	17	1	23	uncultured Candidatus Rhabdochlamydia sp.; KF-9; EF445478	Chlamydiae/Verrucomicrobia group:Chlamydiae:Chlamydiia:Chlamydiales:Rhabdochlamydiaceae:Candidatus Rhabdochlamydia	0.542
Otu066	5	19	27	24	uncultured bacterium; Ob3-7_01; AJ880257	environmental samples:unclassified_environmental samples	0.712
Otu067	1	2	4	5	uncultured bacterium; macon_g23; DQ981015	environmental samples:unclassified_environmental samples	0.451
Otu068	0	0	69	0	bacterium UASWS0069; DQ190282	unclassified Bacteria:unclassified Bacteria (miscellaneous):unclassified_unclassified Bacteria (miscellaneous)	0.692

OTU	Number of reads				Taxonomy (NCBI nomenclature)	RDP classification	S_ab score
	Wol-	Wol+	DAM	WAL			
Otu069	0	0	3	0	uncultured gamma proteobacterium; 24HaSt13_AAB; EF534516	Proteobacteria:Gammaproteobacteria:environmental samples:unclassified_environmental samples	0.718
Otu070	0	0	310	34	uncultured microorganism; SeaGull37; EU181042	unclassified_environmental samples	0.865
Otu071	1	0	0	1	uncultured Candidatus Rhabdochlamydia sp.; KF-9; EF445478	Chlamydiae/Verrucomicrobia group:Chlamydiae:Chlamydiia:Chlamydiales:Rhabdochlamydiaceae:Candidatus Rhabdochlamydia	0.442
Otu072	3	0	13	13	uncultured Clostridia bacterium; WS07B1_G10; DQ171449	Firmicutes:Clostridia:environmental samples:unclassified_environmental samples	0.799
Otu073	3	0	9	30	Stenotrophomonas maltophilia; CC-SB818F1; AY337582	Proteobacteria:Gammaproteobacteria:Xanthomonadales:Xanthomonadaceae:Stenotrophomonas	1
Otu074	0	0	19	3	uncultured Serratia sp.; FC91; FJ608330	Proteobacteria:Gammaproteobacteria:Enterobacteriales:Enterobacteriaceae:Serratia	0.667
Otu075	0	0	0	61	uncultured planctomycete; F155cmL206; JN002808	Planctomycetes:Planctomycetia:Planctomycetales:environmental samples:unclassified_environmental samples	0.753
Otu076	2	1	4	4	uncultured alpha proteobacterium; I-DW-126; GQ453070	Proteobacteria:Alphaproteobacteria:environmental samples:unclassified_environmental samples	0.399
Otu077	0	0	0	41	uncultured Verrucomicrobia bacterium; 39; HQ184346	Chlamydiae/Verrucomicrobia group:Verrucomicrobia:environmental samples:unclassified_environmental samples	0.781
Otu078	0	0	0	23	uncultured Pedobacter sp.; Z2_15; EU560970	Bacteroidetes/Chlorobi group:Bacteroidetes:Sphingobacteriia:Sphingobacteriales:Sphingobacteriaceae:Pedobacter	0.955
Otu079	0	0	2	1	uncultured Firmicutes bacterium; CF2-169; GU958760	Firmicutes:environmental samples:unclassified_environmental samples	0.519
Otu080	0	0	4	0	uncultured bacterium; DNA-R3-A2; JN885829	environmental samples:unclassified_environmental samples	0.615
Otu081	0	1	136	53	uncultured bacterium; 8B-28; FJ719233	environmental samples:unclassified_environmental samples	0.84
Otu082	0	0	28	0	uncultured gamma proteobacterium; 61-01-24c002; DQ316804	Proteobacteria:Gammaproteobacteria: unclassified_environmental samples	0.612
Otu083	0	0	18	18	uncultured bacterium; MD15c6_12662; JQ374518	environmental samples:unclassified_environmental samples	0.949
Otu084	0	4	4	1	uncultured Candidatus Rhabdochlamydia sp.; KF-9; EF445478	Chlamydiae/Verrucomicrobia group:Chlamydiae:Chlamydiia:Chlamydiales:Rhabdochlamydiaceae:Candidatus Rhabdochlamydia	0.387
Otu085	1	2	0	2	Rickettsia sp. 60a; EU430262	Proteobacteria:Alphaproteobacteria:Rickettsiales:Rickettsiaceae:Rickettsieae:Rickettsia	0.394
Otu086	0	0	17	0	uncultured gamma proteobacterium; 357; AB252880	Proteobacteria:Gammaproteobacteria: unclassified_environmental samples	0.462
Otu087	0	0	29	10	uncultured Bradyrhizobiaceae bacterium; Nbac4o25; AY876615	Proteobacteria:Alphaproteobacteria:Rhizobiales:Bradyrhizobiaceae:environmental samples:unclassified_environmental samples	1
Otu088	3	0	1	4	uncultured bacterium; MW_62; HQ910898	environmental samples:unclassified_environmental samples	0.449
Otu089	0	1	0	0	uncultured Candidatus Rhabdochlamydia sp.; KF-9; EF445478	Chlamydiae/Verrucomicrobia group:Chlamydiae:Chlamydiia:Chlamydiales:Rhabdochlamydiaceae:Candidatus Rhabdochlamydia	0.442
Otu090	0	2	2	3	uncultured Candidatus Rhabdochlamydia sp.; KF-9; EF445478	Chlamydiae/Verrucomicrobia group:Chlamydiae:Chlamydiia:Chlamydiales:Rhabdochlamydiaceae:Candidatus Rhabdochlamydia	0.346
Otu091	1	1	1	5	uncultured Clostridiales bacterium; E205B07; HM080425	Firmicutes:Clostridia:Clostridiales:environmental samples:unclassified_environmental samples	0.824
Otu092	0	0	35	2	uncultured bacterium; N3_F3; FN678758	environmental samples:unclassified_environmental samples	0.647
Otu093	0	0	1	0	uncultured bacterium; HW-C15; AB376586	environmental samples:unclassified_environmental samples	0.869
Otu094	1	1	6	1	uncultured Candidatus Rhabdochlamydia sp.; KF-9; EF445478	Chlamydiae/Verrucomicrobia group:Chlamydiae:Chlamydiia:Chlamydiales:Rhabdochlamydiaceae:Candidatus Rhabdochlamydia	0.377
Otu095	0	0	22	1	uncultured bacterium; 65N_G8; FN823912	environmental samples:unclassified_environmental samples	0.609
Otu096	0	0	0	12	bacterium C17; DQ329335	unclassified Bacteria:unclassified Bacteria (miscellaneous):unclassified_unclassified Bacteria (miscellaneous)	0.909
Otu097	1	0	0	0	uncultured bacterium; unfedNYMPH62; HM010906	environmental samples:unclassified_environmental samples	0.647
Otu098	0	0	2	0	uncultured bacterium; BR18-77; AB589871	environmental samples:unclassified_environmental samples	0.532
Otu099	2	0	18	0	uncultured bacterium; ClL11; FN567051	environmental samples:unclassified_environmental samples	0.314
Otu100	1	2	1	6	uncultured Candidatus Rhabdochlamydia sp.; KF-9; EF445478	Chlamydiae/Verrucomicrobia group:Chlamydiae:Chlamydiia:Chlamydiales:Rhabdochlamydiaceae:Candidatus Rhabdochlamydia	0.429
Otu101	0	0	0	15	Arcobacter butzleri; Pig 61; U34386	Proteobacteria:delta/epsilon subdivisions:Epsilonproteobacteria:Campylobacterales:Campylobacteraceae:Arcobacter	1
Otu102	0	1	4	0	uncultured bacterium; N3_F3; FN678758	environmental samples:unclassified_environmental samples	0.618
Otu103	0	0	5	1	uncultured bacterium; ME-21; AB288687	environmental samples:unclassified_environmental samples	0.429
Otu104	5	6	5	11	uncultured Candidatus Rhabdochlamydia sp.; KF-9; EF445478	Chlamydiae/Verrucomicrobia group:Chlamydiae:Chlamydiia:Chlamydiales:Rhabdochlamydiaceae:Candidatus Rhabdochlamydia	0.465
Otu105	0	0	0	41	uncultured bacterium; mMBR.6.21; JQ624229	environmental samples:unclassified_environmental samples	0.747
Otu106	0	0	21	0	uncultured bacterium; 4P_002a_D03; JX672492	environmental samples:unclassified_environmental samples	0.581
Otu107	3	9	13	9	uncultured bacterium; Aero2_D03; FJ747041	environmental samples:unclassified_environmental samples	0.558
Otu108	6	0	18	0	Rickettsia endosymbiont of Dermacentor andersoni; 01-147.R16s; AY375425	Proteobacteria:Alphaproteobacteria:Rickettsiales:Rickettsiaceae:Rickettsieae:Rickettsia	0.268
Otu109	0	0	0	6	uncultured Bacteroidetes bacterium; B3-9; JN371406	Bacteroidetes/Chlorobi group:Bacteroidetes:environmental samples:unclassified_environmental samples	0.673
Otu110	0	0	3	0	Kocuria rhizophila; YI-7-KO-1; AB353333	Actinobacteria:Actinobacteria:Actinobacteridae:Actinomycetales:Micrococcineae:Kocuria	1
Otu111	0	0	0	1	uncultured bacterium; M80; EU215256	environmental samples:unclassified_environmental samples	0.987
Otu112	2	1	19	21	uncultured gamma proteobacterium; TH1-3; AM690808	Proteobacteria:Gammaproteobacteria:environmental samples:unclassified_environmental samples	0.455
Otu113	0	0	0	2	uncultured Candidatus Rhabdochlamydia sp.; KF-9; EF445478	Chlamydiae/Verrucomicrobia group:Chlamydiae:Chlamydiia:Chlamydiales:Rhabdochlamydiaceae:Candidatus Rhabdochlamydia	0.568
Otu114	1	0	1	0	uncultured bacterium; ABRB42; HQ224830	environmental samples:unclassified_environmental samples	0.718
Otu115	0	2	2	1	proteobacterium enrichment culture clone CTBE CDB1009F08; JN817882	Proteobacteria:environmental samples:unclassified_environmental samples	0.321
Otu116	3	5	2	2	uncultured bacterium; Stegobium_mycetome_symbiont; JQ805029	environmental samples:unclassified_environmental samples	0.584
Otu117	0	2	3	0	Rickettsia endosymbiont of Asobara tabida; At-R; FJ603467	Proteobacteria:Alphaproteobacteria:Rickettsiales:Rickettsiaceae:Rickettsieae:Rickettsia	0.487
Otu118	2	0	4	1	uncultured bacterium; 34MIC055; JF341300	environmental samples:unclassified_environmental samples	0.535
Otu119	0	0	3	1	endosymbiont of Brevipalpus obovatus; AY279401	Bacteroidetes/Chlorobi group:Bacteroidetes:unclassified Bacteroidetes:unclassified Bacteroidetes (miscellaneous):unclassified_unclassified Bacteroidetes (miscellaneous)	0.425
Otu120	0	0	1	0	Acinetobacter sp. Cantas3; JN609532	Proteobacteria:Gammaproteobacteria:Pseudomonadales:Moraxellaceae:Acinetobacter	0.371
Otu121	0	0	2	3	uncultured bacterium; 27RHU38; AJ863317	environmental samples:unclassified_environmental samples	0.556
Otu122	0	0	0	1	uncultured Firmicutes bacterium; L2d22UD; HM105337	Firmicutes:environmental samples:unclassified_environmental samples	0.513
Otu123	0	0	1	0	bacterium UASWS0125; DQ190338	unclassified Bacteria:unclassified Bacteria (miscellaneous):unclassified_unclassified Bacteria (miscellaneous)	0.647
Otu124	0	0	35	0	uncultured Acinetobacter sp.; GI6-2-A08; FJ193040	Proteobacteria:Gammaproteobacteria:Pseudomonadales:Moraxellaceae:Acinetobacter	0.654
Otu125	4	6	2	9	uncultured Candidatus Rhabdochlamydia sp.; KF-9; EF445478	Chlamydiae/Verrucomicrobia group:Chlamydiae:Chlamydiia:Chlamydiales:Rhabdochlamydiaceae:Candidatus Rhabdochlamydia	0.548
Otu126	0	0	2	0	human oral bacterium C70; AF202003	unclassified Bacteria:unclassified Bacteria (miscellaneous):unclassified_unclassified Bacteria (miscellaneous)	1
Otu127	2	2	11	1	uncultured bacterium; Napoli-4B-25; BC07-4B-25; AY592763	environmental samples:unclassified_environmental samples	1
Otu128	0	0	0	1	actinobacterium X19; EF634298	Actinobacteria:Actinobacteria:unclassified Actinobacteria:unclassified Actinobacteria (miscellaneous):unclassified_unclassified Actinobacteria (miscellaneous)	0.834
Otu129	0	0	2	1	uncultured bacterium; TG_BD0.2_WB003; HM483721	environmental samples:unclassified_environmental samples	0.627
Otu130	0	0	3	0	uncultured Acinetobacter sp.; 155; EU723430	Proteobacteria:Gammaproteobacteria:Pseudomonadales:Moraxellaceae:Acinetobacter	0.641
Otu131	1	0	1	0	uncultured alpha proteobacterium; I-DW-126; GQ453070	Proteobacteria:Alphaproteobacteria:environmental samples:unclassified_environmental samples	0.622
Otu132	0	1	2	0	uncultured gamma proteobacterium; TH1-3; AM690808	Proteobacteria:Gammaproteobacteria:environmental samples:unclassified_environmental samples	0.675
Otu133	0	1	2	1	uncultured Candidatus Rhabdochlamydia sp.; KF-9; EF445478	Chlamydiae/Verrucomicrobia group:Chlamydiae:Chlamydiia:Chlamydiales:Rhabdochlamydiaceae:Candidatus Rhabdochlamydia	0.667
Otu134	1	2	0	2	uncultured Candidatus Rhabdochlamydia sp.; KF-9; EF445478	Chlamydiae/Verrucomicrobia group:Chlamydiae:Chlamydiia:Chlamydiales:Rhabdochlamydiaceae:Candidatus Rhabdochlamydia	0.448
Otu135	0	0	3	0	Acinetobacter sp. Cantas4; JN609533	Proteobacteria:Gammaproteobacteria:Pseudomonadales:Moraxellaceae:Acinetobacter	0.59
Otu136	0	0	0	2	uncultured planctomycete; Upland_8_781; JF981504	Planctomycetes:Planctomycetia:Planctomycetales:environmental samples:unclassified_environmental samples	0.682

OTU	Number of reads				Taxonomy (NCBI nomenclature)	RDP classification	S_ab score
	Wol-	Wol+	DAM	WAL			
Otu137	0	0	33	0	Acinetobacter sp. IR-811; GU586302	Proteobacteria:Gammaproteobacteria:Pseudomonadales:Moraxellaceae:Acinetobacter	0.66
Otu138	0	0	34	0	bacterium UASWS0069; DQ190282	unclassified Bacteria:unclassified Bacteria (miscellaneous):unclassified_unclassified Bacteria (miscellaneous)	0.647
Otu139	0	0	1	1	Rickettsia endosymbiont of Asobara tabida; At-R; FJ603467	Proteobacteria:Alphaproteobacteria:Rickettsiales:Rickettsiaceae:Rickettsieae:Rickettsia	0.59
Otu140	0	0	5	0	uncultured bacterium; TLSrainConAD02; EU136742	environmental samples:unclassified_environmental samples	0.808
Otu141	14	33	0	21	uncultured Candidatus Rhabdochlamydia sp.; KF-9; EF445478	Chlamydiae/Verrucomicrobia group:Chlamydiae:Chlamydiia:Chlamydiales:Rhabdochlamydiaceae:Candidatus Rhabdochlamydia	0.445
Otu142	0	1	0	3	uncultured bacterium; MW_62; HQ910898	environmental samples:unclassified_environmental samples	0.487
Otu143	2	1	0	2	uncultured Candidatus Rhabdochlamydia sp.; KF-9; EF445478	Chlamydiae/Verrucomicrobia group:Chlamydiae:Chlamydiia:Chlamydiales:Rhabdochlamydiaceae:Candidatus Rhabdochlamydia	0.564
Otu144	1	1	0	0	uncultured Candidatus Rhabdochlamydia sp.; KF-9; EF445478	Chlamydiae/Verrucomicrobia group:Chlamydiae:Chlamydiia:Chlamydiales:Rhabdochlamydiaceae:Candidatus Rhabdochlamydia	0.494
Otu145	0	0	1	0	uncultured bacterium; 0702007L01DMCFF1910; GU369407	environmental samples:unclassified_environmental samples	0.551
Otu146	0	0	0	1	uncultured Candidatus Rhabdochlamydia sp.; KF-9; EF445478	Chlamydiae/Verrucomicrobia group:Chlamydiae:Chlamydiia:Chlamydiales:Rhabdochlamydiaceae:Candidatus Rhabdochlamydia	0.378
Otu147	4	0	6	4	Rickettsia endosymbiont of Asobara tabida; At-R; FJ603467	Proteobacteria:Alphaproteobacteria:Rickettsiales:Rickettsiaceae:Rickettsieae:Rickettsia	0.532
Otu148	3	0	2	2	Rickettsia endosymbiont of Asobara tabida; At-R; FJ603467	Proteobacteria:Alphaproteobacteria:Rickettsiales:Rickettsiaceae:Rickettsieae:Rickettsia	0.558
Otu149	0	0	4	0	Rickettsia endosymbiont of Asobara tabida; At-R; FJ603467	Proteobacteria:Alphaproteobacteria:Rickettsiales:Rickettsiaceae:Rickettsieae:Rickettsia	0.503
Otu150	0	0	1	0	uncultured bacterium; Stegobium_mycetome_symbiont; JQ805029	environmental samples:unclassified_environmental samples	0.6
Otu151	4	0	6	6	Rickettsia endosymbiont of Asobara tabida; At-R; FJ603467	Proteobacteria:Alphaproteobacteria:Rickettsiales:Rickettsiaceae:Rickettsieae:Rickettsia	0.595
Otu152	3	6	0	2	uncultured Candidatus Rhabdochlamydia sp.; KF-9; EF445478	Chlamydiae/Verrucomicrobia group:Chlamydiae:Chlamydiia:Chlamydiales:Rhabdochlamydiaceae:Candidatus Rhabdochlamydia	0.695
Otu153	0	1	0	0	Rickettsia endosymbiont of Asobara tabida; At-R; FJ603467	Proteobacteria:Alphaproteobacteria:Rickettsiales:Rickettsiaceae:Rickettsieae:Rickettsia	0.583
Otu154	1	5	0	3	uncultured Candidatus Rhabdochlamydia sp.; KF-9; EF445478	Chlamydiae/Verrucomicrobia group:Chlamydiae:Chlamydiia:Chlamydiales:Rhabdochlamydiaceae:Candidatus Rhabdochlamydia	0.519
Otu155	0	0	7	0	uncultured bacterium; L2T_300; EF551878	environmental samples:unclassified_environmental samples	0.705
Otu156	0	0	3	0	Rickettsia endosymbiont of Asobara tabida; At-R; FJ603467	Proteobacteria:Alphaproteobacteria:Rickettsiales:Rickettsiaceae:Rickettsieae:Rickettsia	0.487
Otu157	2	0	0	0	Rickettsia endosymbiont of Nuclearia pattersoni; AY364636	Proteobacteria:Alphaproteobacteria:Rickettsiales:Rickettsiaceae:Rickettsieae:Rickettsia	0.5
Otu158	0	1	1	1	Rickettsia endosymbiont of Nuclearia pattersoni; AY364636	Proteobacteria:Alphaproteobacteria:Rickettsiales:Rickettsiaceae:Rickettsieae:Rickettsia	0.366
Otu159	0	1	0	0	uncultured Candidatus Rhabdochlamydia sp.; KF-9; EF445478	Chlamydiae/Verrucomicrobia group:Chlamydiae:Chlamydiia:Chlamydiales:Rhabdochlamydiaceae:Candidatus Rhabdochlamydia	0.474
Otu160	0	1	1	0	uncultured bacterium; D1512W_J93; HQ849850	environmental samples:unclassified_environmental samples	0.477
Otu161	0	0	6	1	uncultured Candidatus Rhabdochlamydia sp.; KF-9; EF445478	Chlamydiae/Verrucomicrobia group:Chlamydiae:Chlamydiia:Chlamydiales:Rhabdochlamydiaceae:Candidatus Rhabdochlamydia	0.219
Otu162	2	3	5	5	uncultured Candidatus Rhabdochlamydia sp.; KF-9; EF445478	Chlamydiae/Verrucomicrobia group:Chlamydiae:Chlamydiia:Chlamydiales:Rhabdochlamydiaceae:Candidatus Rhabdochlamydia	0.292
Otu163	0	0	19	1	uncultured bacterium; G910P31FN13.T0; EU172165	environmental samples:unclassified_environmental samples	0.647
Otu164	0	0	1	0	Acinetobacter sp. TD IW 08; FM164635	Proteobacteria:Gammaproteobacteria:Pseudomonadales:Moraxellaceae:Acinetobacter	0.635
Otu165	0	0	8	1	Acinetobacter sp. 2P1H6; HF936991	Proteobacteria:Gammaproteobacteria:Pseudomonadales:Moraxellaceae:Acinetobacter	0.673
Otu166	0	0	3	53	uncultured bacterium; TB003-01; AB196084	environmental samples:unclassified_environmental samples	0.865
Otu167	0	0	0	1	Rickettsia endosymbiont of Asobara tabida; At-R; FJ603467	Proteobacteria:Alphaproteobacteria:Rickettsiales:Rickettsiaceae:Rickettsieae:Rickettsia	0.6
Otu168	0	0	0	3	uncultured Ochrobactrum sp.; Y6; JF522257	Proteobacteria:Alphaproteobacteria:Rhizobiales:Brucellaceae:Ochrobactrum	0.994
Otu169	0	0	1	0	Rickettsia endosymbiont of Asobara tabida; At-R; FJ603467	Proteobacteria:Alphaproteobacteria:Rickettsiales:Rickettsiaceae:Rickettsieae:Rickettsia	0.548
Otu170	0	0	1	3	uncultured bacterium DEEP-3; AF142877	Proteobacteria:Betaproteobacteria:Burkholderiales:Comamonadaceae:Delftia	0.869
Otu171	1	0	1	2	uncultured bacterium; Stegobium_mycetome_symbiont; JQ805029	environmental samples:unclassified_environmental samples	0.561
Otu172	0	0	5	0	Acinetobacter sp. Cantas3; JN609532	Proteobacteria:Gammaproteobacteria:Pseudomonadales:Moraxellaceae:Acinetobacter	0.487
Otu173	0	2	1	3	uncultured Candidatus Rhabdochlamydia sp.; KF-9; EF445478	Chlamydiae/Verrucomicrobia group:Chlamydiae:Chlamydiia:Chlamydiales:Rhabdochlamydiaceae:Candidatus Rhabdochlamydia	0.526
Otu174	0	0	0	15	uncultured bacterium; Car117fc; AF224846	environmental samples:unclassified_environmental samples	1
Otu175	4	0	0	1	Rickettsia endosymbiont of Asobara tabida; At-R; FJ603467	Proteobacteria:Alphaproteobacteria:Rickettsiales:Rickettsiaceae:Rickettsieae:Rickettsia	0.571
Otu176	2	1	1	2	uncultured bacterium; GP27685cO3; JN850423	environmental samples:unclassified_environmental samples	0.423
Otu177	0	1	7	0	uncultured gamma proteobacterium; WS04A_F10; DQ170984	Proteobacteria:Gammaproteobacteria:environmental samples:unclassified_environmental samples	0.423
Otu178	0	0	1	0	Acinetobacter sp. Cantas3; JN609532	Proteobacteria:Gammaproteobacteria:Pseudomonadales:Moraxellaceae:Acinetobacter	0.526
Otu179	0	1	1	0	uncultured Candidatus Rhabdochlamydia sp.; KF-9; EF445478	Chlamydiae/Verrucomicrobia group:Chlamydiae:Chlamydiia:Chlamydiales:Rhabdochlamydiaceae:Candidatus Rhabdochlamydia	0.425
Otu180	0	0	31	0	bacterium UASWS0069; DQ190282	unclassified Bacteria:unclassified Bacteria (miscellaneous):unclassified_unclassified Bacteria (miscellaneous)	0.647
Otu181	0	4	0	0	uncultured bacterium; Adhufec025rbh; AY471698	environmental samples:unclassified_environmental samples	1
Otu182	0	0	0	3	uncultured bacterium; NC24d2_18949; JQ370011	environmental samples:unclassified_environmental samples	0.814
Otu183	0	0	2	0	Rickettsia endosymbiont of Nuclearia pattersoni; AY364636	Proteobacteria:Alphaproteobacteria:Rickettsiales:Rickettsiaceae:Rickettsieae:Rickettsia	0.589
Otu184	1	1	4	1	uncultured Candidatus Rhabdochlamydia sp.; KF-9; EF445478	Chlamydiae/Verrucomicrobia group:Chlamydiae:Chlamydiia:Chlamydiales:Rhabdochlamydiaceae:Candidatus Rhabdochlamydia	0.308
Otu185	0	0	0	3	uncultured bacterium; 137; FJ718853	environmental samples:unclassified_environmental samples	0.878
Otu186	0	0	1	0	uncultured bacterium; 34T1; EF552239	environmental samples:unclassified_environmental samples	0.39
Otu187	0	0	5	3	uncultured Sphingobacteria bacterium; GASP-WB1S2_H09; EF073373	Bacteroidetes/Chlorobi group:Bacteroidetes:Sphingobacteriia:environmental samples:unclassified_environmental samples	0.477
Otu188	1	0	1	0	Rickettsia endosymbiont of Asobara tabida; At-R; FJ603467	Proteobacteria:Alphaproteobacteria:Rickettsiales:Rickettsiaceae:Rickettsieae:Rickettsia	0.609
Otu189	0	0	1	0	uncultured bacterium; Stegobium_mycetome_symbiont; JQ805029	environmental samples:unclassified_environmental samples	0.5
Otu190	0	0	4	0	uncultured bacterium; P38; JQ958723	environmental samples:unclassified_environmental samples	0.59
Otu191	1	0	0	0	uncultured Candidatus Rhabdochlamydia sp.; KF-9; EF445478	Chlamydiae/Verrucomicrobia group:Chlamydiae:Chlamydiia:Chlamydiales:Rhabdochlamydiaceae:Candidatus Rhabdochlamydia	0.462
Otu192	0	0	2	1	uncultured bacterium; Amsterdam-2B-71; BC20-2B-71; AY592427	environmental samples:unclassified_environmental samples	0.506
Otu193	0	0	2	0	endosymbiont of Brevipalpus obovatus; AY279401	Bacteroidetes/Chlorobi group:Bacteroidetes:unclassified Bacteroidetes:unclassified Bacteroidetes (miscellaneous):unclassified_unclassified Bacteroidetes (miscellaneous)	0.516
Otu194	0	0	0	1	Azotobacter tropicalis; AM9; JN591767	Proteobacteria:Gammaproteobacteria:Pseudomonadales:Pseudomonadaceae:Azotobacter group:Azotobacter	0.721
Otu195	0	0	0	1	Rhizobium sp. RCD1; GU902301	Proteobacteria:Alphaproteobacteria:Rhizobiales:Rhizobiaceae:Rhizobium/Agrobacterium group:Rhizobium	0.827
Otu196	0	0	0	10	uncultured actinobacterium; lhac1; DQ648926	Actinobacteria:Actinobacteria:environmental samples:unclassified_environmental samples	0.955
Otu197	0	0	0	4	uncultured bacterium; D21R45C50; FM956835	environmental samples:unclassified_environmental samples	0.855
Otu198	0	0	0	1	bacterium enrichment culture clone MW-8; JX096819	environmental samples:unclassified_environmental samples	0.429
Otu199	0	0	0	1	uncultured Variovorax sp.; GASP-WB1S2_F06; EF073432	Proteobacteria:Betaproteobacteria:Burkholderiales:Comamonadaceae:Variovorax	1
Otu200	1	0	0	0	uncultured bacterium; II-03; GU724742	environmental samples:unclassified_environmental samples	0.962
Otu201	0	1	7	0	uncultured gamma proteobacterium; TH1-3; AM690808	Proteobacteria:Gammaproteobacteria:environmental samples:unclassified_environmental samples	0.583
Otu202	0	0	4	1	uncultured bacterium; 0702007L01DMCFF1910; GU369407	environmental samples:unclassified_environmental samples	0.506
Otu203	0	0	0	1	uncultured bacterium; AR114; GQ860192	environmental samples:unclassified_environmental samples	0.761
Otu204	0	0	4	1	Rickettsia endosymbiont of Asobara tabida; At-R; FJ603467	Proteobacteria:Alphaproteobacteria:Rickettsiales:Rickettsiaceae:Rickettsieae:Rickettsia	0.302

OTU	Number of reads				Taxonomy (NCBI nomenclature)	RDP classification	S_ab score
	Wol-	Wol+	DAM	WAL			
Otu205	1	0	0	0	uncultured Candidatus Rhabdochlamydia sp.; KF-9; EF445478	Chlamydiae/Verrucomicrobia group:Chlamydiae:Chlamydiia:Chlamydiales:Rhabdochlamydiaceae:Candidatus Rhabdochlamydia	0.484
Otu206	0	0	0	1	uncultured proteobacterium; Upland_16_1262; JF981981	Proteobacteria:environmental samples:unclassified_environmental samples	1
Otu207	0	0	1	1	uncultured Candidatus Cardinium sp.; EU333930	Bacteroidetes/Chlorobi group:Bacteroidetes:unclassified Bacteroidetes:Candidatus Cardinium	0.431
Otu208	0	0	0	1	uncultured bacterium; 1494; JN855126	environmental samples:unclassified_environmental samples	0.948
Otu209	0	0	1	1	uncultured bacterium; MW_62; HQ910898	environmental samples:unclassified_environmental samples	0.525
Otu210	0	0	2	0	uncultured bacterium; ClL11; FN567051	environmental samples:unclassified_environmental samples	0.287
Otu211	0	0	7	0	uncultured bacterium; FS1; EU034457	environmental samples:unclassified_environmental samples	0.718
Otu212	0	0	7	0	Acinetobacter sp. 2P1H6; HF936991	Proteobacteria:Gammaproteobacteria:Pseudomonadales:Moraxellaceae:Acinetobacter	0.686
Otu213	0	1	1	0	uncultured bacterium; HFV02_110; GU103752	environmental samples:unclassified_environmental samples	0.5
Otu214	0	0	1	0	uncultured bacterium; 0B8; EU499490	environmental samples:unclassified_environmental samples	0.645
Otu215	0	0	3	1	uncultured bacterium; BR18-77; AB589871	environmental samples:unclassified_environmental samples	0.571
Otu216	1	1	1	2	uncultured Candidatus Rhabdochlamydia sp.; KF-9; EF445478	Chlamydiae/Verrucomicrobia group:Chlamydiae:Chlamydiia:Chlamydiales:Rhabdochlamydiaceae:Candidatus Rhabdochlamydia	0.423
Otu217	0	1	1	0	Candidatus Rickettsia hoogstraalii; Croatica; FJ767735	Proteobacteria:Alphaproteobacteria:Rickettsiales:Rickettsiaceae:Rickettsieae:Rickettsia	0.253
Otu218	0	0	1	0	Rickettsia endosymbiont of Nuclearia pattersoni; AY364636	Proteobacteria:Alphaproteobacteria:Rickettsiales:Rickettsiaceae:Rickettsieae:Rickettsia	0.384
Otu219	0	0	0	2	uncultured bacterium; CadhumucNU2bF05; DQ339856	environmental samples:unclassified_environmental samples	0.227
Otu220	0	0	0	6	Sphingobacteriales bacterium TP373; EF636200	Bacteroidetes/Chlorobi group:Bacteroidetes:Sphingobacteriia:Sphingobacteriales:unclassified Sphingobacteriales:unclassified Sphingobacteriales (miscellaneous)	0.954
Otu221	0	0	2	1	uncultured bacterium; Stegobium_mycetome_symbiont; JQ805029	environmental samples:unclassified_environmental samples	0.31
Otu222	0	1	1	0	uncultured Candidatus Rhabdochlamydia sp.; KF-9; EF445478	Chlamydiae/Verrucomicrobia group:Chlamydiae:Chlamydiia:Chlamydiales:Rhabdochlamydiaceae:Candidatus Rhabdochlamydia	0.397
Otu223	0	0	0	1	uncultured bacterium; McL21; FN567884	environmental samples:unclassified_environmental samples	0.641
Otu224	0	0	5	1	Acinetobacter johnsonii; F27; EF204267	Proteobacteria:Gammaproteobacteria:Pseudomonadales:Moraxellaceae:Acinetobacter	0.647
Otu225	0	0	0	1	uncultured bacterium; XSHA5; AY601720	environmental samples:unclassified_environmental samples	0.955
Otu226	0	0	3	0	uncultured bacterium; DL071; AB241178	environmental samples:unclassified_environmental samples	0.967
Otu227	0	0	1	4	Micrococcus sp. V4.MO.30; V4.MO.30 = MM_2718; AJ244663	Actinobacteria:Actinobacteria:Actinobacteridae:Actinomycetales:Micrococcineae:Micrococcus	1
Otu228	0	0	0	2	uncultured bacterium; clone group A1ghqi; X91424	environmental samples:unclassified_environmental samples	1
Otu229	0	0	6	1	uncultured bacterium; areia_46; GQ996478	environmental samples:unclassified_environmental samples	0.686
Otu230	0	0	0	1	uncultured Candidatus Rhabdochlamydia sp.; KF-9; EF445478	Chlamydiae/Verrucomicrobia group:Chlamydiae:Chlamydiia:Chlamydiales:Rhabdochlamydiaceae:Candidatus Rhabdochlamydia	0.396
Otu231	0	0	1	0	uncultured bacterium; NL49_4; GU816187	environmental samples:unclassified_environmental samples	0.917
Otu232	2	0	0	0	uncultured bacterium; CHPA.0912.78; HQ904470	environmental samples:unclassified_environmental samples	0.5
Otu233	0	0	2	0	uncultured bacterium; B16_B71; GQ458103	environmental samples:unclassified_environmental samples	0.439
Otu234	0	0	3	0	Acinetobacter radioresistens; SS23; JX032809	Proteobacteria:Gammaproteobacteria:Pseudomonadales:Moraxellaceae:Acinetobacter	0.545
Otu235	0	0	4	0	uncultured bacterium; Amsterdam-2B-71; BC20-2B-71; AY592427	environmental samples:unclassified_environmental samples	0.59
Otu236	0	0	2	2	Leucobacter sp. N8; EF590233	Actinobacteria:Actinobacteria:Actinobacteridae:Actinomycetales:Micrococcineae:Leucobacter	0.974
Otu237	0	0	0	2	uncultured bacterium; MW_62; HQ910898	environmental samples:unclassified_environmental samples	0.429
Otu238	0	0	0	2	uncultured Verrucomicrobia bacterium; 100M1_H4; DQ514041	Chlamydiae/Verrucomicrobia group:Verrucomicrobia:environmental samples:unclassified_environmental samples	0.755
Otu239	1	0	0	3	uncultured bacterium; BFF09x_A9A; JQ046457	environmental samples:unclassified_environmental samples	0.506
Otu240	0	0	5	0	uncultured gamma proteobacterium; 24HbSt13_AAB; EF534517	Proteobacteria:Gammaproteobacteria:environmental samples:unclassified_environmental samples	0.59
Otu241	0	0	0	1	uncultured bacterium; FWC12_MS; JQ480743	environmental samples:unclassified_environmental samples	0.865
Otu242	1	0	0	0	uncultured bacterium; s41; AY171306	environmental samples:unclassified_environmental samples	1
Otu243	0	0	1	0	Acinetobacter sp. TDIW13; B05; GU003823	Proteobacteria:Gammaproteobacteria:Pseudomonadales:Moraxellaceae:Acinetobacter	0.622
Otu244	0	0	2	1	Rickettsia sp. 60a; EU430262	Proteobacteria:Alphaproteobacteria:Rickettsiales:Rickettsiaceae:Rickettsieae:Rickettsia	0.333
Otu245	1	0	0	0	uncultured Candidatus Rhabdochlamydia sp.; KF-9; EF445478	Chlamydiae/Verrucomicrobia group:Chlamydiae:Chlamydiia:Chlamydiales:Rhabdochlamydiaceae:Candidatus Rhabdochlamydia	0.477
Otu246	0	1	7	2	uncultured bacterium; MW_62; HQ910898	environmental samples:unclassified_environmental samples	0.397
Otu247	0	0	4	0	Acinetobacter sp. TDIW13; B05; GU003823	Proteobacteria:Gammaproteobacteria:Pseudomonadales:Moraxellaceae:Acinetobacter	0.545
Otu248	0	0	0	1	Rickettsia endosymbiont of Nuclearia pattersoni; AY364636	Proteobacteria:Alphaproteobacteria:Rickettsiales:Rickettsiaceae:Rickettsieae:Rickettsia	0.564
Otu249	0	0	0	1	uncultured Candidatus Rhabdochlamydia sp.; KF-9; EF445478	Chlamydiae/Verrucomicrobia group:Chlamydiae:Chlamydiia:Chlamydiales:Rhabdochlamydiaceae:Candidatus Rhabdochlamydia	0.416
Otu250	1	0	2	0	uncultured Candidatus Rhabdochlamydia sp.; KF-9; EF445478	Chlamydiae/Verrucomicrobia group:Chlamydiae:Chlamydiia:Chlamydiales:Rhabdochlamydiaceae:Candidatus Rhabdochlamydia	0.374
Otu251	1	0	1	0	uncultured bacterium; Stegobium_mycetome_symbiont; JQ805029	environmental samples:unclassified_environmental samples	0.571
Otu252	0	0	2	0	Rickettsia endosymbiont of Nuclearia pattersoni; AY364636	Proteobacteria:Alphaproteobacteria:Rickettsiales:Rickettsiaceae:Rickettsieae:Rickettsia	0.346
Otu253	0	0	5	1	uncultured bacterium; Bul2ac05; FJ228814	environmental samples:unclassified_environmental samples	0.503
Otu254	0	0	1	0	Acinetobacter junii; DQ224385	Proteobacteria:Gammaproteobacteria:Pseudomonadales:Moraxellaceae:Acinetobacter	0.436
Otu255	0	0	1	16	uncultured Bacteroidetes bacterium; SSG2SE06; JN567968	Bacteroidetes/Chlorobi group:Bacteroidetes:environmental samples:unclassified_environmental samples	0.844
Otu256	0	0	0	2	uncultured prokaryote; BSLc63; EF682366	prokaryotic environmental samples:unclassified_prokaryotic environmental samples	1
Otu257	0	0	2	8	Flavobacterium sp. H1; EF590224	Bacteroidetes/Chlorobi group:Bacteroidetes:Flavobacteriia:Flavobacteriales:Flavobacteriaceae:Flavobacterium	1
Otu258	0	0	1	0	Propionibacterium acnes; AF076032	Actinobacteria:Actinobacteria:Actinobacteridae:Actinomycetales:Propionibacterineae:Propionibacterium	0.955
Otu259	0	0	0	1	Rickettsia endosymbiont of Asobara tabida; At-R; FJ603467	Proteobacteria:Alphaproteobacteria:Rickettsiales:Rickettsiaceae:Rickettsieae:Rickettsia	0.374
Otu260	0	1	1	1	Wolbachia pipientis; HQ121414	Proteobacteria:Alphaproteobacteria:Rickettsiales:Anaplasmataceae:Wolbachieae:Wolbachia	0.309
Otu261	1	2	1	0	uncultured Candidatus Rhabdochlamydia sp.; KF-9; EF445478	Chlamydiae/Verrucomicrobia group:Chlamydiae:Chlamydiia:Chlamydiales:Rhabdochlamydiaceae:Candidatus Rhabdochlamydia	0.556
Otu262	0	0	1	0	uncultured bacterium; SHZA469; GQ154753	environmental samples:unclassified_environmental samples	0.426
Otu263	0	0	1	1	Rickettsia endosymbiont of Nuclearia pattersoni; AY364636	Proteobacteria:Alphaproteobacteria:Rickettsiales:Rickettsiaceae:Rickettsieae:Rickettsia	0.283
Otu264	1	0	0	0	Acinetobacter baumannii; QQb; GU300764	Proteobacteria:Gammaproteobacteria:Pseudomonadales:Moraxellaceae:Acinetobacter	0.27
Otu265	0	1	5	3	uncultured bacterium; GP27685cO3; JN850423	environmental samples:unclassified_environmental samples	0.37
Otu266	0	0	1	0	Acinetobacter sp. Cantas3; JN609532	Proteobacteria:Gammaproteobacteria:Pseudomonadales:Moraxellaceae:Acinetobacter	0.643
Otu267	0	0	0	7	uncultured bacterium; Upland_120_7942; JF988642	environmental samples:unclassified_environmental samples	0.942
Otu268	1	0	0	2	uncultured epsilon proteobacterium; LKC2.127-7; AY208812	Proteobacteria:delta/epsilon subdivisions:Epsilonproteobacteria:environmental samples:unclassified_environmental samples	1
Otu269	0	0	2	0	uncultured bacterium; SB-d0-2; JQ664572	environmental samples:unclassified_environmental samples	0.607
Otu270	3	1	4	6	uncultured prokaryote; DGGE band GW1a3-5(S); AY501817	prokaryotic environmental samples:unclassified_prokaryotic environmental samples	1
Otu271	0	2	0	0	uncultured Candidatus Rhabdochlamydia sp.; KF-9; EF445478	Chlamydiae/Verrucomicrobia group:Chlamydiae:Chlamydiia:Chlamydiales:Rhabdochlamydiaceae:Candidatus Rhabdochlamydia	0.439
Otu272	0	0	1	0	uncultured bacterium; BFF09x_A5A; JQ046439	environmental samples:unclassified_environmental samples	0.575

OTU	Number of reads				Taxonomy (NCBI nomenclature)	RDP classification	S_ab score
	Wol-	Wol+	DAM	WAL			
Otu273	0	5	0	0	uncultured bacterium O8SA; AF018039	environmental samples:unclassified_environmental samples	1
Otu274	0	0	1	0	uncultured Moraxellaceae bacterium; PA+C_F02; FJ665941	Proteobacteria:Gammaproteobacteria:Pseudomonadales:Moraxellaceae:environmental samples:unclassified_environmental samples	0.532
Otu275	0	1	0	0	uncultured Candidatus Rhabdochlamydia sp.; KF-9; EF445478	Chlamydiae/Verrucomicrobia group:Chlamydiae:Chlamydiia:Chlamydiales:Rhabdochlamydiaceae:Candidatus Rhabdochlamydia	0.477
Otu276	0	0	1	0	uncultured gamma proteobacterium; BC18; EF619936	Proteobacteria:Gammaproteobacteria:environmental samples:unclassified_environmental samples	0.507
Otu277	0	0	1	0	uncultured bacterium; WHB47-58; AB426380	environmental samples:unclassified_environmental samples	0.474
Otu278	0	0	1	1	Rickettsia endosymbiont of Nuclearia pattersoni; AY364636	Proteobacteria:Alphaproteobacteria:Rickettsiales:Rickettsiaceae:Rickettsieae:Rickettsia	0.346
Otu279	1	0	3	3	uncultured bacterium; DGGE gel band D17_7; DQ906130	environmental samples:unclassified_environmental samples	1
Otu280	0	0	4	0	Acinetobacter sp. st25; FJ544373	Proteobacteria:Gammaproteobacteria:Pseudomonadales:Moraxellaceae:Acinetobacter	0.639
Otu281	0	0	0	1	Rickettsia endosymbiont of Bemisia tabaci; R12; GU563837	Proteobacteria:Alphaproteobacteria:Rickettsiales:Rickettsiaceae:Rickettsieae:Rickettsia	0.481
Otu282	0	0	0	1	uncultured Mesorhizobium sp.; UVmen1_27; JQ701574	Proteobacteria:Alphaproteobacteria:Rhizobiales:Phyllobacteriaceae:Mesorhizobium	0.955
Otu283	0	1	1	0	uncultured bacterium; 3P-3-2-N22; EU705928	environmental samples:unclassified_environmental samples	0.27
Otu284	0	0	0	1	uncultured bacterium; Z4_72; EU560973	environmental samples:unclassified_environmental samples	0.955
Otu285	0	0	6	0	uncultured bacterium; P3IU-35; AF414573	environmental samples:unclassified_environmental samples	0.968
Otu286	0	0	0	2	uncultured bacterium; Nbs_454_243; JX311011	environmental samples:unclassified_environmental samples	0.801
Otu287	0	0	0	1	uncultured Acinetobacter sp.; SS52D120; GU356368	Proteobacteria:Gammaproteobacteria:Pseudomonadales:Moraxellaceae:Acinetobacter	0.447
Otu288	0	0	1	0	endosymbiont of Brevipalpus obovatus; AY279401	Bacteroidetes/Chlorobi group:Bacteroidetes:unclassified Bacteroidetes:unclassified Bacteroidetes (miscellaneous):unclassified_unclassified Bacteroidetes (miscellaneous)	0.421
Otu289	0	0	0	1	uncultured bacterium; Lc2z_ML_107; FJ355257	environmental samples:unclassified_environmental samples	0.529
Otu290	0	0	0	1	uncultured alpha proteobacterium; RT03-HC-41; AY475202	Proteobacteria:Alphaproteobacteria:environmental samples:unclassified_environmental samples	0.622
Otu291	1	0	0	0	uncultured alpha proteobacterium; I-DW-126; GQ453070	Proteobacteria:Alphaproteobacteria:environmental samples:unclassified_environmental samples	0.484
Otu292	0	0	1	0	uncultured bacterium; Stegobium_mycetome_symbiont; JQ805029	environmental samples:unclassified_environmental samples	0.577
Otu293	1	0	1	0	Rickettsia endosymbiont of Asobara tabida; At-R; FJ603467	Proteobacteria:Alphaproteobacteria:Rickettsiales:Rickettsiaceae:Rickettsieae:Rickettsia	0.487
Otu294	0	0	1	0	uncultured bacterium; L2T_300; EF551878	environmental samples:unclassified_environmental samples	0.426
Otu295	0	0	1	1	uncultured Candidatus Rhabdochlamydia sp.; KF-9; EF445478	Chlamydiae/Verrucomicrobia group:Chlamydiae:Chlamydiia:Chlamydiales:Rhabdochlamydiaceae:Candidatus Rhabdochlamydia	0.455
Otu296	0	0	0	3	Mycobacterium sp. GIC5; AY439252	Actinobacteria:Actinobacteria:Actinobacteridae:Actinomycetales:Corynebacterineae:Mycobacterium	1
Otu297	0	0	2	0	uncultured bacterium; 0702007J19DMCFE10923; GU369404	environmental samples:unclassified_environmental samples	0.405
Otu298	0	0	1	0	uncultured bacterium; d1-57; AM409932	environmental samples:unclassified_environmental samples	0.276
Otu299	1	0	0	0	Streptococcus mutans; CECT 4034; AJ554208	Firmicutes:Bacilli:Lactobacillales:Streptococcaceae:Streptococcus	1
Otu300	0	1	0	0	uncultured bacterium; OS-60; AB205991	environmental samples:unclassified_environmental samples	1
Otu301	0	0	1	0	endosymbiont of Metaseiulus occidentalis; AY279413	Bacteroidetes/Chlorobi group:Bacteroidetes:unclassified Bacteroidetes:unclassified Bacteroidetes (miscellaneous):unclassified_unclassified Bacteroidetes (miscellaneous)	0.571
Otu302	0	1	0	0	uncultured bacterium; gs20; JF420626	environmental samples:unclassified_environmental samples	0.417
Otu303	0	0	0	3	Candidatus Peptoniphilus massiliensis; 2; DQ029044	Firmicutes:Clostridia:Clostridiales:Clostridiales incertae sedis:Clostridiales Family XI. Incertae Sedis:Peptoniphilus	1
Otu304	0	0	1	0	uncultured Granulicatella sp.; BL005B21; AY806069	Firmicutes:Bacilli:Lactobacillales:Carnobacteriaceae:Granulicatella	1
Otu305	0	0	1	0	uncultured bacterium; MW_62; HQ910898	environmental samples:unclassified_environmental samples	0.524
Otu306	0	0	1	0	Pseudomonas fluorescens; CHA0r; AJ697956	Proteobacteria:Gammaproteobacteria:Pseudomonadales:Pseudomonadaceae:Pseudomonas	0.81
Otu307	1	0	0	0	uncultured bacterium; MW_62; HQ910898	environmental samples:unclassified_environmental samples	0.591
Otu308	0	0	1	0	uncultured bacterium; G910P31FN13.T0; EU172165	environmental samples:unclassified_environmental samples	0.645
Otu309	0	0	0	1	Lysinibacillus sp. SS-2009-PA8; FN646609	Firmicutes:Bacilli:Bacillales:Bacillaceae:Lysinibacillus	0.774
Otu310	0	0	1	0	uncultured bacterium; G910P31FN13.T0; EU172165	environmental samples:unclassified_environmental samples	0.519
Otu311	0	0	0	1	uncultured organism; SBZA_618; JN498434	unclassified_environmental samples	0.429
Otu312	1	0	0	0	uncultured Candidatus Rhabdochlamydia sp.; KF-9; EF445478	Chlamydiae/Verrucomicrobia group:Chlamydiae:Chlamydiia:Chlamydiales:Rhabdochlamydiaceae:Candidatus Rhabdochlamydia	0.504
Otu313	0	0	2	0	Acinetobacter radioresistens; NW3-11; AY568483	Proteobacteria:Gammaproteobacteria:Pseudomonadales:Moraxellaceae:Acinetobacter	0.57
Otu314	0	0	0	1	uncultured bacterium; UOXD-d04; EU869771	environmental samples:unclassified_environmental samples	0.378
Otu315	0	0	1	0	gamma proteobacterium S-St(0)-7B; AB074738	Proteobacteria:Gammaproteobacteria:unclassified Gammaproteobacteria:unclassified Gammaproteobacteria (miscellaneous):unclassified_unclassified Gammaproteobacteria (miscellaneo	1
Otu316	0	0	1	1	uncultured Desulfocapsa sp.; BFB101; KC545769	Proteobacteria:delta/epsilon subdivisions:Deltaproteobacteria:Desulfobacterales:Desulfobulbaceae:Desulfocapsa	0.275
Otu317	0	0	0	1	uncultured bacterium; T1C; KC352402	environmental samples:unclassified_environmental samples	0.76
Otu318	0	0	0	1	Cardinium endosymbiont of Pseudoparlatoria sp.; GQ455416	Bacteroidetes/Chlorobi group:Bacteroidetes:unclassified Bacteroidetes:Candidatus Cardinium	0.457
Otu319	0	0	0	1	uncultured bacterium; Eldhufec321; AY920196	environmental samples:unclassified_environmental samples	0.296
Otu320	0	0	1	0	Acinetobacter sp. IR-811; GU586302	Proteobacteria:Gammaproteobacteria:Pseudomonadales:Moraxellaceae:Acinetobacter	0.399
Otu321	0	0	0	1	uncultured epsilon proteobacterium; pYK04-18B-6; AB235385	Proteobacteria:delta/epsilon subdivisions:Epsilonproteobacteria:environmental samples:unclassified_environmental samples	0.314
Otu322	0	0	1	0	uncultured bacterium; layman_j06; DQ980970	environmental samples:unclassified_environmental samples	0.522
Otu323	0	1	0	0	uncultured Candidatus Rhabdochlamydia sp.; KF-9; EF445478	Chlamydiae/Verrucomicrobia group:Chlamydiae:Chlamydiia:Chlamydiales:Rhabdochlamydiaceae:Candidatus Rhabdochlamydia	0.46
Otu324	0	0	1	0	Rickettsia endosymbiont of Asobara tabida; At-R; FJ603467	Proteobacteria:Alphaproteobacteria:Rickettsiales:Rickettsiaceae:Rickettsieae:Rickettsia	0.455
Otu325	0	1	0	0	uncultured Candidatus Rhabdochlamydia sp.; KF-9; EF445478	Chlamydiae/Verrucomicrobia group:Chlamydiae:Chlamydiia:Chlamydiales:Rhabdochlamydiaceae:Candidatus Rhabdochlamydia	0.471
Otu326	1	0	0	0	uncultured Candidatus Rhabdochlamydia sp.; KF-9; EF445478	Chlamydiae/Verrucomicrobia group:Chlamydiae:Chlamydiia:Chlamydiales:Rhabdochlamydiaceae:Candidatus Rhabdochlamydia	0.545
Otu327	0	0	0	1	uncultured Candidatus Rhabdochlamydia sp.; KF-9; EF445478	Chlamydiae/Verrucomicrobia group:Chlamydiae:Chlamydiia:Chlamydiales:Rhabdochlamydiaceae:Candidatus Rhabdochlamydia	0.401
