# Supplementary material for: Endosymbiont Dominated Bacterial Communities in a Dwarf Spider
Source: PLoS One. 2015 Feb 23;10(2):e0117297. doi: 10.1371/journal.pone.0117297 (PMC4338242; doi:10.1371/journal.pone.0117297)
Supplement: S6 Information — (DOCX) [file pone.0117297.s006.docx]

**Supporting information 6: Detailed analysis strategy of bacterial 16S-V4 and 16S-V3 Illumina HiSeq PE 100bp reads in *Oedothorax gibbosus***

**Unless stated otherwise, analyses were performed by Mothur v.1.26.0 [**[**1**](#_ENREF_1)**]**

**16S-V4 region**

1. Concatenate forward and reverse read via home-made python script (concatenate_nonoverlaps.py), which concatenates the forward and the reverse complemented reverse read (first produce rc of the reverse reads!) and inserts a series of n N's between. Based on the expected length of the amplified V4 region, N was chosen to be equal to 38.
2. Merge datafiles of all samples and transformation into fasta file
3. Trim adapters and primers and demultiplex sequences:
   1. Command:

trim.seqs(fasta=V4_all.fasta, oligos=V4_primersbarcodes.oligos,maxambig=38,pdiffs=0)

- 1. Of the 994.207 sequences, 563.076 (D02a06), 1.034.116 (D03b13), 1.057.021 (DAM) and 1.119.157 (WAL) sequences (3.773.370 in total) were retained.

1. Retain unique sequences:
   1. Command:

unique.seqs(fasta=V4_all.trim.fasta)

Of the 3.773.370 sequences, 340.949 unique sequences were found.

1. Allign seqs with silva bacteria, allowing for reverse complement.
   1. Command: align.seqs(fasta=V4_all.trim.unique.fasta,reference=../16S_Databases/silva.bacteria/silva.bacteria.fasta,processors=4,flip=T)
2. Trim seqs such that only those that start and end at position 15.647 and 23.440 and a length of 207 bp are retained and make new group and names file:
   1. Command:

screen.seqs(fasta=V4_all.trim.unique.align,name=V4_all.trim.names,group=V4_all.groups,start=15647,end=23440,minlength=207)

This resulted in the retainment of 318.794 of the 340.949 sequences

- 1. Resulting files:
- V4_all.trim.unique.good.align
- V4_all.trim.unique.bad.accnos
- V4_all.trim.good.names
- V4_all.good.groups

1. Filter seqs to remove trailing '.'s
   1. Command:

filter.seqs(fasta=V3_clean_bc.trim.unique.good.align,vertical=T,trump=.,processors=2)

- 1. Resulted in an alignment with length 473
  2. Resulting files:
- V4_all.filter
- V4_all.trim.unique.good.filter.fasta

1. Precluster sequences.
   1. Command pre.cluster(fasta=V4_all.trim.unique.good.filter.fasta,name=V4_all.trim.good.names,diffs=3).
   2. This resulted in 133.041 sequences out of the 318.794 sequences.
   3. Resulting files:

- V4_all.trim.unique.good.filter.precluster.fasta
- V4_all.trim.unique.good.filter.precluster.names
- V4_all.trim.unique.good.filter.precluster.map

1. Remove chimeras:
   1. First adjust (filter) the silva bacteria database such that only the alligned region is kept.
   2. Command:

filter.seqs(fasta=../16S_Databases/silva.bacteria/silva.bacteria.fasta, hard=V4_all.filter, processors=2).

Resulting files:

../16S_Databases/silva.bacteria/silva.bacteria.filter.fasta

- 1. Find chimeras by chimera.uchime using names filer as a reference: chimera.uchime(fasta=V4_all.trim.unique.good.filter.precluster.fasta,name=V4_all.trim.unique.good.filter.precluster.names)

This resulted in 36.252 chimeras.

- 1. Chimeras were removed from fasta, names and group file:

remove.seqs(accnos=V4_all.trim.unique.good.filter.precluster.uchime.accnos,fasta=V4_all.trim.unique.good.filter.precluster.fasta,name=V4_all.trim.unique.good.filter.precluster.names, group=V4_all.good.groups)

This resulted in the removal of 36.252 seqs in fasta file and 79.995 seqs in names and group file. This resulted in the retainment of 96.789 sequences.

Output file names:

- V4_all.trim.unique.good.filter.precluster.pick.names
- V4_all.trim.unique.good.filter.precluster.pick.fasta
- V4_all.good.pick.groups

1. Classify sequences:
   1. Command:

classify.seqs(fasta=V4_all.trim.unique.good.filter.precluster.pick.fasta, template=../16S_Databases/silva.bacteria/silva.bacteria.fasta,taxonomy=../16S_Databases/silva.bacteria/silva.bacteria.gg.tax)

- 1. Output files:
- V4_all.trim.unique.good.filter.precluster.pick.gg.taxonomy
- V4_all.trim.unique.good.filter.precluster.pick.gg.flip.accnos
- V4_all.trim.unique.good.filter.precluster.pick.gg.tax.summary

1. Conduct phyllotype analysis:
   1. Command:

phylotype(taxonomy=V4_all.trim.unique.good.filter.precluster.pick.gg.taxonomy,name=V4_all.trim.unique.good.filter.precluster.pick.names)

- 1. Output files:
  - V4_all.trim.unique.good.filter.precluster.pick.gg.tx.list
  - V4_all.trim.unique.good.filter.precluster.pick.gg.tx.sabund
  - V4_all.trim.unique.good.filter.precluster.pick.gg.tx.rabund
  1. Make shared file:

Command: make.shared(list=V4_all.unique.good.filter.precluster.pick.gg.tx.list,group=V4_all.good.pick.groups,label=1).

Output file names:

- V4_all.trim.unique.good.filter.precluster.pick.gg.tx.shared
- V4_all.trim.unique.good.filter.precluster.pick.gg.tx.D02a06.rabund
- V4_all.trim.unique.good.filter.precluster.pick.gg.tx.D03b13.rabund
- V4_all.trim.unique.good.filter.precluster.pick.gg.tx.DAM.rabund
- V4_all.trim.unique.good.filter.precluster.pick.gg.tx.WAL.rabund

1. Classify OUT’s:
   1. Command:

classify.otu(taxonomy=V4_all.trim.unique.good.filter.precluster.pick.gg.taxonomy,name=V4_all.trim.unique.good.filter.precluster.pick.names,list=V4_all.trim.unique.good.filter.precluster.pick.gg.tx.list,label=1)

Output file names:

- V4_all.trim.unique.good.filter.precluster.pick.gg.tx.1cons.taxonomy
- V4_all.trim.unique.good.filter.precluster.pick.gg.tx.1cons.tax.summary

1. Get representative sequence from each phyllotype (method1):
   1. First, all groups at the lowest level ('species') need to be extracted from the ../../Classify/V3_cleaned/V3_clean_bc.trim.unique.good.filter.precluster.pick.gg.tx.list file. To do this, only the first line was written to a seperate file. Next, we selected the first sequence within each phyllotype as a representative sequence. To do this, a substitution was done in vi with the following command:

sed '1,$s/\([^,]HWI[^,]*\)[^\t]*/\1/g' V4_all.trim.unique.good.filter.precluster.pick.gg.tx.list > V4_all.trim.unique.good.filter.precluster.pick.gg.tx.repseq.accnos

This file was edited (remove headings and transposed in MS Excel) and saved as: V4_all.trim.unique.good.filter.precluster.pick.gg.tx.repseq.accnos

- 1. Next, a fasta file was generated by selecting only those sequences whose names are in the accnos file: get.seqs(accnos=V3_clean_bc.trim.unique.good.filter.precluster.pick.gg.tx.repseq.accnos,fasta=../../Trimmed/V3_cleaned/V3_clean_bc.trim.fasta)
  2. The resulting sequences are in the file:

“V4_all.trim.pick.fasta”

1. Get representative sequence from each phyllotype (method2):
   1. First, select the sequences of a particular taxonomy: e.g. get.lineage(taxonomy=V4_all.trim.unique.good.filter.precluster.pick.gg.taxonomy,fasta=V4_all.trim.fasta,name=V4_all.trim.unique.good.filter.precluster.pick.names,group=V4_all.good.pick.groups,taxon=Bacteria;Proteobacteria;Alphaproteobacteria;Consistiales;Rickettsiales;spotted_fever_group;)
   2. Select most abundant sequence from this fasta file:

sort V4_all.rickettsia.fasta | uniq -c | sort -g -r | head -n 1

**16S-V4 region**

1. Concatenate via FLASH [[2](#_ENREF_2)]
2. Merge datafiles (‘V3_all.fastq’)
3. Transform to fasta file (‘V3_all.fasta’)
4. Remove 18s contaminants with a standalone version of Deconseq [[3](#_ENREF_3)], using the 18S sequence of JN816711 as database to remove and preliminary screening of bacterial data as database to keep. This resulted in the files ‘V3_clean.fasta’ and ‘V3_cont.fasta’ and ‘V3_both.fasta’ with 994.207, 1.207.092 and 118 sequences respectively.
5. Trim sequences:
   1. Make oligo's file. Because reads are both forward and reverse strands, both original as well as rc of the primers should be provided. Also barcodes and their sample ID were provided.
   2. Trim sequences by means of the command:

mothur > trim.seqs(fasta=V3_clean_bc.fasta, oligos=V3_primersbarcodes.oligos,maxambig=0,pdiffs=2)

- 1. Of the 994.207 sequences, 443.285 and 445.806 sequences (889.091 in total) were retained for D02a06 and D03b13 respectively and 105.116 sequences were discarded. Trimmed sequences are in ‘V3_clean_bc.trim.fasta’; groups are in ‘**V3_clean_bc.group**’ and discarded sequences are in ‘V3_clean_bc.scrap.fasta”

1. Make unique sequences:
   1. Of the 994.207 sequences, 41.171 unique sequences were found.
   2. Unique sequences in /Unique/V3_cleaned/V3_clean_bc.trim.unique.fasta
   3. Names in /Unique/V3_cleaned/V3_clean_bc.trim.names
2. Allign seqs with silva bacteria, allowing for reverse complement: align.seqs(fasta=V3_clean_bc.trim.unique.fasta,reference=../../16S_Databases/silva.bacteria/silva.bacteria.fasta,processors=1,flip=T)
3. Trim seqs such that only those that start at position 6428 are retained and make new group and names file:
   1. screen.seqs(fasta=V3_clean_bc.trim.unique.align,name=../../Unique/V3_cleaned/V3_clean_bc.trim.names,group=../../Trimmed/V3_cleaned/V3_clean_bc.groups,start=6428,end=13125,minlength=135) . This resulted in the retainment of 40.231 sequences
   2. Resulting files:
      - ‘/Alignment/V3_cleaned/V3_clean_bc.trim.unique.good.align’
      - ‘/Alignment/V3_cleaned/V3_clean_bc.trim.unique.bad.accnos’
      - ‘/Alignment/V3_cleaned/V3_clean_bc.trim.good.names’
      - ‘/Alignment/V3_cleaned/V3_clean_bc.good.groups’
4. Filter seqs to remove trailing '.'s
   1. filter.seqs(fasta=V3_clean_bc.trim.unique.good.align,vertical=T,trump=.,processors=2)
   2. Resulting files:

- ‘V3_clean_bc.filter’
- ‘V3_clean_bc.trim.unique.good.filter.fasta’

1. Precluster sequences with the command pre.cluster(fasta=V3_clean_bc.trim.unique.good.filter.fasta,name=../../Alignment/V3_cleaned/V3_clean_bc.trim.good.names,diffs=2). This resulted in 16.995 sequences out of the 40.231 sequences. The resulting files are:
   - ‘/Precluster/V3_cleaned/V3_clean_bc.trim.unique.good.filter.precluster.fasta’
   - ‘/Precluster/V3_cleaned/V3_clean_bc.trim.unique.good.filter.precluster.names’ ‘/Precluster/V3_cleaned/V3_clean_bc.trim.unique.good.filter.precluster.map’
2. Remove chimeras:
   1. First adjust (filter) the silva bacteria database filter.seqs(fasta=../../16S_Databases/silva.bacteria/silva.bacteria.fasta, hard=../../Alignment/V3_cleaned/V3_clean_bc.filter, processors=2)
   2. Find chimeras by chimera.slayer using new reference (../16S_Databases/silva.bacteria/silva.bacteria.filter.fasta): chimera.slayer(fasta=../../Precluster/V3_cleaned/V3_clean_bc.trim.unique.good.filter.precluster.fasta, reference=../../16S_Databases/silva.bacteria/silva.bacteria.filter.fasta,processors=2)
   3. Find chimeras by chimera.slayer using names file as a reference: chimera.slayer(fasta=../../Precluster/V3_cleaned/V3_clean_bc.trim.unique.good.filter.precluster.fasta,name=../../Precluster/V3_cleaned/V3_clean_bc.trim.unique.good.filter.precluster.names,processors=2) . This resulted in 132 chimeras. The output files are:
      - ‘V3_clean_bc.trim.unique.good.filter.precluster.slayer.accnos’
      - ‘V3_clean_bc.trim.unique.good.filter.precluster.slayer.chimeras’
   4. Find chimeras by chimera.uchime using new reference: chimera.uchime(fasta=../../Precluster/V3_cleaned/V3_clean_bc.trim.unique.good.filter.precluster.fasta,reference=../../16S_Databases/silva.bacteria/silva.bacteria.filter.fasta,processors=2). This resulted in 129 chimeras.
   5. Find chimeras by chimera.uchime using names filer as a reference: chimera.uchime(fasta=../../Precluster/V3_cleaned/V3_clean_bc.trim.unique.good.filter.precluster.fasta,name=../../Precluster/V3_cleaned/V3_clean_bc.trim.unique.good.filter.precluster.names). This resulted in 127 chimeras.
   6. Most chimeras were found with chimera-slayer and remove those sequences from fasta, names and group file:
      1. remove.seqs(accnos=../../Chimeras/V3_clean_bc.trim.unique.good.filter.precluster.slayer.accnos, fasta=../../Precluster/V3_cleaned/V3_clean_bc.trim.unique.good.filter.precluster.fasta,name=../../Precluster/V3_cleaned/V3_clean_bc.trim.unique.good.filter.precluster.names, group=../../Alignment/V3_cleaned/V3_clean_bc.good.groups)
      2. This resulted in the removal of 132 seqs in fasta file and 395 seqs in names and group file.
      3. Output file names:

- ‘V3_clean_bc.trim.unique.good.filter.precluster.pick.names’
- ‘V3_clean_bc.trim.unique.good.filter.precluster.pick.fasta’
- ‘V3_clean_bc.good.pick.groups’

1. Classify sequences:
   1. classify.seqs(fasta=V3_clean_bc.trim.unique.good.filter.precluster.pick.fasta, template=../../16S_Databases/silva.bacteria/silva.bacteria.fasta,taxonomy=../../16S_Databases/silva.bacteria/silva.bacteria.gg.tax)
   2. Output files:
      - ‘V3_clean_bc.trim.unique.good.filter.precluster.pick.gg.taxonomy’
      - ‘V3_clean_bc.trim.unique.good.filter.precluster.pick.gg.flip.accnos’
      - ‘V3_clean_bc.trim.unique.good.filter.precluster.pick.gg.tax.summary’
2. Conduct phyllotype analysis:
   1. phylotype(taxonomy=../../Classify/V3_cleaned/V3_clean_bc.trim.unique.good.filter.precluster.pick.gg.taxonomy,name=../../Classify/V3_cleaned/V3_clean_bc.trim.unique.good.filter.precluster.pick.names). Output files:
      - ‘V3_clean_bc.trim.unique.good.filter.precluster.pick.gg.tx.list’
      - ‘V3_clean_bc.trim.unique.good.filter.precluster.pick.gg.tx.sabund’
      - ‘V3_clean_bc.trim.unique.good.filter.precluster.pick.gg.tx.rabund’
   2. make.shared(list=../../Classify/V3_cleaned/V3_clean_bc.trim.unique.good.filter.precluster.pick.gg.tx.list,group=../../Classify/V3_cleaned/V3_clean_bc.good.pick.groups,label=1). Output file names:
      - ‘V3_clean_bc.trim.unique.good.filter.precluster.pick.gg.tx.shared’
      - ‘V3_clean_bc.trim.unique.good.filter.precluster.pick.gg.tx.D02a06.rabund’
      - ‘V3_clean_bc.trim.unique.good.filter.precluster.pick.gg.tx.D03b13.rabund’
   3. classify.otu(taxonomy=../../Classify/V3_cleaned/V3_clean_bc.trim.unique.good.filter.precluster.pick.gg.taxonomy,name=../../Classify/V3_cleaned/V3_clean_bc.trim.unique.good.filter.precluster.pick.names,list=../../Classify/V3_cleaned/V3_clean_bc.trim.unique.good.filter.precluster.pick.gg.tx.list,label=1). Output file names:
      - ‘V3_clean_bc.trim.unique.good.filter.precluster.pick.gg.tx.1cons.taxonomy’
      - ‘V3_clean_bc.trim.unique.good.filter.precluster.pick.gg.tx.1cons.tax.summary’
3. Get representative sequence from each phyllotype:
   1. First, all groups at the lowest level ('species') were extracted from the ../../Classify/V3_cleaned/V3_clean_bc.trim.unique.good.filter.precluster.pick.gg.tx.list file.
   2. Next, we selected the first sequence within each phyllotype as a representative sequence. To do this, a substitution was done in vi with the following command:

1,$s/\([^,]HWI[^,]*\)[^\t]*/\1/g

This file was edited (remove headings and transposed in Excel) and saved as: ‘V3_clean_bc.trim.unique.good.filter.precluster.pick.gg.tx.repseq.accnos’

- 1. Next, a fasta file was generated by selecting only those sequences whose names are in the accnos file: get.seqs(accnos=V3_clean_bc.trim.unique.good.filter.precluster.pick.gg.tx.repseq.accnos,fasta=../../Trimmed/V3_cleaned/V3_clean_bc.trim.fasta)
  2. The resulting sequences are in the file:

‘V3_clean_bc.trim.pick.fasta’

1. Get representative sequence from each phyllotype (method2):
   1. First, select the sequences of a particular taxonomy: get.lineage(taxonomy=../../Classify/V4_all.trim.unique.good.filter.precluster.pick.gg.taxonomy,fasta=V4_all.trim.fasta,name=V4_all.trim.unique.good.filter.precluster.pick.names,group=V4_all.good.pick.groups,taxon=Bacteria;Proteobacteria;Alphaproteobacteria;Consistiales;Rickettsiales;spotted_fever_group;)
   2. Select most abundant sequence from this fasta file in bash:

sort V4_all.rickettsia.fasta | uniq -c | sort -g -r | head -n 1

- 1. Replace these sequences with those in ../../Phyllotypes/V3
     _cleaned/V3_clean_bc.trim.pick.fasta and save as

../../Repseqs/V3_cleaned/V3_clean_bc.repseqs.fasta

1. Select all sequences per species:
   1. 1,$s/^[^H]*\(H[^\t]*\)\t\(H[^\t]*\)\t\(H[^\t]*\)\t\(H[^\t]*\)\t\(H[^\t]*\)\t\(H[^\t]*\)\t\(H[^\t]*\)\t\(H[^\t]*\)\t.*/\7/g

**References**

1. Schloss PD, Westcott SL, Ryabin T, Hall JR, Hartmann M, et al. (2009) Introducing mothur: Open-Source, Platform-Independent, Community-Supported Software for Describing and Comparing Microbial Communities. Applied and Environmental Microbiology 75: 7537-7541.

2. Magoc T, Salzberg SL (2011) FLASH: fast length adjustment of short reads to improve genome assemblies. Bioinformatics 27: 2957-2963.

3. Schmieder R, Edwards R (2011) Fast Identification and Removal of Sequence Contamination from Genomic and Metagenomic Datasets. PLoS One 6.
